# Supplementary material for: Effects of a personalized or generic three-dimensional tumoral kidney model on patient experience and caregiver-patient interactions, before and after partial nephrectomy, a randomized trial (Rein 3D Print Personalize—UroCCR 114)
Source: PLoS One. 2025 Aug 18;20(8):e0323515. doi: 10.1371/journal.pone.0323515 (PMC12360608; doi:10.1371/journal.pone.0323515)
Supplement: S1 File — (PDF) [file pone.0323515.s001.pdf]

Direction des Grands Programmes d'Investissement de l'Etat
Affaire suivie par : Jean-Claude Dussaule
Mail : jean-claude .dussaule@agencerecherche.fr
Nos réf : 2021-531

Le Président-directeur général de l'Agence Nationale de la Recherche

A

Monsieur Jean-Christophe BERNHARD
Service de Chirurgie Urologique et Transplantation Rénale
1 Place Amélie Raba Léon
33076 BORDEAUX

Paris, 10 DEC. 2021

Objet : Appel à projets Recherche Hospitalo-Universitaires Vague 5 (RHU5)

Monsieur Jean-Christophe BERNHARD,

En réponse à l'appel à projets "Recherche Hospitalo-Universitaire en santé" (RHU5) lancé dans le cadre du troisième programme "Investissements d'Avenir" par l'Agence Nationale de la Recherche, vous avez soumis le projet Digital Urology 3D.

Cet appel à projets a recueilli 89 propositions éligibles qui ont fait l'objet d'une évaluation indépendante par un jury international et dont 25 ont été auditionnés. A l'issue de ce processus de sélection RHU5, le jury a proposé pour financement dix-sept projets qui ont reçu l'aval du comité de pilotage.

J'ai l'honneur et le plaisir de vous informer que votre projet Digital Urology 3D a été retenu pour financement. Vous trouverez le rapport final et collectif du jury annexé à ce courrier.

Le montant de la subvention et les conditions à remplir en vue de la signature de la convention de préfinancement, puis de la convention définitive, vous seront précisés prochainement par une décision signée par le Premier Ministre.

Je vous prie d'agréer, Monsieur, l'expression de mes salutations distinguées.

Thierry Damerval

La présente décision peut faire l'objet d'un recours contentieux auprès du tribunal administratif de Paris dans un délai de deux mois à compter de sa notification.

Major State Investment Programs Department

Chairman and CEO, French National Research Agency (ANR)

To

Mr. Jean-Christophe Bernhard
Department of Urological Surgery and Renal Transplantation
1 place Amélie Raba Léon
33076 Bordeaux FRANCE

Mr Jean-Christophe BERNHARD,

In response to the call for projects for “University Hospital Research in Health” (RHU 5) launched as part of the 3rd “Investissement d'Avenir” program by the French National Research Agency, you have submitted the Digital Urology 3D project.

This call for projects attracted 89 eligible proposals, all of which were independently assessed by an international jury, and 25 of which were auditioned. Following this RHU5 selection process, the jury proposed seventeen projects for funding, which were approved by the steering committee.

I am pleased to inform you that your Digital Urology 3D project has been selected for funding. You will find the jury's final and collective report appended to this dossier.

The amount of the grant and the conditions to be met to sign the pre-financing agreement, and then the final agreement, will be specified shortly in a decision signed by the Prime Minister.

Digital Urology 3D ranked a

Introduction and brief description of the project

The project is related to transform standard of care in kidney cancer (KC) by proposing a change of paradigm in the way KC surgery is seen, performed and trained towards a more personalized, safe and patient-centered procedure.

The project belongs to an area which is called digital urology and the project is based on a cooperation of academic / clinical centers with companies and networks in the area (Kidney Cancer UroCCR). The project will result in the improvement of clinical and technical knowledge in the field, collection of clinical evidence on new image guided surgical techniques and IT tools such as the AR surgery tool, a communication kit, 3D soft and multi material printer and several training models. The project can be expanded in the future to cover surgery of other soft organs. The project includes several innovative elements and combines existing work and knowledge and most importantly covers most of the issues related to IT based Kidney Cancer surgery.

Evaluation

1) Originality, Feasibility, Credibility

Project is aiming to bring to routine the virtual 3D and 3D printing technologies, this is novel, especially for kidneys. The project is organised in three technical work packages and two horizontal work packages addressing management, dissemination and exploitation. The competitive advantages of the project are related to the use of VR for image guided surgery, the use of AR and in the development of soft (silicone) 3D printing.

The feasibility of this project is strictly related with the feasibility of the engineering development of the different aspect presented, starting from the realization of 3D models to AR platform and 3D silicon perfused models. This is the crucial step: once the engineers will be able to offer to the surgeons an adequate product for surgeons' needs in the theatre, the clinical validation is relatively easy.

Weaknesses

Still project has to prove usefulness for the healthcare system and exploitation of the modules to be developed, that it is efficient and it can be included in the clinical practice. This needs further validation and metrics which must be instructed, including HTA and health economics.

2) Scientific quality

The scientific quality of the proposal is very good and well documented. It combines several IT techniques and clinical studies to collect the data and validate the outcomes. The organization of work presented in the Fig. in page 9 covers all aspects and will lead to the objectives proposed. The work is tackled technically with existing methodologies but the outcome will be novel or by purely technically innovative manner (3D printer) and related questions are addressed. The clinical studies supporting the work are well organized.

Weaknesses

More technical details in the implementation are needed.

The metrics for the validation must be more specific.

The accuracy of the models and the feasibility, accuracy and safety of AR surgery should be specified.

3) Global impact of the project

The focus of the project being on evidence but also IT tools characterises the impact in both the development of new tools, but also to a better healthcare system, which will be more efficient and more cost effective.

The presented project is ambitious and describes a very interesting potential development of 3D guided surgery during the next five years. The potential impact on urological surgery is impressive both for the patients and for the surgeons.

The project follows the French Government objective related to the patient safety and efficiency of healthcare systems, which is a global trend now worldwide. The focus of the project being on evidence and IT tools characterizes the impact in both the development of new tools, but also to a better healthcare system, which will be more efficient and more cost effective.

Weaknesses

Some KPIs are mentioned, but those must be more detailed and must be included in the project per IT module, or per workpackage.

It is not described how the impact will be proven.

This kind of technology will be available only in high volume center that have the possibility to pay the models and to have the robotic console to perform AR surgery.

4) Quality of the consortium

PI's research areas of interest include conservative robotic kidney cancer surgery, virtual navigation, 3D pre-operative models for surgical planning and simulation, biomarkers for kidney cancer. He is national coordinator and PI of multiple clinical research studies.

The consortium is composed of 8 partners with long term experience in the fields they have to support. The expertise is not only academic / clinical but also comes from companies leaders in their field, with good records with previous projects. The skills are complementary and it is good for the project to include a regulatory expert for the market access. The participation in networks and previous experience gives added value to the work plan of the project. The same applies for the pre-existing support platforms to be used in the project.

Weaknesses

It is not clear how the whole concept for digital surgery in urology will be translated into the clinical practice.

A lot of different actors will work together. Each single actor has specific requisites and needs (i.e. technical, clinical, legal, ethics..., etc.).

5) Methodology

The project is organised in three technical workpackages, and deliverables, and milestones are provided. The roles are clear as well as the information flow, and workflow between the WPs is well described. The same applies for the two horizontal workpackages.

The methodology appears to be solid with level 1 studies planned. For each study proposed the outcomes are clearly presented and as well the methodology. The measurement tools are declared by the authors.

The project is organized in three technical work packages, and deliverables, and milestones are provided. The roles are clear as well as the information flow, and workflow between the WPs is well described. The same applies for the two horizontal workpackages. It is good that the project will hire a project manager to perform the day-to-day work and follow all activities. The risks are mentioned as well as mitigation plans.

Weaknesses

It is not clear what are the metrics and tools that can be used to prove the efficiency of each one of the developed models. For making the proposed approach to clinical practice evidence must be collected and that must be clear in the proposed clinical trials. The contingency plans for the risks in some cases are not convincing.

There is no evaluation of the accuracy of 3D models and their overlapping.

Underestimated sample sizes here and there.

Not defined or not matched statistical analyses for the raised hypotheses.

6) Adequacy between project and resources

The resources needed are clearly described in the proposal and the work assigned to each partner is clearly described. The internal resources are also described and it seems that all aspects of the work promised can be covered.

The allocation of the resources appears to be adequate. There is a good mix between public university and private industries. The technical development of the different product presented (3D models, AR platform etc.) will occupy most of the resources respectively.

Weaknesses

The costs requested for the regulatory study and the marketing study are underestimated and that it is the major weakness of the project. Market access is mentioned, but processing of evidence, processing of the clinical trials outcomes, HTA cannot be performed within this amount of budget given the prices that consulting companies in the field request.

General comments

1) Strengths

Project is aiming to bring to routine the virtual 3D and 3D printing technologies, this is novel, especially for kidneys. The project is organised in three technical workpackages and two horizontal workpackages addressing management, dissemination and exploitation. The competitive advantages of the project are related to the use of VR for image guided surgery, the use of AR and in the development of soft (silicone) 3D printing.

The feasibility of this project is strictly related with the feasibility of the engineering development of the different aspect presented, starting from the realization of 3D models to AR platform and 3D silicon

perfused models. This is the crucial step: once the engineers will be able to offer to the surgeons an adequate product for surgeons' needs in the theatre, the clinical validation is relatively easy.

The scientific quality of the proposal is very good and well documented. It combines several IT techniques and clinical studies to collect the data and validate the outcomes.

The focus of the project being on evidence but also IT tools characterises the impact in both the development of new tools, but also to a better healthcare system, which will be more efficient and more cost effective.

PI's research areas of interest include conservative robotic kidney cancer surgery, virtual navigation, 3D pre-operative models for surgical planning and simulation, biomarkers for kidney cancer. He is national coordinator and PI of multiple clinical research studies.

The consortium is composed of 8 partners with long term experience in the fields they must support. The expertise is not only academic / clinical but also comes from companies leaders in their field, with good records with previous projects. The skills are complementary and it is good for the project to include a regulatory expert for the market access. The participation in networks and previous experience gives added value to the workplan of the project. The same applies for the pre-existing support platforms to be used in the project.

2) Weaknesses

Still project must prove usefulness for the healthcare system and exploitation of the modules to be developed, that it is efficient and it can be included in the clinical practice. This needs further validation and metrics which must be instructed including HTA and health economics.

More technical details in the implementation are needed.

The metrics for the validation must be more specific.

The accuracy of the models and the feasibility, accuracy and safety of AR surgery should be specified.

Horizontal combination of metrics among the WPs is also missing, to provide with the outcome which will be the paradigm shift evaluation in the digital urology.

Some KPIs are mentioned, but those must be more detailed and must be included in the project per IT module, or per workpackage.

It is not described how the impact will be proven.

This kind of technology will be available only in high volume center that have the possibility to pay the models and to have the robotic console to perform AR surgery.

A lot of different actors will work together. Each single actor has specific requisites and needs (i.e., technical, clinical, legal, ethics.... etc.).

3) Conclusion / Recommendation

Project is aiming to bring to routine the virtual 3D and 3D printing technologies, this is novel, especially for kidneys. The project is organised in three technical workpackages and two horizontal workpackages addressing management, dissemination and exploitation. The competitive advantages of the project are related to the use of VR for image guided surgery, the use of AR and in the development of soft (silicone) 3D printing.

The feasibility of this project is strictly related with the feasibility of the engineering development of the different aspect presented, starting from the realization of 3D models, to AR platform and 3D silicon perfused models. This is the crucial step: once the engineers will be able to offer to the surgeons an adequate product for surgeons' needs in the theatre, the clinical validation is relatively easy.

The scientific quality of the proposal is very good and well documented. It combines several IT techniques and clinical studies to collect the data and validate the outcomes.

During the hearing consortium explained usefulness for the healthcare system and exploitation of the modules and efficacy what will be included in the clinical practice. Also, validation and metrics including HTA and health economics are better explained.

The accuracy of the models and the feasibility, accuracy and safety of AR surgery are provided during hearing phase.
