# Supplementary material for: Effects of a personalized or generic three-dimensional tumoral kidney model on patient experience and caregiver-patient interactions, before and after partial nephrectomy, a randomized trial (Rein 3D Print Personalize—UroCCR 114)
Source: PLoS One. 2025 Aug 18;20(8):e0323515. doi: 10.1371/journal.pone.0323515 (PMC12360608; doi:10.1371/journal.pone.0323515)
Supplement: S2 File — (PDF) [file pone.0323515.s002.pdf]

Troisième Programme « Investissements d'avenir »
Appel à projets « Recherche Hospitalo-Universitaire en santé 5e édition »
Jury RHU5 - Edition 2021

Third " Future Investments " Program
Request for proposals "University Hospital Research in Health 5th edition" RHU5 Jury - Edition 2021

Président

Jürgen BECK
PAION AG
Aix-la-Chapelle, Allemagne

Vice-Présidentes

Deborah BURKS
CIBERDEM, Centro de Investigacion Prıncipe Felipe
Valencia, Espagne

Marie-Pierre GAGNON
Université de Laval
Québec, Canada

Carolyn McGREGOR
Institute of Technology, Université d'Ontario
Oshawa, Canada

Membres

Marina BERENGUER
University of Valencia,
Valencia, Espagne

Jean-Pierre BIZZARI
Consulting
New York, États-Unis

Johannes BLATTER
SRH Berufsbildungswerk
Schriesheim, Allemagne

Christian BUCHEL
University Medical Centre Hamburg-Eppendorf
Hamburg, Allemagne

Rafael CANTON
University Hospital Ramón y Cajal
Madrid, Espagne

Yves CARIOU
YC-HCS consultancy
Bruxelles, Belgique

Mina CHUNG
Cleveland Clinic Lerner
Cleveland, États-Unis

Pál CZOBOR
Semmelweis University
Budapest, Hongrie

Olivier DANOS
Regenxbio
New York, États-Unis

Dave DWORACZYK
Bryn Pharma
Beverly Hills, États-Unis

Luca FALCIOLA
DROIA Oncology Ventures
Meise, Belgique

Nenad FILIPOVIC
Kragujevac University
Kragujevac, Serbie

Jörg HAGER
Nestlé Institute of Health Sciences SA,
Lausanne, Suisse

Rita HORVATH
University of Cambridge
Cambridge, Royaume-Uni

Mihaly IMRE
University of Medicine and Pharmacy,
Tirgu Mures, Roumanie

Eric JOHNSON
University of California
Los Angeles, Etats-Unis

Paolo MADEDDU
Bristol University
Bristol, Royaume-Uni

António MARINHO
Centro Hospitar Do Porto, E.P.E.
Porto, Portugal

Membres

Adele MARSHALL
Queen's University Belfast
Belfast, Royaume-Uni

Isabelle MEYTS
Universitair Ziekenhuis Leuven
Leuven, Belgique

Luke MOORE
Chelsea & Westminster
NHS Foundation Trust
Londres, Royaume Uni

Mohandas NARLA
New York Blood Center
New York, États-Unis

Steve PASCOLO
spRNA GmbH,
Zurich, Suisse

Bruno PEAULT
University of Edinburgh
Edimbourg, Royaume-Uni
University of California
Los Angeles, États-Unis

Maria PUFULETE
Bristol Medical School (THS)
Bristol, Royaume-Uni

Werner REIN
Theranexus SA
Paris, France

Bachir TAOULI
School of Medicine at Mount Sinai
New York, États-Unis

Hoda Tawfik
Magforce AG, Nanomedicine
Berlin, Allemagne

Turi STEFANO
San Raffaele Ospedale
Milan, Italie

Marjolein VAN EGMOND
Université d’Amsterdam
Amsterdam, Pays-Bas

Kristin VERBEKE
Faculty of Medicine
Leuven, Belgique

Roel WILLEMZE
Leiden University Medical Center
Leiden, Pays-Bas

Andreas ZIEGLER
StatSol
Lübeck, Allemagne
