# Supplementary material for: Effects of a personalized or generic three-dimensional tumoral kidney model on patient experience and caregiver-patient interactions, before and after partial nephrectomy, a randomized trial (Rein 3D Print Personalize—UroCCR 114)
Source: PLoS One. 2025 Aug 18;20(8):e0323515. doi: 10.1371/journal.pone.0323515 (PMC12360608; doi:10.1371/journal.pone.0323515)
Supplement: S3 File — (PDF) [file pone.0323515.s003.pdf]

INVESTMENTS FOR THE FUTURE

HOSPITAL-UNIVERSITY RESEARCH IN HEALTH (RHU)

2021 Edition, fifth call

Call for proposals closing date

01 July 2021 at 1 pm (Paris time)

Call for proposals publication address

https://anr.fr/fr/detail/call/recherche-hospitalo-universitaire-en-sante-rhu-vague-5-2021-1/

KEY WORDS

Translational research in health, clinical trials and research, cohorts of patients, personalized medicine, healthcare technologies, therapeutics, diagnostics, public-private partnerships, technology transfer, development and valorisation, medication, advanced therapy medicinal products, medical devices, antibiotic resistance, biotherapy, biomarkers, technological platforms, bioinformatics, pathophysiology, systems biology.

SUMMARY

This present call for proposals extends the objectives of call "*Hospital-University research in health*" (RHU). Its goal is to stimulate innovative and large-scale research projects in the area of health. The projects are expected to be of translational nature. Insofar, they will present a strong potential for rapid transfer to the practice of care, industrial production or the implementation of public policies. These projects will mandatorily be coordinated by teams of university hospital located inside a health establishment (CHU, CLCC, etc.), and be involving one or more companies with a view to developing health solutions (medical devices, therapy products, diagnostic tools…). If relevant, the proposed projects will potentially be involving one or more local authorities. Proposals may be backed by projects funded under the Investments for the Future Programme, but must demonstrate a real scientific or medical gain, thematic coherence and a new ambition justifying these reconciliations. Whatever the case, the *hospital-university research in health* action is intended to support research projects and not structures.

.

IMPORTANT DATES

DEADLINE FOR SUBMISSION OF PROPOSALS

Electronic submission of the application and supporting documents is mandatory (cf. §5 Conditions of submission) before:

01 July 2021 at 1 pm (PARIS TIME)

Via the website:

https://investissementsdavenir.agencerecherche.fr/RHU-V5/

CONTACTS

FOR ANY QUESTIONS - rhu@agencerecherche.fr

IN CHARGE OF ACTION

Jean-Claude Dussaule jean-claude.dussaule@agencerecherche.fr

CORRESPONDENT

Gabriel Matherat gabriel.matherat@agencerecherche.fr

Please read carefully and thoroughly this document and the "regulations on conditions for the allocation of funding project application in hospital and academic research in health" prior to submitting a proposal for a research project.

CONTENTS

1. Context and objectives of the call for proposals 4

1.1. Context 4
1.2. Call for proposals objectives 5

2. Scope of the call for proposals 5

2.1. Extent 5
2.2. Partners 6
2.3. Mission and specificities of the projects 6
2.4. Specific provisions 7

3. Evaluation of the submitted projects 7

3.1. Acceptability criteria... 8
3.2. Eligibility criteria 9
3.3. Evaluation criteria 10
3.4. Important recommendations 11

4. General provisions for the funding....................................... 12

4.1. Funding 12
4.2. Consortium agreements.. 13
4.3. Other provisions 13

5. Conditions of submission.. 14

5.1. Content of the submission dossier.. 14
5.2. Submission procedure 15
5.3. Advice for submission... 15

6. Appendix 16

6.1. Definitions relating to the project organization 16
6.2. Definitions relating to the structures 16
6.3. Definitions relating to the different categories of research......... 17
6.4. Other definitions.. 18

1. Context and objectives of the call for proposals

1.1. Context

The *Hospital-University research in health* action is supporting a major effort to structure teams of health research. Within the first "Investments for the future" program in 2011-2012, this effort resulted in the "Academic Hospital Institutions" action which selected 6 projects classified A (IHU)[1], 6 projects classified B (PHUB) and 2 hospital-university oncology research clusters (PHUC)[2]. These projects closely associate activities of fundamental research, health translational research, clinical research, training, health care and valorisation in a specific therapeutic area (cardiology, neuroscience, metabolism, rare diseases, infectious diseases, technologies for health, oncology, haematology, immunology, transplantation).

This structuring effort was strengthened in 2012 with the creation of new entities recognized through a tripartite agreement between a university hospital (CHU), a university and INSERM (French National Institute of Health and Medical Research) or another institution member of Aviesan alliance: hospital-university departments (DHU) and hospital-university federations (FHU), which today extend throughout France. During the two first editions, the call of proposals *Hospital-University Research in Health* (RHU) contributed to the DHU/FHU structuring. This fifth call, like the fourth and the third ones, is opened to any hospital-university team.

With this fifth RHU call of proposals, the Government keeps to contribute to this structuration effort of the research teams in health. Indeed, while boosting innovation and facilitating hospital-university research in health, the RHU action aims at creating an ecosystem leading to a sustainable and fruitful partnership between academic and industrial teams. It also plans to promote the transfer of innovation into daily medical practice. Previous editions of the RHU call of proposals have confirmed that the French research was able to foster the emergence of large translational research project of excellent quality, associating research activities and healthcare activities, with the support from industrial partners.

The candidates in this this fifth RHU call have to demonstrate recognized expertise in preclinical research, pathophysiology, creation and study of cellular or animal model systems, identification and validation of therapeutic targets and biomarkers. They must have developed high-level know-how in constituting and exploiting strongly documented and highly stratified cohorts of patients, linked to biological resources centres and rapidly available for clinical trials. The projects must be worn by teams acknowledged for the excellence of their methodological approaches and for the conduct of clinical trials. They offer hospital structures specifically dedicated to research and have access to technologic platforms in biology and medical imaging that ensure the production of data whose quality is guaranteed by standardized procedures. They have also access to information systems that ensure the traceability of the data collected and interoperability within the network and with other

---

[1] http://www.agence-nationale-recherche.fr/investissementsdavenir/documents/ihu-selection-2010.pdf
[2] http://www.agence-nationale-recherche.fr/investissementsdavenir/documents/2011/phuc-selection-2011.pdf

collaborating centres. Finally, they propose a single window for industrial partnerships in order to ensure rapid transfer of scientific innovations into healthcare practices.

1.2. Call for proposals objectives

The *hospital-university research in health* program aims at supporting translational health research projects or clinical research projects. These projects will rely on fundamental research in biology, epidemiology, social sciences or health economics, and extend them to achieve benefits in patient care, improved understanding of diseases, more effective and better-tolerated treatments, and progress in healthcare system performance.

The projects submitted may associate the development of medical devices, biomarkers for therapeutic or diagnostic application, biological and technological platforms, bioinformatics or biology system platforms. Projects regarding personalized medicine and innovative therapeutics, incorporating the use of connected consumer items in the area of health, or proposing new protocols or treatment management modalities for patients, may also be submitted.

The proposed programs must have a socio-economic impact, especially through the improvement of medical practices and healthcare system performance or the decrease in healthcare costs. The projects should include a recovery target and/or transfer of technologies. They should be able to attract a significant number of projects from private partners and, as far as possible, include a partnership with a competitiveness cluster. The proposals should demonstrate a research partnership dynamism in the healthcare and life sciences areas, and thus better integrate research, education and care into the major health challenges.

Large-scale research projects with strong potential for transfer to industry and/or society are awaited. The most ambitious projects will be eligible for funding of 4 to 10 million Euros with a completion deadline of 5 years. Only new original projects which have not previously been funded by ANR will be considered. The leadership by hospital-university entities aims at structuring the health research around themes of excellence and removing barriers between the structures (public institutions/private enterprises/local authorities). The association of enterprises and/or local authorities will ensure an economic and/or social return from the supported projects. At local, regional, national and international level, the candidates will have to show their strategic and structuring role in their hospital-university environment and medical specialty. They will have to prove their ability to conduct and sustain innovative and high-quality research matching top world standards. The attractiveness and the dynamics of the submitted projects will be assessed according to their total cost which should be 3 times higher than the requested funding.

2. Scope of the call for proposals

2.1. Extent

In all cases, the objective is to support research projects and not to support structures. Only projects submitted by a hospital-university team will be considered. On condition that they

demonstrate coherence and added-value in scientific terms, projects associating a hospital-university teams with others academic partners are encouraged, as are projects capitalizing on other achievements from the previous Investments for the Future program (Labex, Equipex, Cohorts, Biology-health Infrastructures, Demonstrators, Nanobiotechnologies and Bioinformatics programs, Technology Research Institute).

The projects must necessarily include at least one enterprise with a view to developing health solutions (medical devices, therapy products, diagnostic tools…). If relevant, projects will potentially be involving one or more local authorities.

The team laureates of the previous calls for proposals can submit new projects. However, projects must explicitly be coordinated by different scientific and technical managers.

These projects should ambition to be included in the strategic agenda for research France Horizon 2020, with the perspective of European visibility through European partnerships established by the teams.

A dynamic force of translational research must be at the core of each project: from bench to bed and from bed to bench. The candidates will have to demonstrate the existence of carefully phenotyped active patient files or cohorts, associated with biological resources centres, and consistent with the proposed topic and the scientific project. The projects will thus involve clinicians and researchers in all the activities.

The candidates will have to assemble a critical mass of researchers and clinicians as well as a central core of resources, equipment and competencies suitable for the ambition of the submitted project. It is a question of funding projects which can be accomplished in the contract period.

2.2. Partners

The proposed projects must be partnership projects involving at least one university hospital or healthcare institution, at least one research organization (university, national organism… cf. definitions §2.6) and at least one enterprise. The projects may be limited to a principal site or possibly be associated with satellite sites, but must necessarily ensure scientific and medical coherence.

The projects may involve foreign partners (Europe and outside Europe), although foreign partners will not be financed by ANR.

Only one partner, the coordinator institution specified in the project, will sign the grant agreement with ANR. This partner will be in charge of managing the funding and will sign subcontracts with its partner institutions if necessary.

2.3. Mission and specificities of the projects

In terms of research activities, the submitted projects should be capable of closely combining fundamental and clinical research, and to this end must be able to:

- formulate research concerns issued from health care and to explore their fundamental

aspects,

- bring results supporting the main hypothesis,
- validate the proofs of concept and perform their clinical assessment,
- extract new preventive, diagnostic or therapeutic strategies,
- ensure their medical, social and economic evaluation,
- conclude partnerships with the private sector,
- create a consortium of researchers able to attract funds from national, European or international research institutions,
- contribute to the research training of health professionals,
- ensure the dissemination of discoveries and innovative practices to the professionals, the patients and the public.

2.4. Specific provisions

During the selection process, the ANR will check the financial soundness of the partner enterprises.

Benefit for patients and socio-economic spin-offs will be considered as well as ethical aspects and the acceptability of the submitted projects.

3. Evaluation of the submitted projects

The projects will be selected by an international and independent jury, mainly composed of foreign members (recognized in the scientific, medical and technology sectors and of personalities from the economic world) who collectively gather experience in research, training, clinical and translational research, and in the evaluation of healthcare systems.

The main steps of the selection process are as follows:

- Evaluation of the project acceptability by the ANR, according to the criteria described in §3.1,
- Evaluation of the project eligibility by the Evaluation Committee, according to the criteria described in §3.2;
- Evaluation of the projects by the Evaluation Committee with, if appropriate, use of external expertise[3] and, if deemed necessary by the Evaluation Committee, after a pre-selection and an audition of the leaders of the pre-selected projects;
- Handing over of the Evaluation Committee report to the steering committee[4] including: i) a set of grades from A to E (or equivalent) for each project, according to the criteria described §3.3; ii) a motivated list of projects considered by the Evaluation

[3] The external experts are designated by the jury and give a written advice on the projects
[4] The steering committee is the body as referred to in paragraph 2.4 of the Convention State – ANR governing the present call for proposals. It is chaired by the Minister in charge of higher education and research, or his representative. The members of the steering committee are independent from the projects.

Committee as not recommended for funding due to insufficient quality with regard to at least one of the criteria or its overall perception of the project; iii) a motivated list of projects considered as potentially fundable and subject, where relevant, to modifications that the Evaluation Committee will indicate as recommendations;

- The steering committee: i) on the basis of the international Evaluation Committee's report, proposes to the general secretary for investment the designation of the beneficiaries and the corresponding amounts: the final decision is taken by the Prime Minister; ii) asks the ANR President and General Manager to sign the ANR/beneficiaries conventions which detail the mutual obligations of the parties; iii) ensures the partial or total grant payment under the conditions provided for by the conventions, after decision from the Prime Minister.

The persons participating in the evaluation of the projects, particularly the Evaluation Committee and the solicited experts, will have to adhere to the ANR[5] ethics charter. ANR will notably ensure the respect of confidentiality and the absence of any relation or conflict of interest. In case of any infringement, the ANR reserves the right to take any measure it considers necessary to remedy the situation. The ANR ethics charter is available on its website. Operational and organizational arrangements of the Evaluation Committee are described in the documents available on ANR website.

The composition of the Evaluation Committee will be posted on the publication website of the call for proposals after the evaluation process.

3.1. ACCEPTABILITY CRITERIA

IMPORTANT

The dossiers that do not meet the acceptability criteria will not be submitted to the Evaluation Committee and in no case will be eligible for funding.

1) The submission dossier must be filed on the full ANR submission site before the date and time of closure of the call for proposals. Furthermore, administrative and financial document and letters of commitment signed and scanned are to be filed on the ANR submission website at the date and time indicated on page 2.

2) The scientific document has to be under unprotected PDF format and must not exceed 40 pages (minimum font size: 11). Any scientific supporting document exceeding 40 pages will automatically render the project inadmissible. The scientific document has to be supplemented by:

5 http://www.agence-nationale-recherche.fr/CharteDeontologieSelection et http://www.agence-nationale-recherche.fr/missions-et-organisation/qualitedeontologie/politique-d-ethique-et-d-integrite-scientifique/

a) an annex precisely describing preliminary data and publications which support the proof of concept on which the project is based. Maximum of 5 pages (minimum font size: 11) ;

b) an annex devoted exclusively to the description of the preclinical and clinical trial methodology proposed in the project and necessary for the scientific evaluation. Each clinical trial planned in the project must be described on one page maximum, all compiled into a single annex (minimum font size: 11) ;

c) an annex describing in 3 pages maximum (minimum font size: 11) the sharing principles from the impact of the project between partners (intellectual property, know-how, revenue…).

3) Project must not last for more than 60 months.

4) The amount of the requested grant must be between €4 million and €10 million.

5) The project must have a significant funding leveraging effect. The total cost of the projects must be at least 3 times higher than the amount of funding granted.

6) This call for proposals is opened only to partnership-based research projects. The project must include at least one partner from each of the following categories (cf. definitions §6.2):
   - Research organization (university, national organism…),
   - Healthcare institution,
   - Enterprise.

7) The coordinator institution must be a hospital-university centre or a research organization (university, national organism, scientific cooperation foundation, etc.) (cf. definitions §6.2).

8) The scientific project leader has to belong to a hospital-university team. ANR reserves the right to request any documents it considers useful to check that this acceptability criterion is satisfied.

9) An entity may propose and coordinate several projects. Winner establishments of the RHU first calls for proposals can submit new projects. <u>However, these new projects must be led by different scientific and technical managers.</u>

3.2. Eligibility criteria

Important

After examination by the Evaluation Committee, the application dossiers that do not meet the eligibility criteria will in no case be eligible for funding.

1) The project has to **enter into the scope** of the call for proposals as described §2.

2) The project proposal must not be considered by the Evaluation Committee as infringing an intellectual property right characterizing a counterfeiting within the meaning of intellectual property.

3.3. Evaluation criteria

Important

The dossiers that meet the acceptability and eligibility criteria will be evaluated according to the following criteria.

External experts and Evaluation Committee members are requested to review the proposals according to the evaluation criteria below. To help them in their evaluation, appreciation elements are suggested for each criterion while being neither restrictive nor mandatory.

1) Relevance of the research project proposal with respect to the orientations of the call for proposals:
2) Innovative nature of the proposal
    - Proposal will be based on original works in terms of scientific, technological and medical innovations,
    - Proposal will aim to extend existing works in order to product innovations in the treatment of concerned pathology(ies).
3) Preliminary data
    - Published preliminary data especially by the coordinating team should allow to support the proof of concept on which is based the project (cf. annex preliminary data planned 3.1).
4) Scientific and technical quality
    - Positioning with respect to the state of the art or innovation,
    - Structuring the project, precision in defining the final results (deliverable), identification of milestones,
    - Step risks identification and proposal propositions to remove technical barriers and alternative possible means
    - Methodology quality and detailed description of the hypothesis and tools used for the preclinical and clinical trials (annex of methodology)
5) Overall impact of the project
    - Prospects for medical and/or public health applications,
    - Prospects for industrial and/or technological use and economic and commercial potential, business plan, integration into the industrial activity, reliability of the expected valorisation,
    - Whenever relevant, addressing the issue of the environmental impact,
6) Quality of the consortium

- Scientific quality, experience in conducting multi-partner project and involvement of the scientific and technological project leader.
- Level of scientific excellence or expertise and relevance of the choice of the partner teams,
- Coordination and governance plan quality (project management in its functional, technical, organizational, time-based and financial aspects),
- Active role of the partner enterprise(s),
- Implementation and definition of the role of a scientific advisory board (SAB),
- Involvement of ongoing projects from PIA (Labex, Equipex, Cohorts, Demonstrators, Infrastructures, nanobiotechnologies and bioinformatics program, Technologic Research Institutes...).

7) Matching resources to project / Project feasibility

- Achievable timelines,
- Adjustment of the resources implemented to project management,
- Adjustment and justification for the amount of funding requested,
- Adjustment of coordination costs,
- Justification of costs of permanent staff,
- Justification of costs of non-permanent staff (internship, thesis, post-docs),
- Assessment of the amount of investments and equipment purchase,
- Assessment of other expenditure items (missions, subcontracting, consumables…).

3.4. Important recommendations

It is strongly recommended to register at the earliest opportunity and to regularly consult the program website, indicated on the first page of this document. It will give updated information and links to the reference documents and to the submission website.

The following recommendations provide advice to prepare projects proposals in the frame of this call for proposals.

As the Evaluation Committee may have to judge the relevance of any deviation from the recommendations, it is essential to explain the reasons for any such deviation.

Involvement of enterprises

Within the frame of this call for proposals, applicants are invited to present projects in which enterprises are strongly involved. In this spirit, their financial involvement should be significant, whilst complying with the community framework relating to support for research, development and innovation. To be accepted, the cost requested by the partnered enterprises should reveal an incentive effect (cf. definition §6.4).

The partnered enterprises (especially small and medium-sized enterprises) must pay attention to their genuine capacities for financing their inputs to the proposal. Overly optimistic or unrealistic forecasts may penalise the whole proposal. ANR may subsequently decide not to fund the proposal or to stop it.

Foreign partners

The French subsidiaries of foreign enterprises are eligible to the funding provided their R&D activities are carried out in France. Foreign teams (public or private) may participate (without any funding) in projects. Nevertheless, there should be necessarily a real and significant return for the health sector in France, and particularly for the enterprises. Furthermore, the foreign partner must provide its own funding.

Innovation and its protection

Special attention will be drawn to the originality and the innovative character of the project. The intellectual property issues will need to be precisely and clearly described. It is essential to set out the chosen protection strategy and to align it with the product, the technology or the service.

Regulatory and ethical aspects

Special attention will be drawn to the quality of the analysis of regulatory and ethical issues associated with the project, as well as to the acceptability of the proposed research by the enterprise. The dossier must include a description of the regulatory framework, the constraints and obligations related to the project, the potential product and the targeted deliverables. Whenever appropriate, the steps to be performed, the authorizations to be obtained, the enforceable framework and the applicable good practices (of laboratory …) will have to be detailed. Moreover, each step and authorization will have to be scheduled in the corresponding work package and in the global planning of the project.

4. General provisions for the funding

4.1. Funding

The project will be funded by an allocation from the State to ANR within the frame of the Investments for the Future program.

Funding methods

The funding awarded will be provided in the form of a non-reimbursable grant, according to the provisions of the "regulations on the funding modalities within the frame of calls for proposals in the hospital-university research", available on the website of the calls for proposals.

The grants will be paid to the Coordinator Institution (cf. definition §6.1). These grants may be subject to a transfer to the Partner Institutions, in accordance with the conditions provided in the regulations on the funding modalities within the frame of calls for proposals in the hospital-university research, available on the website of the calls for proposals.

The payment of a pre-funding instalment immediately following the announcement of the results will allow rapid initiation of the project. The pre-funding agreement will be in force until the signature of the final funding agreement with the Coordinator Institution, comprising all the supporting documents, but without exceeding 12 months. The pre-funding amount will not exceed 10% of the total amount granted to the project, by decision of the Prime Minister.

Conditions for funding temporary staff

Temporary staff (trainees, doctoral students, post-docs, fixed-term contracts, interim workers, etc.) may be assigned to the project. Except in special cases, throughout the project, the workload (person/month) funded by ANR should not exceed 50% of the total workload (person/month) committed to the project.

4.2. Consortium agreements

Under the auspices of the coordinator institution, the partner institutions will have to conclude an agreement that specifies:

- The distribution of tasks, human and financial resources and, deliverables,
- The sharing of intellectual property rights on the results obtained within the frame of the project,
- The publication scheme / dissemination of results,
- The valorisation of the project results.

This agreement will allow the existence of any indirect financial support included in the calculation of the maximum financial support rate approved by the community framework for research, development and innovation (hereinafter referred to as "the framework") to be determined.

The absence of indirect financial support is assumed if at least one of the following conditions is satisfied:

- The recipient subject to the Framework bears the total cost of the project,
- If the results cannot be protected by an intellectual property right, the recipient research organization is allowed to widely disseminate its results,
- If the results can be protected by an intellectual property right, the recipient research organization retains ownership of that right,
- The recipient subject to the Framework who uses a result developed by a recipient research organization pays to this organization fees equivalent to the market conditions.

The project coordinator institution will forward a copy of this agreement to ANR, along with a certificate signed by the partner institutions and confirming compliance with the Framework regulations, and with the agreement(s) defining the conditions of project execution and funding. **Forwarding must be carried out within 12 months from the date of signature of the prefunding convention**.

This certificate should therefore confirm either that the agreement satisfies one of the above listed conditions, or that all intellectual property rights on the results and rights of access to the results are allocated to the different partner institutions and properly reflect their respective interests, level of involvement in the works and financial and other contributions to the project.

4.3. Other provisions

The funding of a project does not dispense the involved partners from meeting their obligations regarding the regulation, the ethical rules and the code of ethics applicable in their

activity area.

On behalf of all the partner institutions, the project's coordinator institution commits to keep ANR informed of any change modifying the content, the partnership and the completion timeline of the project, from its submission to the publication of the list of selected projects.

5. Conditions of submission

5.1. Content of the submission dossier

The submission dossier shall contain all the necessary elements for the scientific and technical evaluation of the project. It must be complete at the closing of the call for proposals, whose date and time are indicated on page 2.

Important

No additional element will be accepted after the closing of the call for proposals whose date and time are indicated on page 2.

The complete dossier will be filed via the submission website whose address and dates are indicated on page 2. To have access to this service, an account must first be opened (username and password). It is recommended to register as soon as possible to get these elements.

The complete submission dossier should include the following documents fully filled in:

- **The "administrative and financial document" and the "letters of undertaking of the Coordinator Institution and of the Partner Institutions" available online on the submission website;**

- **The "scientific document" which describes the scientific, technical and clinical aspects of the project, as well as its educational and valorisation objectives.** The scientific document has to be supplemented by three annexes: one devoted exclusively to the description of the preclinical and clinical trial methodology proposed in the project and necessary for the scientific evaluation, one dealing with preliminary data and one about intellectual property.

The different parts of the submission dossier (administrative and financial, templates of letters of undertaking and of the scientific document) will be available on the web page of the present call for proposals (address on the 1st page).

Important: the projects being evaluated by an international committee, it is recommended to provide an English description of the project. Should it be written in French, the evaluation committee could request an English version within a deadline consistent with the evaluation process.

5.2. Submission procedure

The elements of the submission dossier must be transmitted by the scientific and technical project leader:

1) IN ELECTRONIC FORMAT (administrative and financial document and the unique scientific document plus three annexes), imperatively:

- Before the closing dates indicated on page 2 of the present call for proposals,
- Via the submission website, according to the recommendations in §5.1.

Prior registration on the submission website is mandatory in order to submit a project.

Only the electronic version of the submission documents, posted on the submission website at the closing of the call for proposals, will be transmitted to the experts and evaluation committee members for evaluation.

2) SIGNED VERSION IN SCANNED FORM ("administrative and financial document" and the letters of undertaking), imperatively:

- Signed by the scientific and technical project leader, by the legal representative of the coordinator institution and by all the partner institutions,
- Submitted online on the submission website before the closing date indicated on page 2 of the present call for proposals, with submission date and time as proof of submission.

NB: The signed version is used to certify that the partner institutions of the project agree on the project submission.

AN ACKNOWLEDGMENT OF RECEIPT in electronic format will be sent to the Scientific and Technical Manager of the project once uploading of the submission dossier and of the signed documents is validated.

5.3. Advice for submission

It is strongly recommended:

- To open an account on the submission website at the earliest opportunity,
- Not to wait for the limit date of sending the projects to enter the data online and to upload the requested documents (be careful: compliance with the submission time limit is mandatory),
- to verify that the documents submitted in the dedicated spaces under the headings "submission documents" and "signed documents" are complete and correspond to the expected elements, in particular a letter of undertaking is mandatory for each partner institution. The submission file and the deposit of the signed documents will only be validated by the scientific and technical project leader if all the documents have been downloaded.

- To regularly consult the call for proposals website (at the address indicated on 1st page) which updates information on its progress,
- If necessary, to contact the correspondents via electronic mail at the addresses indicated on page 2 of the present documents.

6. Appendix

6.1. Definitions relating to the project organization

Coordinator institution: endowed with legal personality, it is the preferred contact of ANR as regards administrative matters. It is in charge of implementing and formalizing the collaboration between the partner institutions, of producing the project's deliverables, of holding follow-up meetings and of communicating the results. To this end, it relies on a scientific and technical project leader. It signs the grant agreement with ANR and receives the funding attributed to the proposal.

Scientific and technical project leader: S/He ensures the technical, clinical and scientific coordination of the project on behalf of the coordinator institution. S/He is the physical person, technically and scientifically in charge of the coordination structure. S/He is the preferred contact person of ANR.

Partner: research unit of a research organization or of an enterprise member of the project. Each of the partner units appoints a technical and scientific correspondent, the preferred contact of the technical and scientific project leader.

Partner institution: research organization or healthcare institution responsible for a partner unit, or a research organization or a healthcare institution allocating resources to the partner unit or enterprise this partner unit depends on. If appropriate and in accordance with a subcontract, it benefits a funding share paid by ANR to the Coordinator institution for the realisation of a task or mission in the framework of the project.

6.2. Definitions relating to the structures

Enterprise: designates small, medium-size (SME) and major enterprises. The definition of small and medium-size enterprises (SME) is the one of the regulation (EC) n° 70/2001 of the European Commission of January 12, 2001, included in the recommendation 2003/361/CE of the European Commission of May 6, 2003 and regarding the definition of medium, small and micro enterprises, and any other substitute communitarian text. Within the meaning of Community law, an enterprise is any entity taking up economic activity regardless of its legal form. Economic activity means any activity offering goods and services on a given market.

Research organization: this term should be understood under the meaning of the 2.2 d) Framework. This is an entity, such as a university or a research institution, whatever its legal status (private or public law institution) or its type of financing. Its main purpose is to conduct activities of fundamental research, industrial research or experimental development, and to disseminate their results through education, publication or transfer of technology. The benefits

are totally re-invested in those activities, in the dissemination of their results or in education. The enterprises having a potential interest in such entities, for example as a shareholder or a member, do not benefit from any preferred access to their research capacities or to the results produced.

Territorial collectivities: **endowed with legal personalities according to public law and distinct from the state**, benefiting as such from patrimonial and legal autonomy. The equivalent term in normal English usage would be "regional or local authorities". For example, the following are designated as territorial collectivities: the municipalities; the departments, including the 5 French overseas departments (Dom); the regions, including the 5 overseas regions; the collectivities with a special status; the overseas collectivities (Com).

Health care institutions: structures ensuring the diagnosis, the monitoring and the treatment of sick persons, injured persons and pregnant women. They provide inpatient, outpatient and home-based care. They are committed in the coordination of health care in relation with the health professionals who practice in private office and medico-social institutions and services. They contribute to the implementation of the public health policy and of vigilance. They conduct a reflection on the ethics associated with the reception and management of patients (Article L6111-1 et seq. of the French Public Health Code).

6.3. DEFINITIONS RELATING TO THE DIFFERENT CATEGORIES OF RESEARCH

These definitions are given in the community framework for State aid for research, development and innovation[6].

Fundamental research: means experimental or theoretical work undertaken primarily to acquire new knowledge of the underlying foundations of phenomena and observable facts, without any direct commercial application or use in view.

Industrial research: means the planned research or critical investigations aimed at the acquisition of new knowledge and skills for developing new products, processes or services for bringing about a significant improvement in existing products, processes or services. It comprises the creation of components parts of complex systems, notably for generic technology validation, excluded the prototypes concerned by the definition of experimental development described below.

Experimental development: means acquiring, combining, shaping and using existing scientific, technological, business and other relevant knowledge and skills to produce designs, devices or drawings with the aim of developing new or improved products, processes or services. This may also include, for example, activities aiming at the conceptual definition, planning and documentation of new products, processes and services as well as the recording of related information. These activities may also comprise prototypes, plans and other

---

[6] Cf. JOUE 30/12/2006 C323/9-10
http://www.agence-nationale-recherche.fr/documents/uploaded/2007/encadrement.pdf

documents, provided they are not aimed to a commercial use.

The development of a commercially usable prototype or pilot is experimental development when the prototype is necessarily the final commercial product and when it is too expensive to produce for it to be used only for demonstration and validation purposes. In case of future commercial use of demonstration product or of pilot projects, any benefit from such a use would be deducted from the eligible costs.

The experimental production and testing of products, processes and services can also benefit from an aid provided they cannot be used or transformed for a use for industrial or commercial applications.

Experimental development does not include routine or periodic changes made to existing products, production lines, manufacturing processes, services and other operations in progress, even if those changes may represent improvements.

6.4. Other definitions

Incentive effect: according to the community provisions, means that the financing support should bring a change in the recipient's behaviour, leading him to strengthen his R&D activities: it should result in an increase in scale, impact, budget or rhythm of the R&D activities. The analysis of the incentive effect will be based on a comparison of the situation with and without any grant, from answers to questions sent to the enterprise. Several markers may be used: total cost of the project, R&D staff dedicated to the project, scale of the project, risk level, increase in the risk level of the activities, increase in R&D expenses within the enterprise
