# Supplementary material for: Effects of a personalized or generic three-dimensional tumoral kidney model on patient experience and caregiver-patient interactions, before and after partial nephrectomy, a randomized trial (Rein 3D Print Personalize—UroCCR 114)
Source: PLoS One. 2025 Aug 18;20(8):e0323515. doi: 10.1371/journal.pone.0323515 (PMC12360608; doi:10.1371/journal.pone.0323515)
Supplement: S5 File — (PDF) [file pone.0323515.s005.pdf]

HLS-EU16 version Française (Version 2020)

Indiquez, sur une échelle de très facile à très difficile, dans quelle mesure il est facile pour vous de...

	très facile	facile	difficile	très difficile
... trouver des informations sur les traitements des maladies qui vous concernent ?	❑	❑	❑	❑
… savoir où obtenir l'aide d'un professionnel quand vous êtes malade ? (Par ex. médecin, infirmier, pharmacien ou psychologue)	❑	❑	❑	❑
… comprendre ce qu'un médecin vous dit ?	❑	❑	❑	❑
… comprendre les consignes de votre médecin ou pharmacien sur la manière de prendre vos médicaments ?	❑	❑	❑	❑
… savoir quand il serait utile d'avoir l'avis d'un autre médecin ?	❑	❑	❑	❑
… utiliser les informations que le médecin vous donne pour prendre des décisions concernant votre maladie ?	❑	❑	❑	❑
… suivre les consignes de votre médecin ou pharmacien ?	❑	❑	❑	❑
… trouver des informations sur comment faire en cas de problèmes psychologiques ? (Par ex. stress, dépression ou anxiété)	❑	❑	❑	❑
… comprendre les mises en gardes concernant l'impact sur la santé de certains comportements comme fumer, ne pas faire assez d'exercice et boire trop ?	❑	❑	❑	❑

Tournez la page s'il vous plaît…

Indiquez, sur une échelle de très facile à très difficile, dans quelle mesure il est facile pour vous de...

	très facile	facile	difficile	très difficile
… comprendre les informations sur les dépistages et examens recommandés ? (Par ex. dépistage du cancer colorectal, test de glycémie)	❑	❑	❑	❑
… évaluer la fiabilité des informations disponibles dans les médias sur ce qui est dangereux pour la santé ? (Par ex. journaux, télévision ou internet)	❑	❑	❑	❑
… savoir comment vous protéger des maladies à partir des informations disponibles dans les médias ? (Par ex. journaux, télévision ou internet)	❑	❑	❑	❑
… vous renseigner sur les activités bénéfiques pour votre santé et votre bien être ? (Par ex. relaxation, exercice physique, yoga)	❑	❑	❑	❑
… comprendre les conseils de votre famille ou de vos amis en matière de santé ?	❑	❑	❑	❑
… comprendre les informations disponibles dans les médias pour être en meilleure santé ?	❑	❑	❑	❑
… identifier quels sont les comportements de votre vie de tous les jours qui ont un impact sur votre santé ?	❑	❑	❑	❑

Fin du questionnaire.
