# Supplementary material for: Effects of a personalized or generic three-dimensional tumoral kidney model on patient experience and caregiver-patient interactions, before and after partial nephrectomy, a randomized trial (Rein 3D Print Personalize—UroCCR 114)
Source: PLoS One. 2025 Aug 18;20(8):e0323515. doi: 10.1371/journal.pone.0323515 (PMC12360608; doi:10.1371/journal.pone.0323515)
Supplement: S6 File — (PDF) [file pone.0323515.s006.pdf]

1/ What I know about the kidney, generally speaking	1/ Ce que je sais sur le rein, de manière générale	False / Faux	True / Vrai	Don't know / je ne sais pas
1.1- The kidney is a paired organ	1.1- Le rein est un organe pair			
1.2- The kidney function is to "clean" my blood	1.2- La fonction du rein est d'épurer le sang			
1.3- The kidney produces urines	1.3- Le rein produit l'urine			
1.4- The urine is collected in cavities inside of the kidney, called the collecting system	1.4- L'urine est recueillie dans des cavités situées à l'intérieur du rein, appelées système collecteur.			
1.5- The kidney is a highly vascularized organ; a lot of blood lows through the renal vessels	1.5- Le rein est un organe très vascularisé, avec un débit sanguin important			
1.6- The renal vessels can be described as an artery and a vein	1.6- Les vaisseaux du rein comprennent l'artère et la veine rénales			
1.7- To work properly the kidney needs blood coming through the renal artery	1.7- Pour fonctionner correctement, le rein a besoin de sang qui arrive par l'artère rénale			
1.8- When the kidneys are not working properly this leads to renal insufficiency	1.8- Lorsque les reins ne fonctionnent pas correctement, cela conduit à l'insuffisance rénale			
**2/ What I know about my disease:**	**2/ Ce que je sais de ma maladie :**	**False / Faux**	**True / Vrai**	**Don't know / je ne sais pas**
2.1- My kidney is bearing a tumor	2.1- Mon rein est porteur d'une tumeur			
2.2- The tumor is located in the mid part of my kidney	2.2- La tumeur est située dans la partie médiane de mon rein			
2.3- The tumor is in close contact with the kidney vessels	2.3- La tumeur est en contact étroit avec les vaisseaux du rein			
2.4- The tumor is in close contact with the collecting system	2.4- La tumeur est en contact étroit avec le système collecteur			
**3/ What I understand about my planned surgery**	**3/ Ce que je comprends de l'intervention chirurgicale prévue**	**False / Faux**	**True / Vrai**	**Don't know / je ne sais pas**
3.1- My surgeon will try to remove the tumor only	3.1- Mon chirurgien essaiera d'enlever uniquement la tumeur			
3.2- My surgeon will remove the entire kidney	3.2- Mon chirurgien va enlever tout le rein			
In case of tumor only removal my surgeon will have to cut the kidney itself to separate the tumor from surrounding healthy tissue. This may lead to: 3.3- bleeding with a risk of hemorrhage	Dans le cas d'une ablation de la tumeur uniquement, mon chirurgien devra couper le rein lui-même pour séparer la tumeur des tissus sains qui l'entourent. Cela peut entraîner 3.3- des saignements avec un risque d'hémorragie			
3.4- opening the collecting system with a risk of urine leakage	3.4- l'ouverture du système collecteur avec un risque de fuite d'urine			
3.5- To reduce the risk of hemorrhage at the time of tumor removal my surgeon may need to clamp (=interrupt blood-low) the renal artery	3.5- Pour réduire le risque d'hémorragie au moment de l'ablation de la tumeur, mon chirurgien peut être amené à clamper (= interrompre le flux sanguin) l'artère rénale.			
3.6- Prolonged renal artery clamping is known to alter renal function so my	3.6- Le clampage prolongé de l'artère rénale est connu pour altérer la fonction			

surgeon will have to speed up the procedure to limit the length of clamping	rénale, mon chirurgien devra donc accélérer la procédure pour limiter la durée du clampage			
3.7- In case of tumor only removal, the benefit is preservation of healthy kidney tissue	3.7- En cas d'ablation de la tumeur uniquement, l'avantage est la préservation du tissu rénal sain.			
3.8- Preserving healthy tissue from my tumor bearing kidney decreases the risk of renal insuficiency	3.8- Préserver le tissu sain de mon rein porteur de tumeur<br>diminue le risque d'insuffisance rénale			
