# Supplementary material for: Effects of a personalized or generic three-dimensional tumoral kidney model on patient experience and caregiver-patient interactions, before and after partial nephrectomy, a randomized trial (Rein 3D Print Personalize—UroCCR 114)
Source: PLoS One. 2025 Aug 18;20(8):e0323515. doi: 10.1371/journal.pone.0323515 (PMC12360608; doi:10.1371/journal.pone.0323515)
Supplement: S7 File — (PDF) [file pone.0323515.s007.pdf]

S'il vous plaît, notez de 1 à 10 (1 = pas d'aide du tout -> 10 = d'une grande aide) dans quelle mesure la présentation du modèle de Rein imprimé en 3D vous a aidé à :

1. Apprendre « des notions » sur le rein lui-même :

1 2 3 4 5 6 7 8 9 10

Pas d'aide du tout — D'une grande aide

2. Comprendre votre maladie :

1 2 3 4 5 6 7 8 9 10

Pas d'aide du tout — D'une grande aide

3. Comprendre la chirurgie prévue :

1 2 3 4 5 6 7 8 9 10

Pas d'aide du tout — D'une grande aide

4. Comprendre le risque de complications liées à l'intervention chirurgicale prévue

1 2 3 4 5 6 7 8 9 10

Pas d'aide du tout — D'une grande aide
