# Supplementary material for: Effects of a personalized or generic three-dimensional tumoral kidney model on patient experience and caregiver-patient interactions, before and after partial nephrectomy, a randomized trial (Rein 3D Print Personalize—UroCCR 114)
Source: PLoS One. 2025 Aug 18;20(8):e0323515. doi: 10.1371/journal.pone.0323515 (PMC12360608; doi:10.1371/journal.pone.0323515)
Supplement: S8 File — (PDF) [file pone.0323515.s008.pdf]

Projet UroCCR et UroCCR Chain
Version n°5.0 du 13/03/2023

NOTE D'INFORMATION POUR LA CONSTITUTION DE BASES DE DONNEES A VISEE DE RECHERCHE ET D'EVALUATION DES SOINS AVEC COLLECTION VIRTUELLE D'ECHANTILLONS BIOLOGIQUES SUR LE CANCER DU REIN : UROCCR ET UROCCR-CHAIN

Gestionnaire du Réseau UroCCR : **CHU de Bordeaux**
Coordonnateur : **Pr Jean-Christophe BERNHARD**

Madame, Monsieur,

Votre médecin spécialiste et le service auquel il appartient participent à un projet multicentrique national soutenu par l'Institut National du Cancer (INCa). Ce projet, intitulé UroCCR, consiste en la création et le déploiement d'une base de données dédiée à la recherche multidisciplinaire sur le cancer du rein, associée à une banque de ressources biologiques virtuelle.

Lors de votre prise en charge médicale, des prélèvements biologiques (sang, urines, tissus…) peuvent être effectués afin de permettre un diagnostic, la surveillance de votre maladie et son traitement. Cette prise en charge peut également nécessiter le recueil de données médicales. A l'issue de leur utilisation à des fins médicales, ces ressources biologiques et ces données sont susceptibles de présenter un intérêt pour la recherche scientifique en lien avec votre pathologie, réalisée sous contrôle de la pertinence scientifique par un comité d'experts.

Les médecins des différentes spécialités impliquées (Chirurgie Urologique, Oncologie médicale, Imagerie médicale et anatomo-pathologie notamment), pourront ainsi collecter l'ensemble des renseignements cliniques et biologiques en rapport avec votre maladie et sa prise en charge.

Dans le cadre d'UroCCR, le Gestionnaire pourra communiquer des informations personnelles aux agences réglementaires ou à ses partenaires de recherche. Ces personnes, sociétés et agences peuvent être situées en France, dans d'autres pays de l'Espace Economique Européen (EEE), aux États-Unis et dans d'autres pays à l'extérieur de l'EEE. Il est possible que certains pays hors de l'EEE n'offrent pas le même niveau de protection de la vie privée qu'en France. Dans ce cas, le Gestionnaire maintiendra toutefois la confidentialité de toutes les informations personnelles qu'il échangera dans les limites de la loi. Le Gestionnaire adoptera les mesures contractuelles appropriées relatives à la protection et au transfert des données, pour s'assurer que les destinataires pertinents en dehors de l'EEE fournissent un niveau adéquat de protection concernant vos informations personnelles et conformément à la loi.

En parallèle, des données du Système National des Données de Santé (« SNDS », c'est-à-dire les données de l'Assurance Maladie) permet d'obtenir une meilleure compréhension de vos pathologies dans la mesure où elles permettent d'avoir un suivi plus complet de chaque patient. Dans ce cadre, le CHU de Bordeaux a également décidé de mettre en œuvre, en collaboration avec la société Clinityx, un second projet « UroCCR-Chain », afin d'intégrer ces données pour permettre de réaliser des analyses.

Il est important que vous lisiez attentivement ces pages qui vous apporteront des informations sur l'utilité d'un tel recueil de données ainsi que sur ses modalités pratiques. N'hésitez pas à nous poser toutes les questions que vous jugerez utiles.

Pour rappel, UroCCR et UroCCR-Chain sont deux projets, qui consistent en la mise en place d'une Base de Données pour le premier et d'un Entrepôt de données de santé (EDS) pour le second, dont l'objectif commun est de mettre en place de nombreuses études ultérieurement.

Les informations relatives aux différents projets menés seront disponibles sur www.uroccr.fr.

Vous pouvez à tout moment vous opposer à ce que vos échantillons et données soient utilisés pour tout ou partie des projets référencés sur www.uroccr.fr sans avoir de justification à fournir.

	**UroCCR**	**UroCCR-Chain**
Personnes en charge du projet (Responsable de traitement ou coresponsables de traitement)	Le CHU de Bordeaux dont la Direction Générale est située 12 rue Dubernat 33400 Talence France.	Le CHU de Bordeaux et Clinityx (société spécialisée dans l'analyse de données de l'Assurance Maladie et la mise en place d'entrepôts de données de santé) dont le siège social est situé 137 rue d'Aguesseau 92160 Boulogne Billancourt
Finalité (Raison de la mise en place des projets)	Les objectifs d'UroCCR sont :<br>- permettre la réalisation d'études épidémiologiques, observationnelles ou translationnelles, à échelon national ou international, dans le domaine du cancer du rein, liées à :<br>- l'épidémiologie descriptive du cancer du rein ;<br>- la pharmaco-épidémiologie et l'observation des traitements ;<br>- la qualité de vie et les conséquences personnelles, familiales, profession-nelles et sociales du cancer du rein ;<br>- la recherche de biomarqueurs diagnostiques et pronostiques ;<br>- la recherche de marqueurs prédicteurs de l'évolution de la maladie ;<br>- l'évaluation des pratiques de soins et des techniques de traitement.	L'entrepôt de données de santé « UroCCR-Chain » a les mêmes finalités qu'UroCCR avec des données enrichies de l'assurance maladie afin d'obtenir un suivi complet de la pathologie et de disposer d'information médico-économiques (coûts de prise en charge).
Base légale (raison juridique de la mise en place de ce projet)	Le recueil/traitement de ces données sera fait à des fins de recherche scientifique et trouve son fondement dans l'intérêt public des missions du CHU de Bordeaux.	Le traitement mis en œuvre par les coresponsables de traitement (CHU de Bordeaux et Clinityx) est nécessaire aux fins des intérêts légitimes qu'ils poursuivent.
Destinataires des données à caractère personnel	Etablissements participants au réseau UroCCR et autres partenaires de recherche dans le cadre des projets ancillaires.	Le CHU de Bordeaux et Clinityx uniquement.
Informations concernées (Données personnelles)	Données de santé vous concernant, données relatives au phénotype africain et données génétiques issues de votre dossier médical.	Les mêmes données de santé que pour UroCCR enrichies des données de l'Assurance Maladie (consommation de soins, consul-tations, traitements, hospitalisations, statut vital…).
	Afin d'assurer la confidentialité de vos informations à caractère personnel, ni votre nom ni aucune autre information qui permettraient de vous identifier directement ne seront saisis dans un dossier ou sur un échantillon que le médecin du projet fournira au Gestionnaire ou aux représentants autorisés du Gestionnaire. Vous serez uniquement identifié(e) par un code et vos initiales. Le code est utilisé pour que le médecin du projet puisse vous identifier si nécessaire. Ces données seront enregistrées sur des serveurs informatiques sécurisés.	
Durée de conservation	15 ans après le dernier suivi du patient.	10 ans à compter de la mise en place du projet.

<table>
<tr><td>Coordonnées du Délégué à la protection des données personnelles</td><td colspan="2">Le Délégué à la protection des données personnelles du CHU de Bordeaux : mesdonneespersonnelles@chu-bordeaux.fr</td></tr>
<tr><td rowspan="2">Vos droits (loi relative à l'informatique, aux fichiers et aux libertés n° 78-17 du 6 janvier 1978 relative à l'informatique, aux fichiers et aux libertés modifiée par la loi n° 2018-493 du 20 juin 2018 relative à la protection des données personnelles ; Règlement général sur la protection des données UE 2016/679)</td><td>Vous disposez des droits suivants sur vos données collectées et générées dans le cadre de votre participation :
- Droit d'accès aux données,
- Droit de rectification des données erronées,
- Droit d'effacement des données en cas de traitement illicite,
- Droit de portabilité vous permettant d'obtenir les données que vous avez-vous-même fournies,
- Droit de limitation du traitement des données notamment si celui-ci venait à être remis en cause.</td><td>Vous disposez des droits suivants sur vos données collectées et générées dans le cadre de votre participation :
- Droit d'accès aux données,
- Droit de rectification des données erronées,
- Droit d'effacement des données en cas de traitement illicite,
- Droit de limitation du traitement des données notamment si celui-ci venait à être remis en cause.</td></tr>
<tr><td colspan="2">Vous disposez également d'un droit d'opposition au traitement de vos données. Dès lors, aucune nouvelle donnée à caractère personnel vous concernant ne sera collectée. L'exercice de ce droit empêche tout traitement ultérieur des données vous concernant et vous ne pourrez plus poursuivre votre participation à cette recherche. Toutefois, toute information déjà collectée au préalable sera conservée et continuera d'être utilisée dans le cadre de ces recherches.

Vous pouvez exercer ces droits en le demandant par écrit auprès du médecin qui vous suit dans le cadre de ces recherches qui transmettra la demande au CHU de Bordeaux. Le CHU de Bordeaux répondra à vos demandes conformément à ses obligations légales et réglementaires.

Vous pouvez également accéder directement ou par l'intermédiaire du médecin de votre choix à l'ensemble de vos données médicales en application des dispositions de l'article L1111-7 du code de la santé publique. Ce droit s'exerce auprès du médecin qui vous suit dans le cadre de ses recherches et qui connaît votre identité.</td></tr>
</table>

En cas d'insatisfaction, vous pouvez déposer une réclamation auprès de l'autorité de surveillance de la protection des données, la Commission Nationale de l'Informatique et des Libertés (CNIL) à l'adresse : https://www.cnil.fr/fr/webform/nous-contacter ou https://www.cnil.fr/fr/plaintes/.

Les résultats des projets menés sur UroCCR et UroCCR Chain pourront donner lieu à des innovations brevetées ou protégées par le droit de la propriété intellectuelle, et à des publications scientifiques.

Lorsque ces projets seront terminés, les résultats globaux seront accessibles sur le site www.uroccr.fr ou par votre médecin dès que ceux-ci seront disponibles et si vous le souhaitez.

Projet UroCCR et UroCCR Chain
Version n°5.0 du 13/03/2023

NOTE D'INFORMATION POUR LA CONSTITUTION DE BASES DE DONNEES A VISEE DE RECHERCHE ET D'EVALUATION DES SOINS AVEC COLLECTION VIRTUELLE D'ECHANTILLONS BIOLOGIQUES SUR LE CANCER DU REIN : **UROCCR ET UROCCR-CHAIN**

En remplissant ce document vous certifiez avoir pris connaissance de la note d'information jointe et avoir pu poser toutes les questions utiles à votre médecin. Merci de nous faire part de votre décision en complétant et en signant le formulaire joint ci-dessous.

Identification

Je soussigné(e)
Nom de famille (patronymique) : ....

Prénom : ....

Né(e) le : |_|_| (jj) |_|_| (mm) |_|_|_|_| (aaaa) Ville de naissance : ....

Pour les majeurs sous tutelle *(les majeurs sous curatelle ou sous sauvegarde de justice ne peuvent participer à ces activités)****, le représentant légal doit compléter les mentions ci-dessous*** :

Le représentant légal : ❑ Mme ❑ M.

Nom de famille : ....

Prénom : .... Né(e) le : |_|_| (jj) |_|_| (mm) |_|_|_|_| (aaaa)

Merci d'indiquer votre décision en cochant les cases correspondant à votre choix

Compte tenu de ces informations :

	**J'accepte :**	**Je refuse :**
Les échantillons biologiques et les informations médicales disponibles au décours de ma prise en charge peuvent être collectés, conservés et utilisés par mon établissement de soins participant à UroCCR ou transférés à l'usage d'autres équipes scientifiques dans le cadre du Projet UroCCR, jusqu'à leur épuisement (utilisation totale). La présentation des projets ancillaires est accessible sur le site www.uroccr.fr. L'affichage des projets de recherche sur cette page Internet vaut information, sans lettre d'information individuelle complémentaire.	❒	❒
Ces recherches non interventionnelles pourront comprendre l'examen de mes caractéristiques génétiques sans finalité identifiante. Elles ne pourront pas modifier ma prise en charge médicale et la manière dont me sont dispensés les soins.	❒	❒
J'accepte de participer à UroCCR-Chain. La présentation des projets ancillaires est accessible sur le site www.uroccr.fr.	❒	❒

Ce formulaire, ainsi que toutes les informations personnelles me concernant resteront strictement confidentiels, sont et resteront couverts par le secret professionnel et médical, ainsi que par le respect dû à ma vie privée. En vertu de la loi « Informatique et Libertés » du 6 janvier 1978 modifiée (notamment par la loi n° 2018-493 du 20 juin 2018 relative à la protection des données personnelles) et au règlement général sur la protection des données (règlement UE 2016/679), je dispose d'un droit d'accès, de rectification, de suppression, de limitation du traitement, à la portabilité des données (non applicable pour UroCCR-Chain), d'opposition et de retrait.
J'ai pris connaissance que :
- le projet UroCCR a reçu l'avis favorable du Comité Consultatif sur le Traitement de l'Information en matière de Recherche dans le domaine de la Santé (CCTIRS) le 24/10/2012 et l'autorisation de la Commission nationale de l'informatique et des libertés (CNIL) le 12/04/2013 (Décision DR-2013-206) et le 15/12/2016 (Décision DR-2016-485).
- l'entrepôt de données de santé UroCCR-Chain a reçu l'autorisation de la CNIL dans sa délibération n°2022-091 du 08/09/2022

Je suis libre de revenir sur ma décision en le signifiant par e-mail via www.uroccr.fr ou par téléphone au 05 57 82 23 94

Fait à : ....

Le : |_|_| / |_|_| / |_|_|_|_|

Signature du Patient

Fait à : ....
Nom du médecin: ....

Le : |_|_| / |_|_| / |_|_|_|_|

Signature du Médecin

Fait à :..

Le : |_|_| / |_|_| / |_|_|_|_|

Signature du représentant du Patient
