# Supplementary material for: Effects of a personalized or generic three-dimensional tumoral kidney model on patient experience and caregiver-patient interactions, before and after partial nephrectomy, a randomized trial (Rein 3D Print Personalize—UroCCR 114)
Source: PLoS One. 2025 Aug 18;20(8):e0323515. doi: 10.1371/journal.pone.0323515 (PMC12360608; doi:10.1371/journal.pone.0323515)
Supplement: S9 File — (PDF) [file pone.0323515.s009.pdf]

Committee for the Protection of Individuals South-East IV

Opinion on an initial application

CPP

**Comitee name**	Committee for the Protection of Individuals South-East IV
**Adress**	Centre Léon Bérard - 28 rue Laennec 69373 LYON CEDEX 08 France
**Email**	cppse4@lyon.unicancer.fr
**Contact**	0478782761

Promotor

**Promotor**	CHU BORDEAUX
**Représentant légal (UE) :**	-
**Mandataire :**	-

File

**SI number**	24.00621.000226
**National number**	2024-A00129-38
**Internal reference**	CHUBX2023/78

**Legislation**	Jardé Law
**Qualification :**	Second Category
**Product**	Excluding health products (products not mentioned in article L.5311-11 of the French Public Health Code)

**Investigator**	Gaëlle MARGUE
**Title**	**Effects of a personalized or generic three-dimensional tumoral kidney model on patient experience and caregiver-patient interactions, before and after partial nephrectomy (Rein 3D Print Personalize UroCCR 114).**

This file was examined at a meeting on 12/03/2024 and a mandate was given to the Chairman of the PPC to issue an opinion on receipt of the applicant's responses to the latest requests.
the opinion on receipt of the applicant's responses to the latest requests. In view of the responses received the following opinion was issued. This notice runs from the change of status on the IS.

Considering that the ethical conditions have been met, in particular with regard to the elements of article L.1123-7 of the Public Health Code, the Committee's review leads it to conclude that the research may be carried out and to issue the following opinion :

Favourable opinion

This opinion is valid for two years. In accordance with article L.1123-11 of the French Public Health Code, the sponsor must declare the start of the research to the CPP. This declaration is made directly on SIRIPH2G.

If you have not been able to include your first research participant within this deadline, you may ask the CPP for an extension of this notice before it expires (article R.1123-26 of the Public Health Code).

Participants to the deliberation

College	Category	Name and Surname
First college	Qualification RIPH-Biostatistics or epidemiology	O. PEROL
		P. CONY-MAKHOUL
	RIPH-Other qualification	S. BÉNÉZECH
		S. METZGER
		C. ABDELKRIM
		A. BERTRAND REYNAUD
		M. MONTANGE
	Specialist in general medicine	G. WALLON
	Medical auxiliary	G. DUYCK
		S. BOUVET
Second college	Ethical competence	A. GELOT
		D. SALAKO
	Competence in the humanities or social action	V. BAUDRY
	Legal competence	E. CHAPOUTIER
		M-A. EUDELINE
	Association representative association	P. CHEMLI
		J. SASSARD

Letter requesting an opinion, dated and signed

Administrative folder	N° version	Date (dd/mm/yy)
Letter requesting an opinion, dated and signed		02/02/2024
Letter in response to request for additional information		14/02/2024
Cover letter		05/04/2024
Letter in response to deliberation A24-067		28/03/2024
Comparison table		28/03/2024
Request for advice form, dated and signed		14/02/2024
Additional document to the request for advice to the CPP		31/01/2024
**DOSSIER SUR LA RECHERCHE**	**N° version**	**Date (jj/mm/aaaa)**
Research protocol (signature page: sponsor and coordinating investigator)	1.1	29/03/2024
Summary of the protocol in French	1.0	23/01/2024
Information document(s) and consent form(s) :<br>Patients *<br>Professional *	 1.1<br>1.1	 29/03/2024<br>29/03/2024
Certificate of insurance: beH B1339CTLICNWL23-30		25/01/2024
Adequacy of human, material and technical resources and compatibility with safety of participants, unless the venue has been granted the authorisation referred to in article L.1121-13 of the public health code: CHU Bordeaux		31/01/2024
List of investigators (1 investigators including national coordinator / 1 participating centres)	1.0	23/01/2024
Dated and signed CV of the corresponding investigator (Dr Gaëlle MARGUE - CHU de BORDEAUX - BPC 2023)		29/01/2024
Other document(s):<br>Declaration of conformity		20/01/2017

*Document(s) sent in 'tracked changes' and final versions

The Chairman. Dr Amandine BERTRAND

signed on 24-04-2024

Amandine Bertrand

✓ Certified by yousign
