# Supplementary material for: Effects of a personalized or generic three-dimensional tumoral kidney model on patient experience and caregiver-patient interactions, before and after partial nephrectomy, a randomized trial (Rein 3D Print Personalize—UroCCR 114)
Source: PLoS One. 2025 Aug 18;20(8):e0323515. doi: 10.1371/journal.pone.0323515 (PMC12360608; doi:10.1371/journal.pone.0323515)
Supplement: S10 File — (PDF) [file pone.0323515.s010.pdf]

Effects of a personalized or generic three-dimensional tumoral kidney model on patient experience and caregiver-patient interactions, before and after partial nephrectomy (Rein 3D Print Personalize UroCCR 114)

Kidney 3D PRINT PERSONALIZE

UroCCR N° 114

Study Code : CHUBX 2023/78

Research Category: Category 2 - Minimal Risk and Constraint Research)

Version 2.0, 04/06/2024

ID-RCB NUMBER : 2024-A00129-38

This intervention research received funding from ANR-21-RHUS-0015

Sponsor:
Bordeaux University Hospital 12, rue Dubernat
33 400 Talence
France

Coordinating Investigator:
Dr Gaëlle MARGUE
Bordeaux University Hospital - Pellegrin Hospital
Urology Department Place Amélie Raba Léon
33076 Bordeaux Cedex
Tél. : 05 57 82 17 62
Courriel : gaelle.margue@chu-bordeaux.fr

Scientific Lead:
Pr. Jean-Christophe BERNHARD
Bordeaux University Hospital - Pellegrin Hospital
Urology Department Place Amélie Raba Léon
33076 Bordeaux Cedex
Place Amélie Raba-Léon
33076 Bordeaux Cedex
Tél. : 05 57 82 17 62
Courriel: jean-christophe.bernhard@chu-bordeaux.fr

Methodology Support Center :
Methodology and Data Management Center:
Pr. Laura RICHERT
Clinical and Epidemiological Research Methodology Support Unit of Bordeaux University Hospital Case 75, 146 rue Léo Saignat
33076 Bordeaux cedex
Courriel: laura.richert@chu-bordeaux.fr

DATA management
Mme Marthe-Aline JUTAND
CeDS EA 74-40
3 ter Place de la Victoire
33076 Bordeaux Cedex
Tel: +33 (0)5 57 57 19 92 / 06 13 05 78 61
Courriel : marthe-aline.jutand@u-bordeaux.fr

Version 4.0 was written on the 18/01/2022, protocole-form : GIRCI SOHO

Update registry

VERSION	DATE	update
1.0	23/01/2024	initial CPP Version
1.0	29/03/2024	Modified Version
2.0	04/06/2024	Re-Modified Version

MAIN CORRESPONDENTS

Coordinating
investigator
Pr Jean-Christophe BERNHARD
Bordeaux University Hospital - Hôpital Pellegrin
Place Amélie Raba Léon
33076 Bordeaux Cedex

Program Manager
Ms Solène RICARD
Department of Urology and Renal Transplantation Bordeaux University Hospital - Pellegrin

Clinical Research Associate
Ms Clémence MORICE
Department of Urology and Renal Transplantation Bordeaux University Hospital - Pellegrin

Methodological Support Unit for Clinical and Epidemiological Research
Medical Information Department, Public Health Unit, Bordeaux University Hospital
146 rue Léo Saignat, case n°75
33076 Bordeaux Cedex FRANCE
Methodological coordination :
Pr Laura RICHERT
Biostatistician :
Mme Roxane COUERON

Unit for Research and Innovation in Care and Human Sciences (URISH)
Ms Hélène HOARAU
Direction des soins et Direction de la Recherche Clinique et de l'Innovation
12, rue Dubernat
rdeaux - Pellegrin

33404 Talence Cedex

Sponsor
Bordeaux University Hospital 12 rue Dubernat
33 400 Talence
FRANCE
Responsible for research at sponsor level
Mr. Gilles DULUC - Director of Clinical Research and Innovation
Dr Anne GIMBERT - Internal Promotion Manager

Clinical Studies Manager
Mrs Aline DOUBLET
Bordeaux University Hospital Clinical Research and Innovation Department
12 rue Dubernat 33404 Talence Cedex

Clinical Research Safety and Vigilance Unit
Clinical Research and Innovation Department 12, rue Dubernat
33404 Talence Cedex

Lab. Culture and dissemination of knowledge (CeDS) EA 7440
Ms Marthe-Aline Jutand
Ms Sarah Masanet
Ms Hélène Hoarau
3 ter Place de la Victoire
33076 Bordeaux Cedex

UroCONNECT tool coordinator
Ms Anne CALLEDE
Department of Urology and Renal Transplantation CHU de Bo

MAIN CORRESPONDENTS

Principal Investigator
Dr Gaëlle MARGUE
CHU de Bordeaux - Hôpital Pellegrin
Place Amélie Raba Léon
33076 Bordeaux Cedex
Tél. : 05 57 82 17 62
Courriel : gaelle.margue@chu-bordeaux.fr

Scientific ManagerPr Jean-Christophe BERNHARD
CHU de Bordeaux - Hôpital Pellegrin
Place Amélie Raba Léon
33076 Bordeaux Cedex
Tél. : 05 57 82 17 62
Courriel : jean-christophe.bernhard@chu-bordeaux.fr

Program Manager
Mme Solène RICARD
Service d'Urologie et Transplantation Rénale
CHU de Bordeaux – Pellegrin
33076 Bordeaux Cedex
Tél. : 05 57 82 12 94
Courriel : solene.ricard@chu-bordeaux.fr

Clinical Research Associate
Mme Clémence MORICE
Service d'Urologie et Transplantation Rénale
CHU de Bordeaux – Pellegrin
33076 Bordeaux Cedex
Tél. : 05 57 82 23 94 - Fax : 05.56.79.56.51
Courriel : clemence.morice@chu-bordeaux.fr

Methodological Support Unit for Clinical and Epidemiological ResearchService d'information médicale, Pôle Santé publique,
CHU Bordeaux
146 rue Léo Saignat, case n°75
33076 Bordeaux Cedex
Tel : 05 57 57 11 29 / 14 42 - Fax : 05 57 57 15 78
Méthodological Coordination :
Pr Laura RICHERT
Courriel : laura.richert@chu-bordeaux.fr
Biostatistics :
Mme Roxane COUËRON
Courriel : roxane.coueron@chu-bordeaux.fr

Unité de la Recherche et de l'Innovation en Soins et Sciences Humaines (URISH)
Mme Hélène HOARAU
Direction des soins et Direction de la recherche clinique et de l'innovation
12, rue Dubernat
33404 Talence Cedex
Tél. : 06 37 83 92 85
Courriel : helene.hoarau@chu-bordeaux.fr

Promotor
Centre Hospitalier Universitaire de Bordeaux
12 rue Dubernat
33400 Talence

Responsible for research at the promoter level
Gilles DULUC - Directeur de la Recherche Clinique et de l'Innovation
Dr Anne GIMBERT - Responsable « Promotion interne »
Tél : 05 57 82 08 34 – Fax : 05 56 79 49 26
Courriel : anne.gimbert@chu-bordeaux.fr

Clinical Studies Manager
Mme Corinne CASTERMANS
Direction de la Recherche Clinique et de l'Innovation du CHU de Bordeaux
12 rue Dubernat
33404 Talence Cedex
Tél. : 05 57 82 08 53
Courriel : corinne.castermans@chu-bordeaux.fr

Unité de sécurité et de vigilance de la recherche clinique
Direction de la recherche clinique et de l'innovation
12, rue Dubernat
33404 Talence Cedex
Tél: 05 57 82 16 26 - Fax: 05 57 82 12 62
Courriel : vigilance.essais-cliniques@chu-bordeaux.fr

Lab. Culture et Diffusion des Savoirs (CeDS)
Mme Marthe-Aline JUTAND et Mme Sarah MASANET
Laboratoire CeDS EA-7440
3 ter, Place de la Victoire
33076 Bordeaux Cedex
Tél. : 05 57 57 19 92 - Mobile : 06 13 05 78 61
Courriel : marthe-aline.jutand@u-bordeaux.fr

UroCONNECT Coordinator
Mme Anne CALLEDE
Service d'Urologie et Transplantation Rénale
CHU de Bordeaux – Pellegrin
Tél. : 05 57 79 60 13 - Fax : 05.56.79.56.51
Courriel : anne.callede@chu-bordeaux.fr

Summary

RESEARCH SUMMARY 9
ABSTRACT 14
1. **SCIENTIFIC JUSTIFICATION AND GENERAL DESCRIPTION** 17
    1.1. CURRENT STATE OF KNOWLEDGE 17
    1.2. RESEARCH HYPOTHESES AND EXPECTED RESULTS 18
    1.3. JUSTIFICATION FOR THE LOW LEVEL OF INTERVENTION 18
    1.4. BENEFIT/RISK RATIO 19
    1.5. EXPECTED RESULTS 20
2. **RESEARCH OBJECTIVES** 20
    2.1. MAIN OBJECTIVE 20
    2.2. SECONDARY OBJECTIVES 20
3. **JUDGMENT CRITERIA** 21
    3.1. MAIN JUDGMENT CRITERION 21
    3.2. SECONDARY JUDGMENT CRITERIA 21
4. **RESEARCH DESIGN** 22
    4.1. JUSTIFICATION OF METHODOLOGICAL CHOICES 22
    4.2. RESEARCH DESIGN 23
    4.3. METHODS FOR RANDOMIZATION 25
5. **ELIGIBILITY CRITERIA** 25
    5.1. INCLUSION CRITERIA 25
    5.2. NON-INCLUSION CRITERIA 25
    5.3. FEASIBILITY AND RECRUITMENT METHODS 25
6. **RESEARCH STRATEGY(IES)/PROCEDURE(S)/PRODUCTS** 26
    6.1. STRATEGY 26
    6.2. EXPERIMENTAL AND COMPARISON PROCEDURE 26
        6.2.1. Personalized 3D Printed Model Group 26
        6.2.2. Generic 3D Printed Model Group 27
7. **RESEARCH PROCEDURE** 27
    7.1. RESEARCH SCHEDULE 27
    7.2. SUMMARY TABLE OF PARTICIPANT FOLLOW-UP 28
    7.3. INCLUSION VISIT (T0) 29
        7.3.1. Consent collection 29
        7.3.2. Visit procedure 29
        7.3.3. Randomization visit/process 30
    7.4. FOLLOW-UP VISITS 30
        7.4.1. Preoperative period (T1) 30
        7.4.2. Exchange and information consultation (T2) 31
        7.4.3. Second preoperative period (T3) 31

7.4.4. Surgery (T4) 31
7.4.5. Postoperative consultation (T5) 31
7.5. END OF RESEARCH VISIT (T6) 31
7.6. STOPPING RULES 32
7.6.1. Stopping a person's participation in the research 32
7.6.2. Stopping the research 32
7.7. DEVIATIONS FROM THE PROTOCOL 33
7.7.1. Premature and definitive termination of the research procedure 33
7.7.2. Participant lost to follow-up 33
7.7.3. Participant wrongly included 33
7.8. SIMULTANEOUS PARTICIPATION IN OTHER RESEARCH, EXCLUSION PERIOD, **COMPENSATION AND REGISTRATION IN THE VRB FILE 33**
8. **MANAGEMENT OF ADVERSE EVENTS / SIDE EFFECTS / INCIDENTS** 33
9. **STATISTICAL ASPECTS** 34
9.1. STUDY SIZE 34
9.2. STATISTICAL METHODS USED 34
9.2.1. Analysis strategy 34
9.2.2. Patients included in the analysis 35
9.2.3. First-type risk 35
9.2.4. Descriptive statistical methods 35
9.2.5. Comparative statistical methods 36
9.2.6. Qualitative data analysis methods 36
9.2.7. Software used 36
9.3. ANALYSIS PLAN 36
9.3.1. Description of inclusions, deviations and follow-up 36
9.3.2. Characteristics of patients at inclusion 36
9.3.3. Analysis of the primary objective 37 9.3.4. Analysis of secondary objectives 37
10. **RESEARCH MONITORING** 38
10.1. SCIENTIFIC COUNCIL 38
10.1.1. Composition 38
10.1.2. Frequency of meetings 38
10.1.3. Role 38
10.2. INDEPENDENT MONITORING COMMITTEE 39
11. **MANAGEMENT AND PROCESSING OF SOURCE DATA AND DOCUMENTS** 39
11.1. SOURCE DATA AND DOCUMENTS 39
11.2. INSTRUCTIONS FOR DATA COLLECTION 39
11.3. DATA MANAGEMENT AND CIRCUIT 39
11.3.1. Data management software 39
11.3.1.1. Software used 39
11.3.1.2. Data hosting 40
11.3.1.3. Data security 40
11.3.2. Data entry 40
11.3.3. Data coding 41
11.3.4. Data controls 41
11.3.5. Data transfer 41

11.4. DATA CONFIDENTIALITY 41
11.5. RETENTION OF RESEARCH DOCUMENTS AND DATA 42
11.6. DATA TRANSFER 42
12. **QUALITY CONTROL AND ASSURANCE** 43
12.1. DATA ACCESS 43
12.2. QUALITY CONTROL 43
12.3. AUDIT AND INSPECTION 43
13. **ETHICAL AND REGULATORY CONSIDERATIONS** 44
13.1. COMPLIANCE WITH REFERENCE TEXTS 44
13.2. MODIFICATIONS TO THE PROTOCOL 44
14. **FINAL REPORT** 45
15. **RULES RELATING TO PUBLICATION** 45
15.1. SCIENTIFIC COMMUNICATIONS 45
15.2. COMMUNICATION OF RESULTS TO PARTICIPANTS 45
BIBLIOGRAPHICAL REFERENCES 46

ABREVIATION LIST

ANSM : French National Agency for the Safety of Medicines and Health Products

CeDS: Culture et Diffusion des Savoirs (Culture and Dissemination of Knowledge)

CNIL: Commission Nationale de l'Informatique et des Libertés (French Data Protection Authority)

CPP: Comité de Protection des Personnes (Committee for the Protection of Individuals)

CREDIM: Centre for Research and Development in Medical Informatics

DM: Data Manager

DREES: Direction de la Recherche, des Etudes, de l'Evaluation et des Statistiques (Research, Studies, Evaluation and Statistics Department)

HDS: Health Data Hosts

HLSEU-Q16: European Health Literacy Survey Questionnaire

ITT: Intention-to-treat

IUT: Institut Universitaire de Technologie (University Institute of Technology)

WHO: World Health Organisation

R3DP-P: 3D Print-Personalize Kidney Study

RCP: Réunion de Concertation Pluridisciplinaire (Multidisciplinary Consultation Meeting)

SHS: Human and Social Sciences

URISH: Unité de la Recherche et de l'Innovation en Soins et Sciences Humaines (Research and Innovation Unit for Care and Human Sciences)

UroCCR: French Kidney Cancer Research Network

USMR: Methodological Support Unit for Clinical and Epidemiological Research

RESEARCH PROMOTION

PROMOTOR	Centre Hospitalier Universitaire de Bordeaux<br>12, rue Dubernat, 33400 Talence, France
COORDONNATING INVESTIGATOR	Dr Gaëlle MARGUE
Scientific lead	Pr Jean-Christophe BERNHARD
Acronym, Title	R3DP-P - Effects of a Personalized or Generic Three-Dimensional Tumoral Kidney Model on Patient Experience and Caregiver-Patient Interactions Before and After Partial Nephrectomy
JUSTIFICATION / CONTEXT	A 2015 pilot study showed the benefit of using custom 3D-printed kidney models as an educational mediation tool during the pre-operative consultation of patients scheduled for robot-assisted partial nephrectomy. This made it easier to understand the pathology and the surgery. Patients were better able to grasp the surgical implications of their disease, which led to greater satisfaction with their care.<br>The patients we treat come from a variety of cultures and social backgrounds. It is therefore important to take this heterogeneity into account in the care provider-patient relationship, laying the foundations for personalised medicine. Measuring health literacy makes it possible to identify and describe this heterogeneity in patient profiles. It has been shown that patients with a low level of literacy have a higher level of anxiety, particularly about surgery, and a poorer level of post-operative recovery. Numerous studies have been carried out to develop strategies for improving patients' health literacy and the information provided by carers. More specifically, they stress the importance of improving understanding of the information communicated to patients.<br>The use of a 3D-printed kidney model, whether personalised or generic, during a specific pre-operative information consultation with the patient, could therefore change the patient's understanding of his or her pathology and its management, as well as his or her interactions with all healthcare professionals.
OBJECTIVES	Main objective:<br>To study the effects of using a personalised 3D printed kidney model versus a generic 3D printed kidney model as a mediation tool, throughout the care pathway, on patients' experience and interactions with professionals, before and after partial nephrectomy.

	Secondary objectives :<br>1. To use qualitative studies to describe interactions between professionals and patients, specifying the situations in which exchanges take place and their environments.<br>2. To use qualitative studies to compare differences in discourse and the use of terms in the use of different tools during interactions between patients, professionals and family and friends.<br>3. To use a qualitative approach to study the use of the 3D-printed kidney model introduced during a medical information consultation right through to the post-operative visit.<br>4. To compare patient understanding of anatomy and surgical strategy according to the type of tool, personalised or generic, before and after the operation on the basis of questionnaires and interviews.<br>5. To compare the level of health literacy between the two groups and at different times (HLSEU-Q16: European Health Literacy Survey Questionnaire).<br>1. 6. To use qualitative studies to describe the changes perceived by professionals in patient care following the integration of the patient mediation tool.
ASSESSMENT CRITERIA	MAIN JUDGING CRITERION :<br>Mixed criterion used to describe the patient experience based on the interviews. Observations and the three questionnaires used complete the qualitative data analyses. The patient experience will also be analysed in relation to the clinical and demographic data from the UroCCR database.<br><br>SECONDARY JUDGEMENT CRITERIA :<br>1. Conduct of interactions between professionals and patients: description of the conduct of interactions according to the model used, specifying the practice and the vocabulary and content of the discourse used.<br>2. Frequency of terms used in accordance with the use of the various tools during interactions.<br>3. Use of the 3D model during care: description of the life of the model throughout care (from the inclusion visit to the post-operative visit).<br>4. Level of understanding of renal anatomy and surgical issues.<br>5. Change in the mean score of the HLSEU-Q16 literacy questionnaire measured (health literacy).<br>1. 6. Effects on professionals: qualitative assessment of changes in practice following the integration of 3D models into the department's practice.

RESEARCH scheme	Single-centre, mixed (qualitative and quantitative) study based on observations (interaction situations), interviews (professionals and patients) and questionnaires (patients).

	Patients<br>- Interventional, prospective, randomised study in a 1:1 ratio, in 2 parallel open arms: one with a preoperative visit with a customised 3D printed kidney model, the other with a generic 3D printed kidney model.<br><br>Professionals<br>Non-interventional study of the experience of professionals in the management of patients who have benefited from a pre-operative visit using a personalised or generic 3D printed kidney model.
INCLUSION CRITERIA	Patients<br>- Age ≥ 18 years<br>- Scheduled surgical management by laparoscopic partial nephrectomy with robotic assistance (1st management for unilateral or bilateral kidney tumour)<br>- Free, informed and signed consent for the database UroCCR<br>- Free, informed and signed consent for the Rein3D Personalize protocol<br>- Person affiliated to or benefiting from a social security scheme<br><br>Professionals<br>- Professionals working with patients treated in the Urology, Andrology and Renal Transplant Department of Bordeaux University Hospital for at least two months prior to the implementation of 3D tools<br>- Free, informed and signed consent
NON INCLUSION CRITERIA	Patients<br>- Metastatic at inclusion<br>- Previous kidney cancer<br>- Single kidney at the time of inclusion<br>- No preoperative CT scan or poor quality CT scan not allowing reliable 3D modelling<br>- Person under legal protection (safeguard of justice, guardianship or curatorship)<br>- Difficulty understanding and expressing themselves in French<br><br>Professionals<br>Professional on work placement for less than 6 months<br>Professional with no contact with patients

RESEARCH STRATEGIES/PROCEDURES	Patients<br>On inclusion in the study, patients will be randomised into 2 arms, corresponding to the different information media used during the information consultation:

	1. Personalised 3D printed model group: discussion time with the printed three-dimensional physical model of the kidney to be operated on as information support<br>2. Generic 3D printed model group: discussion time based on a three-dimensional printed physical model, taken from a case of eight models, and corresponding as closely as possible to the patient's situation.<br><br>Professionals<br>No specific intervention
CONDUCT OF THE STUDY	Patients: 60 included<br>- An initial consultation with the surgeon between 1 and 6 months before surgery for patient inclusion.<br>- An interview with the SHS researcher within 15 days of the inclusion visit, and completion of the HLSEUQ16 questionnaire and the knowledge questionnaire.<br>- A second consultation with the surgeon for information using the personalised or generic 3D printed model depending on the randomisation group, between 7 and 30 days before surgery.<br>- A second interview with the SHS researcher within 15 days of the second consultation, and completion of the knowledge questionnaire.<br>- A post-operative consultation 1 month after surgery, followed by a final interview with the SHS researcher and completion of the HLSEUQ16 questionnaire, the knowledge questionnaire and the satisfaction questionnaire.<br><br>Professionals: 30 inclusions<br>- - 1 individual interview per professional in the year following the start of the study
STUDY SIZE	60 patients et 30 professionnals
STUDY DURATION	Length of inclusion period: 18 months<br>Duration of participation for patients: between 3 and 6 months<br>Duration of participation for professionals: 24 months<br><br>Total duration of research: 38 months (3 years and 2 months) inclusion, follow-up, analysis and evaluation.

STATISTICAL ANALYSIS OF DATA	Patients' socio-demographic and clinical data will be described in terms of frequencies for qualitative data and means and standard deviations for quantitative data, enabling comparisons to be made between the two groups of patients.<br>Data from questionnaires completed by patients will be analysed descriptively (distribution of scores obtained by patients for the HLS-EU16 literacy questionnaire at the beginning and at the end, description of the satisfaction questionnaire at the last stage). This
	A descriptive analysis will be carried out overall and by randomisation group.<br>A comparative analysis between the randomisation groups will be carried out, systematically without adjustment and with adjustment for the initial prognostic factors whose distribution could be unbalanced despite randomisation.<br>The patient experience will be described on the basis of a cross-sectional analysis of the content of the interviews at each stage, as well as a longitudinal analysis. Changes in understanding of the disease and surgery will be studied by analysing the content of the interviews and questionnaires, and a comparison will be made between the two groups.<br>The professionals' interviews will also be analysed for content.<br>All the analyses will be carried out with the support of Nvivo software and will be performed by the SHS researchers.<br>The data will be analysed by the team of SHS researchers to ensure cross-analysis of the qualitative and quantitative data.<br>The main analysis will be carried out by sub-treatment for the patients, i.e. the data from all the participants must be analysed with regard to the 3D printed model actually administered, even if randomisation has decided otherwise, on the basis of available data.

EXPECTED BENEFITS	The expected outcomes of the R3DP-P mixed (qualitative and quantitative) study are :<br>For patients o Acquire knowledge on the contribution of the materialization of the pathology in its understanding.<br>o To assess patients' appropriation of the 3D tool as a means of mediation and understanding (literacy).<br>o To improve the patient's experience of hospitalisation and surgery.<br><br>For professionals o To assess the appropriation of the 3D tool as a mediation tool by professionals.<br><br>For the healthcare system o To identify specific uses depending on whether or not the model is personalised, and to assess the benefits of personalising the 3D tool and the value of implementing it in everyday practice.

ABSTRACT

This research has been registered in http://www.clinicaltrials.gov/ under the n° NCT06379698

Effects of a personalized or generic three-dimensional tumoral kidney model on patient experience and professional-patient interactions, before and after partial nephrectomy. R3DP-P

The University Hospital of Bordeaux is the sponsor of this research.

This research will be conducted with the support of ANR-21-RHUS-0015.

Brief summary: We aim to compare preoperative information and patient experience using a personalized versus a generic 3D printed models of patients' tumoral kidney before and after nephron-sparing surgery. The main outcome measure will be based on semi-structured interviews with the patient and the carers.

Detailed description: Use of tools to decrease anxiety and enhance understanding prior to surgery is a key point in comprehensive care that is way not enough promoted for now.
A pilot study from 2015 demonstrated the benefits of using personalized 3D-printed kidney models as educational mediation tools during the pre-operative consultation of patients scheduled for robot-assisted partial nephrectomy. The patient's understanding of his pathology and of the surgery was thus facilitated, leading to greater satisfaction during treatment.

For personalized medicine, it is important to consider the heterogeneity in culture and social backgrounds of patients in the doctor-patient relationship. Measuring health literacy help to identify and describe this heterogeneity in patient profiles. Patients with low literacy levels have higher levels of anxiety, particularly regarding surgery, and poorer post-operative recovery. Some studies highlight the importance of improving patient understanding with strategies to improve patients' health literacy and information provided by carers.

The aim of this study is therefore to investigate the benefits of using a personalized 3D-printed kidney model versus a generic 3D-printed kidney model as a mediation tool, all along the care pathway, on patients' experience and their interactions with carers, before and after partial nephrectomy.

To achieve this aim, 60 patients planned for robot-assisted laparoscopic partial nephrectomy will be randomized, in a 1:1 ratio, between the use of a personalized 3D kidney model and a generic 3D kidney model.

3D models will be presented to the patients according to the allocated study group during a preoperative education consultation. All patients will complete questionnaires about their health literacy level, their knowledge of kidney anatomy and tumor and their satisfaction about the using 3D-printed kidney models as educational mediation tools. Semi-structured interviews will be conducted at three different times: between the first consultation with the surgeon and the preoperative education consultation, between this second consultation and the surgery and after the post-operative consultation.

Primary outcome:

To study the effects of using a personalized versus a generic 3D-printed kidney model as a mediation tool on patients' experience and interactions with carers, before and after partial nephrectomy.

This is a mixed-type study designed to combine data from different collection tools (observations, interviews and questionnaires). The criteria studied are:

For the patient:

- Qualitative assessment of the patient experience
- Changes in the average score of the HLSEU-Q16 literacy questionnaire - Level of understanding of renal anatomy and of the surgical issues

For the carers:

- Qualitative assessment of changes in practice following the integration of 3D models into the department

Secondary outcomes:

1. Description of interactions between carers and patients, specifying the situations and the environments, the practice, vocabulary and content of the discourse used during the entire care process.
2. Comparison of the differences in speech and terminology used when using the different tools during interactions between patients, carers and patients' entourage with systematic analysis of interviews.
3. Studying the use of the 3D-printed kidney model introduced during the medical information consultation up to the post-operative visit by describing the life of the model throughout the entire care process (from the inclusion visit to the post-op visit).
4. Comparison of the patient's understanding of anatomy and surgical strategy according to the type of tool (personalized vs generic 3D printed model) before and after the surgery (T1, T3 and T6).
5. Comparison of the level of health literacy between the two groups at different times with changes in the average score on the literacy questionnaire (HLSEU-Q16: European Health Literacy Survey Questionnaire; T1 and T6).
6. Description of the changes perceived by carers in patients management following the integration of the mediation tool with patients by qualitatively assessing changes in practices following the integration of 3D models during the entire care process.

Study design: Monocentric, randomized and controlled clinical trial Eligibility criteria:

- Patients:

- Adult patients (≥ 18 years of age)
    - Scheduled for surgical management by laparoscopic robotic-assisted partial nephrectomy (1st management for a unilateral or bilateral kidney tumor)
    - Expressed consent for integration in the UroCCR database o Expressed consent for participation in the Rein-3D Personalize study o Patients affiliated or benefiting from social security system

- Carers:
    - Carers working with patients treated in the Urology, Andrology and Renal Transplant Department of the Bordeaux University Hospital for at least two months prior to the implementation of 3D models
    - Free, informed and signed consent

Arm number or label and arm type:

- Group 1: 3D Printed Personalized Model Group: exchange time with the printed threedimensional model of the patient's kidney as information support.
- Group 2: 3D Printed Generic Model Group: exchange time with the printed threedimensional generic kidney model, approximating the patient's situation, as information support.

Number of subjects: 60 patients and 30 carers -

Statistical analysis :

A mixed analysis of the data will be realised with a triangulation approach. The sociodemographic data and the medical profiles of the patients will be described (in terms of frequencies for the qualitative data and with means and standard deviations for the quantitative data) and will be compared between the two groups. Data from patients questionnaires will be analysed descriptively (distribution of patient scores for the HLS-EU16 litteracy questionnaire at start and end and at the end distribution of satisfaction with use of the 3D model.). The analysis of the patients' experience will be based on the content of the cross-sectional interviews at each stage, but also on a longitudinal analysis. The evolution of the understanding of the disease and the surgery will be studied by analysing the content of the interviews and questionnaires and a comparison between the two groups will be made.

Content of the carers interviews will also be analysed.

Conditions: kidney cancer surgery

Keywords: Renal-Cell Carcinoma, Nephron-Sparing Surgery, 3D Model, 3D Printing, Personalized Medicine, Literacy Questionnaire, Interviews.

1. SCIENTIFIC JUSTIFICATION AND GENERAL DESCRIPTION

1.1 CURRENT STATE OF KNOWLEDGE

In 2015, a pilot study evaluated the use of a personalised 3D-printed kidney model in 7 English-speaking patients who were being considered for partial nephrectomy (1). It was shown that these 3D models could be used as educational mediation tools to make it easier for patients to understand their pathology and surgical management.

Better communication would give patients a better understanding of the issues involved in surgery and give them greater satisfaction with the course of their visits and medical follow-up (2).

However, as the study by Curchod et al. points out, 'the care provided in our society is aimed at people from diverse and varied cultures and social backgrounds' (3). It is therefore essential to take account of this heterogeneity in the care-giver-patient relationship, thus defining the notion of personalised medicine. Health literacy, defined by the WHO as 'the cognitive and social skills that determine people's motivation and ability to obtain, understand and use information in ways that promote and maintain good health', makes it possible to identify and describe this heterogeneity in patient profiles.

According to a study by the DREES (Direction de la recherche, des études, de l'évaluation et des statistiques), published in May 2023, 10.7% of the population consider that they have difficulty understanding medical information (4). In the case of surgery, it has been shown that patients with a low level of literacy have a higher level of anxiety (5), particularly in relation to the operation (6), and a poorer level of post-operative recovery (7). Numerous studies have been carried out to develop strategies for improving the health literacy of cancer patients and information methods for carers (8-10). They emphasise the importance of improving understanding of the information given to patients.

A patient diagnosed with a kidney tumour will meet a number of different healthcare professionals during his or her hospital stay. The interactions that will take place between the patient and each professional will depend on the place, the context, the patient's personal characteristics (age, gender, profession, literacy level, etc.) and also the various conceptions that the professional has of the patient as a subject. For example, if the patient is seen as a player in his or her own care, i.e. able to acquire and develop care skills through the professional-patient relationship, then professionals' expectations of the patient and his or her interpersonal skills will be high (11).

The introduction of new technologies into patient care can therefore change the way professionals behave (12). For example, the use of a 3D-printed kidney model can change the patient's understanding of his pathology and its management, as well as his interactions with all the professionals involved.

In 2022, the study by Scott et al, based on a protocol similar to that of Bernhard et al (1) and using the same comprehension questionnaire, confirmed the impact of these personalised models on patient comprehension, while highlighting the financial impact of their manufacture (13). The study by Bernhard et al. used personalised 3D printed models, i.e. a reproduction of the patient's kidney made from the preoperative scan (1). However, the cost and time taken to produce these models may make it difficult to incorporate them into clinical practice during the preoperative consultation. It therefore seems worthwhile evaluating the use of different models that are not personalised but represent the different tumour situations that are classically found.

The addition of a specific pre-operative information consultation, using a personalised or generic 3D printed model of the kidney, can have a different impact (depending on the model) on the patient's experience, their level of literacy and their interactions with healthcare professionals.

This study is part of the RHU Digital Urology 3D programme, which includes several studies aimed at assessing the contribution of 3D models in patients undergoing robot-assisted laparoscopic partial nephrectomy (e.g. the ANXIETY study conducted by Bordeaux University Hospital, NCT06035211). The Rein 3D Print-Personalize (R3DP-P) study will evaluate the information situations throughout the patient's care in the Urology department, depending on the type of model used during the pre-operative information consultation (generic or personalised), using an anthropological approach.

1.2. RESEARCH HYPOTHESES AND EXPECTED RESULTS

The R3DP-P study evaluates the personalised aspect of the 3D-printed tool, its interest for the patient (diagnosis of a renal tumour accessible to conservative surgical treatment) and its impact on his understanding of the disease and its management, as well as on his relations with healthcare professionals.

The main hypothesis is that the personalised aspect of the 3D-printed model leads to the use of the model as a mediation tool during the patient's social interactions with family and friends and with healthcare professionals to discuss the disease, or during the development of knowledge. This use would reflect an appropriation of the personalised 3D model.
In addition, it is assumed that the patient's observation and manipulation of the 3D representation of 'their' kidney would enable them to better understand their pathology throughout the care pathway.

The R3DP-P study is based on a didactic health anthropology approach, where the reflexivity of the principal investigator and the field are the driving force behind the scientific method. The working hypotheses are as follows:

- Hypothesis 1: The 3D-printed personalised kidney model enables patients to 'better' understand their pathology and the surgical procedure.

- Hypothesis 2: The 3D-printed personalised kidney model makes it easier for patients to communicate with their family and friends about their condition.

- Hypothesis 3: The 3D printed personalised kidney model improves the therapeutic alliance between patients and professionals.

- Hypothesis 4: The 3D-printed personalised kidney model leads to a change in professionals' information practices.

1.3. JUSTIFICATION FOR THE LOW LEVEL OF INTERVENTION

The nature of the R3DP-P study does not involve any risks and is associated with minimal constraints for patients.

This research does not entail any change in the patient's indications and clinical management strategies, as validated by the surgical team in accordance with current recommendations and the conclusions of the multidisciplinary consultation meeting (RCP).

Only one preoperative information consultation will be added to the patient's conventional care, 7 to 30 days before the operation, delivered by a doctor: this will be a specific time, mediated by a 3D printed model, dedicated to informing the patient.

As for the professionals who have agreed to take part, the interviews will be conducted by the SHS researchers at their convenience and in the place of their choice, in order to make it easier for them to talk, not to interfere with their care and not to disrupt their daily lives.

The research method is based on interviews, questionnaires to understand the pathology and observations. If, during an interview with a patient or a professional, a researcher notes a need for psychological support, she will refer the participant to a healthcare professional. The SHS researchers will act as observers to understand the different situations of interaction between patients and professionals and will ensure that they never disrupt the situations (particularly consultations). An oral request will always be made to obtain the agreement of the protagonists in the situation being observed.

1.4. RISK/REWARD RATIO

The main expected benefit is improved understanding of preoperative information, an essential condition for better preparation before surgery.

Patients

The addition of a mediated consultation to the care pathway can also be seen as a benefit, as it gives patients the opportunity to ask all their questions before the operation. It does, however, oblige the patient to return to the care centre for an additional period of time.

As the study intervention consists of taking part in research interviews and answering (non-diagnostic) questionnaires, there is no physical risk during participation.

Nevertheless, it is possible that asking questions related to the presence of renal tumours could lead to other questions being asked. Indeed, the questions question the patient's feelings. It is conceivable that the patient will then begin a process of introspection that he or she had not experienced before, which may have psychological consequences that are considered to be moderate. It should be emphasised that the patient's overall care is part of a care network that includes close nursing support and contacts (particularly psychologists) available throughout the patient's follow-up.

Professionals

The R3DP-P study protocol is qualitative and non-interventional, based on observations and interviews with no expected effect on professionals or patient management.

1.5. EXPECTED BENEFITS

The expected outcomes of the R3DP-P mixed (qualitative and quantitative) study are :

- For patients o Acquire knowledge on the contribution of the materialization of the pathology in its understanding.

o Evaluate the appropriation of the 3D tool as a mediation and comprehension (literacy) tool by patients.

o To improve patients' experience of hospitalisation and surgery.

- For professionals

o To assess the appropriation of the 3D tool as a mediation tool by professionals.

- For the healthcare system :

o Identify specific uses depending on whether or not the model is personalised, and assess the benefits of personalising the 3D tool and the value of implementing it in everyday practice.

2. RESEARCH OBJECTIVES

2.1. MAIN OBJECTIVE

To use qualitative studies to investigate the effects of using a personalised 3D-printed kidney model versus a generic 3D-printed kidney model as a mediation tool, throughout the care pathway, on patients' experience and their interactions with professionals, before and after partial nephrectomy.

2.2. SECONDARY OBJECTIVES

1. To use qualitative studies to describe interactions between professionals and patients, specifying the situations in which exchanges take place and their environments.

2. To use qualitative studies to analyse differences in discourse and the use of terms in the use of different tools during interactions between patients, professionals and their families.

3. To use a qualitative approach to study the use of the 3D-printed kidney model introduced during a medical information consultation right through to the post-operative visit.

4. To compare patient understanding of anatomy and surgical strategy according to the type of tool, personalised or generic, before and after the operation on the basis of questionnaires and interviews.

5. To compare the level of health literacy between the two groups and at different times (HLSEU-Q16: European Health Literacy Survey Questionnaire).

6. To use qualitative studies to describe the changes perceived by professionals in patient care following the integration of the mediation tool with patients In order to meet the objectives set out above, the study will focus on two populations: patients and professionals.

3. ASSESSMENT CRITERIA

3.1. MAIN ASSESSMENT CRITERIA

Qualitative assessment of the patient experience

This is not a single criterion, but a set of elements used to describe the patient experience based on the interviews. Observations and the three questionnaires used (literacy, knowledge and satisfaction questionnaires: appendices 1, 2 and 3) complete the analyses of the qualitative data. The patient experience collected will also be analysed in relation to the clinical and demographic data from the UroCCR database.

3.2. SECONDARY ASSESSMENT CRITERIA

Course of interactions between professionals and patients: description of the course of interactions according to the model used, specifying the practice as well as the vocabulary and content of the discourse used.

Frequency of terms used in accordance with the use of the different tools during interactions: comparison of the differences in discourse and terminology when using the different tools

during interactions between patients, carers and the patient's entourage with the systematic analysis of the interviews.

Use of the 3D model during care: description of the life of the model throughout care (from the inclusion visit to the post-operative visit).

Level of understanding of renal anatomy and surgical issues measured using the translation of the questionnaire proposed by Bernhard et al (1) (appendix 2). This questionnaire consists of 4 parts: physiological and anatomical dimensions, knowledge of the disease and tumour characteristics, understanding of the surgical procedure and associated risks of complication. These dimensions will be studied before and after the presentation of the model and compared according to the model used. The final part of the questionnaire, based on the evaluation of patient satisfaction, will be presented at the end of the post-operative consultation period (T6) (Appendix 3).

Evolution of the mean score of the HLSEU-Q16 literacy questionnaire measured (health literacy)

The evolution of the literacy score according to the type of mediation tool used during the information consultation will be assessed by measuring the HLSEU-Q16 at inclusion and 1 month post-operatively. This questionnaire is based on a model including four skills related to the processing of health information: access, understanding, evaluation and application of health information (Appendix 1). These four skills are explored in three health contexts: health care, disease prevention and health promotion. The HLSEU-Q16 is composed of 16 items. All dimensions are explored except for the dimension "applying information in health promotion". This short version was validated in French by Rouquette et al. in 2018 (15). Each item is rated on a four-level scale: "very easy", "easy", "difficult" and "very difficult". For the rating, the score of each item is reduced to a binary score, the "very easy" and "easy" modalities scoring for 1 while "difficult" and "very difficult" for 0. The total score can then vary from 0 to 16. To facilitate interpretation, they can be classified into three literacy levels: less than or equal to 8 reflecting an inadequate level, from 9 to 12 reflecting a problematic level and strictly greater than 12, an adequate level.

The distribution of the three score levels will be described preoperatively and postoperatively in each arm.

Validated in French, this short scale of the "European Health Literacy Survey Questionnaire" (14) has acceptable psychometric qualities and allows a reduced administration time for participants, the latter having several questionnaires to complete at the same time as part of the study.

Effects on professionals

Qualitative evaluation of changes in practices following the integration of 3D models into the practice of the service.

4. RESEARCH DESIGN

4.1. JUSTIFICATION OF METHODOLOGICAL CHOICES

Choice of the mixed approach

The study aims to understand the patient experience when introducing a new tool in caregiver-patient communication, throughout the care pathway. The introduction of a new mediation tool in patient care can impact patient-professional interactions. It is therefore necessary to understand how this tool, whether personalized or generic, will support the patient throughout their care.

This is why a comprehensive approach to the interactions between patient/professional and patient/entourage, during the informational and educational processes mediated by a personalized or generic tool, is indicated as a study design.

Also, the R3DP-P study is anchored in a comprehensive approach to the interactions between patients and professionals, as developed in health anthropology (16,17):

- Ethnographic approach (i.e. based on observations of information situations, particularly during interactions between professionals/patients, professionals/professionals, patients/patients, etc.) and more generally anthropological (based on individual semi-directive interviews exploring the patients' experience, health education, health literacy) (18).

- Educational Sciences approach (based on the same interviews and observations) to understand the information situations that arise during the patient's journey, describe the knowledge dissemination and knowledge construction circuits of the different actors, to understand the transposition work carried out by professionals during interactions with patients (19).

In addition, a more quantitative approach complements the comprehensive and didactic approach and allows us to question the impact of the integration of the model on the patient's understanding of the pathology and their level of health literacy. This approach will be carried out via the validated HLSEU-Q16 literacy questionnaire, the pathology understanding and satisfaction questionnaire, created internally, and taken up internationally in the Scott study (13) (appendix 1, 2 and 3).

4.2. STUDY SCHEME

This is a single-center study, conducted within the Urology, Andrology and Renal Transplantation Department of the Bordeaux University Hospital. It is a mixed study (qualitative and quantitative) based on observations (interaction situations), interviews (professionals and patients) and questionnaires (patients).

Patients

- Interventional study, prospective randomized, in 2 parallel open arms: one having a preoperative visit with a personalized 3D printed kidney model, the other with a generic 3D printed kidney model,
- Prospective inclusion.

Patients will be randomized with a 1:1 ratio to two groups:

- Group 1: Personalized 3D printed model group,
- Group 2: Generic 3D printed model group.

Professionals

Non-interventional study of the experience of professionals when caring for patients who have benefited from a preoperative visit using a personalized or generic 3D printed kidney model. Note that a study of the experience of professionals before the implementation of 3D models

was carried out as part of the Rein-3D Print PERSONALIZE– Pro Before– Hors Loi Jardé project (reference number CER-BDX 2023 – 64).

STUDY SCHEME

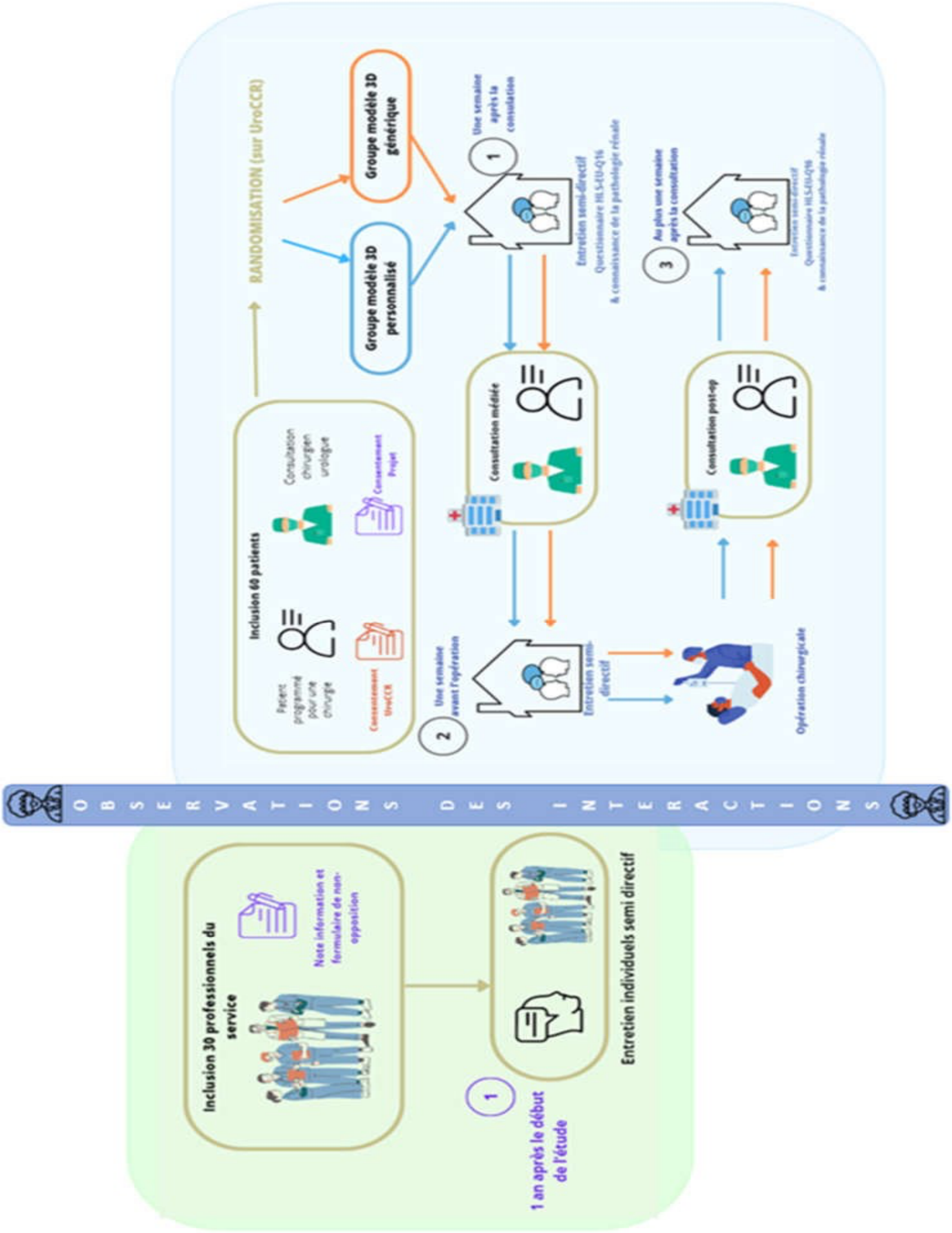

4.3. RANDOMISATION METHOD

The randomization list is established by the statistician of the Methodology Center (USMR of the Bordeaux University Hospital) before the start of the research.

The numbers of the 2 strategy groups are balanced with a 1:1 ratio, without stratification factor. A document describing the randomization procedure is kept confidentially within the Methodology and Data Management Center.

5. ELIGIBILITY CRITERIA

5.1. INCLUSION CRITERIA

Patients

- Age ≥ 18 years,
- Scheduled surgical management by coelioscopic partial nephrectomy with robotic assistance (first management for a unilateral or bilateral kidney tumor),
- Free, informed and signed consent for the UroCCR database,
- Free, informed and signed consent for the Rein-3D Personalize protocol,
- Person affiliated or beneficiary of a social security scheme.

Professionals

- Professional working with patients treated in the Urology, Andrology and Renal Transplantation department of the Bordeaux University Hospital for at least two months before the implementation of the 3D tools,

- Free, informed and signed consent.

5.2. EXLUCSION CRITERIA

Patients

- Metastatic at the time of inclusion,

- History of kidney cancer,

- Single kidney at the time of inclusion,

- Absence of preoperative CT scan or poor quality CT scan not allowing reliable 3D modeling,

- Person subject to a legal protection measure (judicial safeguard, guardianship or curatorship),

- Difficulty understanding and expressing oneself in French.

Professionals

- Professional on an internship of less than 6 months,

- Professional without contact with patients.

5.3. FEASIBILITY AND RECRUITMENT TERMS

This is a single-center study including patients treated in the Urology, Andrology and Renal Transplantation Department of the Bordeaux University Hospital. The patients are all included in the UroCCR database and, given the prospective inclusions recorded in the database in 2022, the inclusion potential is much higher than the expected 4 patients/month (223 patients operated on for robot-assisted partial nephrectomy in 2022).

RECRUITMENT METHODS
Patients
Patient inclusion will be prospective and will take place after the consultation with the urologist.
All adult patients meeting the inclusion criteria and scheduled for a laparoscopic partial nephrectomy with robotic assistance for a kidney tumor will be offered the study.
The principal investigator will provide the participant with an information note on the study and a consent form.

Professionals
All professionals meeting the inclusion criteria who care for patients monitored in the Urology, Andrology and Renal Transplantation Department of the Bordeaux University Hospital will be included.
The SHS researchers or the principal investigator will present the study to the professionals themselves and give them the information note relating to the study including a consent form.

6. RESEARCH STRATEGY(S)/PROCEDURE(S)/PRODUCTS

6.1. STRATEGY

Patients

Upon inclusion in the study, patients will be randomized into 2 arms, corresponding to the different information media used during the information consultation:

1. Personalized 3D printed model group: discussion time with the physical three-dimensional model printed of the kidney to be operated on as information support

2. Generic 3D printed model group: discussion time with a physical three-dimensional model printed as information support, from a case of eight models, and corresponding as closely as possible to the situation

Professional population

No specific intervention

6.2. EXPERIMENTAL AND COMPARISON PROCEDURE

6.2.1 CUSTOM PRINTED 3D MODEL GROUP

For patients randomized to the “personalized” group, a 3D model of the kidney and tumor will be performed by the surgeon, based on the preoperative scan, using Synapse 3D software (Fujifilm). This model will then be printed using the Stratasys printer acquired in partnership with the IUT of Bordeaux and located on the IUT site (sending of the 3D files (.stl) anonymized by the software to the IUT engineer in charge of printing). The 3D model printing time is approximately 15 days after the scans are performed.

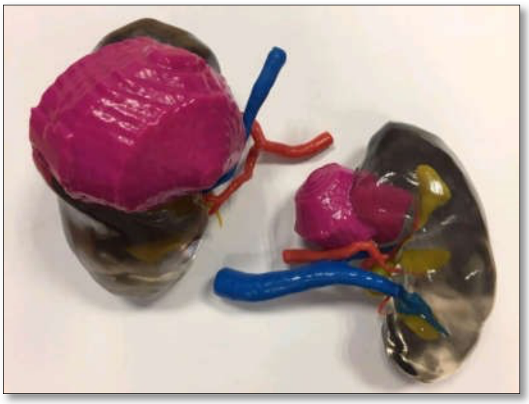

3D TUMOR KIDNEY MODELS

For group 1, the 3D printed personalized model is presented to the patient by the surgeon during a dedicated consultation between D-30 and D-7 before the operation, accompanied by anatomical comments and simple surgical strategies. During this visit, the patient will then be able to take charge of and manipulate the personalized model.

6.2.2 GENERIC PRINTED 3D MODEL GROUP

For patients randomized to the "generic" group, the surgeon has a case containing 8 3D-printed kidney models (made using the Stratasys printer acquired in partnership with the IUT of Bordeaux and located on the IUT site).
These 8 models cover:
- The different locations: superior, equatorial and inferior; anterior and posterior; external border and internal border,
- Hilar tumors,
- The endophytic or exophytic characteristic,
- The different sizes: cT1a, cT1b and cT2,
- Modal vascularization or the presence of a 2nd artery.

The surgeon will select from the generic models the one that most closely matches the patient's situation based on the size and location of the kidney tumor. The 3D printed generic model will then be presented to the patient by the surgeon during a dedicated consultation between D-30 and D-7 before the operation, accompanied by anatomical comments and simple surgical strategies. The patient will then be able to take charge of and manipulate the generic model.

7. <u>RESEARCH PROCESS</u>

7.1 RESEARCH CALENDAR

Patients

- Duration of the inclusion period - 18 months, - Duration of participation - between 3 and 6 months.

Professional population

Participation throughout the study, as part of the observation of patient pathways. Duration of participation for professionals: 24 months.

Total duration of the research: 38 months (inclusion, monitoring, analysis and valorization).

7.2 SUMMARY TABLE OF PARTICIPANT MONITORING

Patients

	STUDY PERIOD							
	Enrolment	Allocation	Post-allocation				Close-out	
**Timepoint**	**T0** (First consultation)	**T1** (T0 + 1 day)	(T1 + 15 days)	**T2** (30 to 7 days before D0)	**T3** (T2 + 15 days)	**T4** (D0)	**T5** (T4 + 15 to +45 days)	**T6** T5+15 days
**ENROLEMENT**								
**Eligibility screen**	X							
**Informed consent**	X							
**Allocation**		X						
**INTERVENTION**								
**Modeling virtual 3D-model (according to randomized arm)**			X					
**Printing 3D-model (according to randomized arm)**			X					
**Preoperative information according to randomized arm**				X				
**Surgery**						X		
**ASSESSMENTS**								
**HLSEU-Q16 questionnaire (health literacy)**		X					X	
**Questionnaire (knowledge of disease and planned surgery)**		X			X			X
**Questionnaire of satisfaction**								X
**Semi-directive interview**		X			X			X

(R) : recherche
(S) : soins courants
* 15 jours devant être pris en compte pour l'impression et la transmission du modèle 3D imprimé, l'imagerie doit avoir été réalisée au moins 15 jours avant la consultation d'information.

TEMPORAL SCHEME FOR PATIENT FOLLOW UP

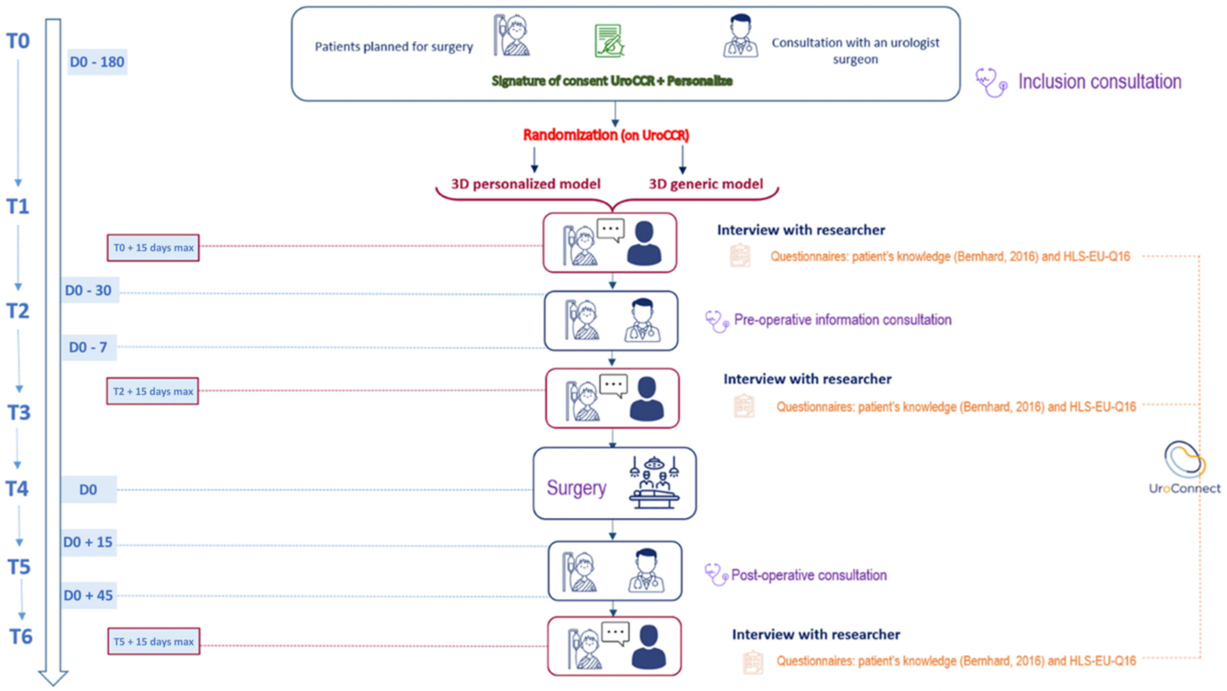

Professionnels

	Presentation of the study to the entire team - T0		Interview with professional
	When setting up the study	Between implementation and 1 year after first inclusion	1 year after the first patient inclusion
Information	✓		
Collection of consent (R)		✓	
Semi-directed interview conducted by a researcher in SHS (R)			✓

7.3 INCLUSION VISIT (T0)

7.3.1 COLLECTION OF CONSENT

During the inclusion visit, the investigating physician informs the participant and answers all their questions regarding the objective, the nature of the constraints, the foreseeable risks and the expected benefits of the research. He also specifies the participant's rights in the context of research and checks the eligibility criteria.

A copy of each information note (Rein-3D PERSONALIZE and UroCCR) and the consent form is then given to the participant by the investigating physician. After this information session, the participant

has a period of reflection. If the participant agrees to participate, the latter and the investigator write their first and last names in clear text, date and sign the two consent forms in two original copies (for participation in the Rein-3D PERSONALIZE project and for collecting the data necessary for analysis in the UroCCR database). These must be signed before any clinical or paraclinical examination required by the research is carried out. The different copies of the information note and the consent form are then distributed as follows:

- An original copy of each information note and each signed consent is given to the participant.

- The other original copies are kept by the investigator (even if the participant moves during the research) in a safe place inaccessible to third parties.

7.3.2 VISIT DETAIL

Patients

The inclusion visit is carried out by the investigator. Before any examination related to the research, the investigator obtains the free and informed consent of the patient (or their legal representative if applicable).

The patients included are received in the urology department, as part of the management of their pathology. The inclusion visit is carried out by the investigator. A member of the SHS research team may participate in the visit as an observer.

Professionals

A collective meeting to present the study is held one month before the start of the interviews by the investigator and the SHS research team who present the study during formal or informal times set up in the department with the professionals.

The SHS research team will act under the responsibility of the investigator.

The SHS researchers inform and answer all questions concerning the objective, the nature of the constraints, the foreseeable risks and the expected benefits of the research. They also specify the possibility for the participant to withdraw from the research at any time, and verify the eligibility criteria. A copy of the information note (with the contact details of the SHS research team and the clinical research associate of the Rein 3D Print PERSONALIZE project) and the consent form is then given to the professional by the research team. The research team then collects the consent to participate.

- An original copy of the information note and the signed consent is given to the participant.

- The other original copy will be kept by the investigator (even if the participant moves during the research) in a safe place inaccessible to third parties.

7.3.3 VISIT/RANDOMIZATION APPROACH

Patient randomization will be performed immediately after the inclusion consultation. It will be done directly on the UroCCR database web interface.

When an investigator wishes to perform randomization, after prior verification of the participant's eligibility, he will connect with his codes on the website: http:uroccr.fr. He will complete the "Research / Kidney-3D PERSONALIZE" tab and confirm all the patient's eligibility criteria. After validation of the content, inclusion and randomization will be performed. The interface will

immediately communicate to the investigator the patient's unique number in the research and the result of the randomization. The result of the randomization will only be communicated to the patient at the Exchange and Information Consultation (T2).

7.4 FOLLOW-UP VISITS

7.4.1 PREOPERATIVE PERIOD (T1)

Patients are seen, between the inclusion visit and the information consultation, by a member of the SHS research team at the hospital or at home according to their preferences at T0 +15 days at the latest. This exchange time corresponds to a face-to-face interview during which the following will be discussed:

- Collection of the patient's experience since the first symptoms leading to the visit to the urology department,
- Collection of socio-demographic data,
- Collection concerning exchanges with those around them about the disease,
- HLSEU-Q16 literacy questionnaire collected via a tablet on UroConnect,
- Questionnaire on understanding renal anatomy and surgical issues via a tablet on UroConnect.

7.4.2 EXCHANGE AND INFORMATION CONSULTATION (T2)

Patients are seen between 30 days and 7 days before the operation by a member of the surgical team. The purpose of this discussion time is to allow the patient to express their questions, share their doubts or misunderstandings in relation to their medical situation and the scheduled intervention. It may lead to explanations on anatomy and simple surgical strategies by the member of the surgical team participating in this interview.

The two interventional groups (Personalized 3D Printed Model Group and Generic 3D Printed Model Group) are presented with the model as a discussion support.

The explanations given to patients are not standardized within the framework of the study in order to be closer to usual practice.

The duration of this visit as well as the presence or absence of an accompanying person will be collected.

A member of the SHS research team may participate in the visit as an observer.

7.4.3 SECOND PREOPERATIVE PERIOD (T3)

Patients are seen between the information consultation and the operation by a member of the SHS research team at the hospital or at home according to their preferences at the latest at T2 +15 days. This exchange time corresponds to a face-to-face interview during which the following elements will be discussed:

- Collection of the patient's experience since the last interview,
- Collection concerning exchanges with those around them about the disease,
- Questionnaire for understanding renal anatomy and surgical issues with a tablet on UroConnect.

7.4.4 SURGERY (T4)

No specificity for the study but, as for any patient included in UroCCR, the characteristics and technical progress of the surgical procedure will be collected. There will be, due to the study, no modification of the surgical technique of robot-assisted partial nephrectomy as envisaged by the surgeon.

7.4.5 POSTOPERATIVE VISIT (T5)

The post-operative assessment will be collected. There will be, due to the study, no modification of the post-operative consultation other than the potential presence of a member of the SHS research team during the consultation.

7.5 END OF RESEARCH VISIT (T6)

Patients

The research data collection ends no later than 15 days after the last postoperative consultation.

Patients will be seen by a member of the SHS research team at the hospital or at home, according to their preferences. This exchange time corresponds to a face-to-face interview during which the following elements will be discussed:

- Collection of the patient's experience since the last interview,
- Collection concerning exchanges with those around them about the disease,
- HLSEU-Q16 literacy questionnaire via a tablet on UroConnect,
- Questionnaire on understanding renal anatomy and surgical issues via a tablet on UroConnect,
- Satisfaction questionnaire via a tablet on UroConnect.

Professionals

An interview to collect the professional's experience following the implementation of 3D Print tools in their relationship with patients and their entourage will be carried out within the urology department no later than one year after the inclusion of the first patient.

7.6 STOPPING RULES

7.6.1 TERMINATION OF A PERSON'S PARTICIPATION IN RESEARCH

A participant who wishes to abandon or withdraw his or her consent to participate in the research (as he or she is entitled to do at any time) is no longer followed within the framework of the protocol, but must be subject to the best possible care taking into account his or her state of health and the current state of knowledge.

A withdrawal is a decision by an included participant to assert his or her right to interrupt his or her participation in a research study, at any time during the follow-up, without incurring any prejudice as a result and without having to justify himself or herself.

A withdrawal of consent is a decision by a participant to reconsider his or her decision to participate in a research study and to assert his or her right to cancel his or her informed consent, at any time during the follow-up and without incurring any prejudice as a result and without having to justify himself or herself.

The investigator should identify the cause of the dropout/withdrawal and assess whether it is possible to collect the primary endpoint variable at the time of dropout/withdrawal. Dropouts/withdrawals should be promptly notified to the coordinating investigator, sponsor, and methodology and data management center. The reasons and date of dropouts should be documented in the case report and in the participant's medical record.

7.6.2 STOP RESEARCH

End of research or planned termination of research: end of participation of the last person who participates in the research, also called the last visit of the last participant included in the research.

When the research has reached its planned end (planned termination), the end of the research must be declared to the ANSM and the CPP within 90 days.

Early termination of research: clinical research is terminated (definitively) early. This is the case, in particular, when the sponsor decides:

- not to start the research despite obtaining a favorable opinion from a CPP;

- not to resume the research after having temporarily interrupted it or after its suspension by the ANSM.

When the research is terminated (definitively) early, the end of the research must be declared to the ANSM within 15 days, indicating the reasons for this termination. Temporary cessation of research: the temporary cessation of clinical research consists of:

- stopping the inclusion of new people in this research;

- and/or stopping the practice of the acts provided for in the research protocol.

Any decision by the sponsor to temporarily interrupt the research must be the subject of immediate information to the ANSM and the CPP concerned and, in a second stage and within a maximum period of 15 calendar days following the date of this interruption, of a request for authorization of substantial modification concerning this temporary cessation submitted to the ANSM and a request for advice to the CPP concerned.

7.7 DEVIATIONS FROM PROTOCOL

Deviations can concern all aspects of a research protocol: inclusion process, monitoring, measurement of judgment criteria, treatments. All must be documented by the investigator and discussed in the Scientific Council.

Only dropouts result in a cessation of monitoring. Even in the event of a deviation from the protocol, the participant's monitoring must be carried out until the end of the period provided for in the protocol.

7.7.1 PREMATURE AND DEFINITIVE TERMINATION OF THE RESEARCH PROCEDURE

Participants who stop prematurely continue to be monitored as planned by the protocol. Under no circumstances should the planned monitoring be modified.

The participant must receive the best possible care given his or her state of health and the current state of knowledge.

7.7.2 LOST TO FOLLOW UP

A participant is considered lost to follow-up when he or she stops the follow-up planned under the protocol for no reason known to the investigator, so that data collection cannot be carried out as planned.

Participants lost to follow-up should be actively sought by the investigator.

7.7.3 PARTICIPANT WRONGLY INCLUDED

A participant is considered to be wrongly included when he or she has actually been included in the research while not meeting all the eligibility criteria. Participants wrongly included must be discussed by the Scientific Council. They must continue to be followed as provided for in the protocol until a decision is made by the Scientific Council.

7.8 SIMULTANEOUS PARTICIPATION IN OTHER RESEARCH, EXCLUSION PERIOD, COMPENSATION AND REGISTRATION IN THE VRB FILE

Outside of the ACCURATE and ANXIETY studies, the person may participate simultaneously in another research.

No compensation will be received by the participants during the duration of this research.

8. MANAGEMENT OF ADVERSE EVENTS / SIDE EFFECTS / INCIDENTS

Adverse events/adverse effects/incidents must be reported to the various health monitoring circuits applicable to each product or practice concerned (care vigilance, pharmacovigilance, haemovigilance, cosmetovigilance, etc.) in accordance with the regulations in force.
Reporters must specify that the patient is included in a clinical trial and precisely identify the clinical trial concerned.
If the investigator becomes aware of a breach of patient safety in the context of the research, he must inform the sponsor without delay.

9. STATISTICAL APPROACH

9.1 STUDY SIZE

Patients

The study approach is mainly of an anthropological type by systemic essence, it is based on a mixed approach with observations, interviews and questionnaires. In order to be able to combine, within the framework of the collection and then the analysis, the qualitative and quantitative approaches it was decided to build a study with 60 patients divided into 2 randomized groups of 30 patients. Patients who will have unusable data will be excluded from the analyses and replaced with a maximum total inclusion of 68 patients.

Professionals

Similarly it was chosen to conduct interviews with 30 professionals, meeting the inclusion criteria, and making it possible to cover the heterogeneity in terms of profession and seniority. This will make it

possible to conduct the interviews correctly in the allotted time and to have a representation within the cohort of professional heterogeneity and experience.

9.2 STATISTICAL METHODS USED

9.2.1 ANALYSIS STRATEGY

The data will be analyzed by the SHS research team to ensure cross-analysis of qualitative and quantitative data.
The main analysis will be carried out by sub-treatment for patients, i.e. the data of all participants must be analyzed with regard to the 3D printed model actually administered, even if randomization has decided otherwise, on available data.

The diagram below presents the different phases of the mixed method research proposed here. Many collection tools are used in this study to be able to study the effects of using a personalized 3D printed kidney model versus a generic 3D printed kidney model as a mediation tool on the patient experience and their interactions with professionals. This triangulation approach (21) requires specific analyses of the data obtained from the different collection tools, associated with cross-interpretations to meet the different objectives.

A descriptive analysis will be performed globally and by 3D model group.

A comparison analysis between the randomization groups will be performed, systematically without adjustment and with adjustment on the initial prognostic factors whose distribution could be, despite randomization, unbalanced.

9.2.2 PATIENTS INCLUDED IN THE ANALYSIS

Only patients and professionals who present at least one of the following conditions may be excluded from the analysis:

- Patients wrongly included due to unsigned consent;
- Patients or professionals wrongly included due to major eligibility criterion(s) not met;
- Patients or professionals who have withdrawn their consent.

This exclusion decision will be taken by the Scientific Council without the knowledge of the randomization group and the patient's progress after inclusion.

Apart from these exclusions, patients or professionals who have died, been lost to follow-up or have abandoned the research will all be included in the analysis.

9.2.3 RISK OF THE FIRST KIND

Statistical data comparison analyses will be performed at an overall risk of error of = 5%.

9.2.4 DESCRIPTIVE STATISTICAL METHOD

For the results of the questionnaires used:

- The number and percentage of participants (patients and professionals) with missing data will be described for each variable of interest. The reason for the missing data will be documented as much as possible in order to interpret the results.
- Qualitative variables will be described in terms of numbers, percentages and 95% confidence intervals according to the exact binomial distribution.
- Quantitative variables will be described in terms of numbers, means, standard deviations, medians, ranges and interquartile ranges.

For observations of consultations, systematically collected data will be described (duration of the different visits, delays between visits, characteristics of the professional(s) ...).

An analysis of the interviews will lead to a statistical approach of content, allowing to describe, among other things, the elements of understanding.

9.2.5 COMPARATIVE STATISTICAL METHOD

The distributions of qualitative variables will be compared between groups by $\chi 2$, or corrected $\chi 2$, or Fisher exact tests, depending on the values of the expected numbers under the assumption of independence.

The distributions of quantitative variables will be compared between groups by the Student test if the conditions of validity of the test are respected (normal distribution, homogeneous variances). If the variances are unequal between the two groups, a Student test for unequal variances will be used and if the distribution is not normal, a Wilcoxon test will be used.

9.2.6 QUALITATIVE DATA STATISTICAL METHOD

The interviews and the observation collections are sources of important content to be analyzed in order to study the themes addressed, the resemblance and heterogeneity between respondents, but also the evolution of the latter during the different phases of the study, while taking into account for the samples of patients the belonging to one or the other of the groups to see the differences.

9.2.7 STATISTICAL SOFTWARE

The analyses will be carried out with R software (version 4.2.3 or later) and Nvivo software (version 9.4 or later).

9.3 ANALISIS PLAN

9.3.1 DESCRIPTION OF INCLUSIONS, DEVIATIONS AND FOLLOW-UP

The following elements will be presented:

- Verification of eligibility criteria,
- The study flow diagram following CONSORT recommendations,
- A description of the causes of death, abandonment, patients and professionals lost to follow-up or having abandoned the research, patients who were not operated on,
- Deviations from the protocol (particularly concerning deadlines),
- Follow-up visits carried out: number of patients having carried outeach of the follow-up visits.

9.3.2 CHARACTERISTICS OF PATIENTS AT INCLUSION

Patients will be described according to the following variables:

- demographic characteristics,

- clinical characteristics, - biological characteristics,

- strategy characteristics.

9.3.3 ANALYSIS OF THE MAIN OBJECTIVE

The main objective is to study the effects of using a personalized 3D printed kidney model versus a generic 3D printed kidney model as a mediation tool, throughout the pathway, on the patient experience and their interactions with professionals, before and after partial nephrectomy.

The analysis of the main objective will be carried out by relying on the cross-referencing of elements from the different collection tools and will depend on the content of the interviews as well as observations. The analysis of the verbatims will be based on observational data and data from the questionnaires. A triangulation analysis of the data will therefore be carried out globally and then by group to study the differences.

A descriptive analysis will be carried out globally and by strategy group for patients on all data from the questionnaires, socio-demographic data as well as data characterizing the tumor. The description of the socio-demographic data of each of the two groups of patients (generic 3D model group / specific 3D model group) will be carried out: by indicating the frequencies (e.g.: sex, profession, etc.), the means and standard deviations (e.g.: age, illness delay, etc.) allowing the distribution of the two groups to be compared.

A comparison analysis between the groups of 3D model types will be carried out, systematically on the data from the questionnaires and the socio-demographic data.

The data from the interview transcripts will be analyzed in order to be able to study the unique life experiences of the patients as well as the common elements between patients and the heterogeneities between groups.

Observations and interviews are collection methods that do not automatically lead to a statistical analysis of the data. The statistical analysis will only cover part of the data collected, qualitative and quantitative.

The description of the patients' experience will be carried out based on the analysis of the contents of the interviews in a transversal manner for each time but also according to a longitudinal analysis taking into consideration the 3 times (T, T3 and T6) and specifying the group to which the patient belongs.

9.3.4 ANALYSIS OF SECONDARY OBJECTIVES

The description of interactions between professionals and patients, specifying the terms used and their frequencies, will be carried out via the analysis and synthesis of the information collected via the observation notebooks. This analysis will make it possible to respond to secondary objectives 1 and 2. Additional elements concerning patient and entourage interactions may come from interviews with patients and will enrich the response to objective 2.

To respond to the third secondary objective, a synthesis of all the information collected as part of the observations but also from interviews with patients and professionals will make it possible to trace the use made of 3D models and to understand the users and the circumstances of use.

The evolution of the understanding of the disease and surgery will be studied by analyzing the content of the interviews and the questionnaire on understanding renal anatomy and surgical issues with a tablet on UroConnect. The content will be compared between the two groups.

A descriptive analysis of the distribution of scores obtained by patients for the HLS-EU16 literacy questionnaire will be carried out in each group for times 1 and 6. A comparison analysis between time 1 and time 6 will be carried out, for each group, by a paired Student test, after checking the homogeneity of variances, otherwise non-parametric tests will be used. Finally, the changes between the two groups (HLSEU-Q16 at time T6 - HLSEU-Q16 at time T1) will be compared by a Student test.

A second analysis will be carried out using the classification of the literacy score into three levels as indicated in section 4.2, the data will be described for each time and then a comparison between times will be carried out by a paired Chi-square test.

A thematic content analysis of the interviews conducted with professionals will be carried out.

10. RESEARCH GUIDANCE

10.1 SCIENTIFIC COMITEE

10.1.1 COMPOSITION

It is composed of the following people: Dr Gaëlle MARGUE (President), Pr Jean-Christophe BERNHARD (Scientific Manager), Pr Laura RICHERT (Methodologist), Roxane COUËRON (Biostatistician), Marthe-Aline JUTAND, (Researcher in educational sciences), Héléne HOARAU (Anthropologist), Sarah MASANET (PhD student in educational and training sciences), Solène RICARD (Project Manager), Manon JAFFREDO (Project Manager), Clémence MORICE (Coordinating Clinical Research Associate) and the representative of the promoter.

10.1.2 RYTHME DES REUNIONS

The Scientific Research Council meets according to the needs of the study and at least once a year.

10.1.3 ROLE

- Its mission is to make any important decision at the request of the coordinating investigator concerning the proper conduct of the research and compliance with the protocol.
- It verifies compliance with ethics.
- It obtains information from the Methodology and Data Management Center and the coordinating investigator center of the research on the progress of the research, any problems and the available results.

- It decides on any relevant modification of the protocol necessary for the continuation of the research, in particular:

• measures to facilitate recruitment in the research,

• modifications to the research documents (protocol and information and consent collection documents) before their presentation to the CPP,

• measures that ensure the best security for people participating in the research, • discussion of the results and the strategy for publishing these results.

- The Scientific Council may propose to extend or interrupt the research in the event of too slow an inclusion rate, too many lost to follow-up, major violations of the protocol or for medical and/or administrative reasons. It specifies the possible modalities of the extended monitoring of the participants included in the research. - At the end of the meeting, the chair of the Scientific Council must inform the sponsor of the decisions taken. Decisions concerning a major modification or a budget modification must be approved by the sponsor.

10.2 INDEPENDANT SURVEILLANCE COMITEE

This study does not require the establishment of an independent monitoring committee due to the absence of any treatment that could lead to premature termination of the research and the nature of the procedures under study not entailing any significant risk for the patients.

11. MANAGEMENT AND PROCESSING OF SOURCE DATA AND DOCUMENTS

11.1 DONNEES ET DOCUMENTS SOURCES

Source data is all information contained in original documents, or in authenticated copies of these documents, relating to clinical examinations, observations or other activities carried out in the context of research and necessary for the reconstruction and evaluation of the research. The documents in which the source data are recorded are called source documents.

Need for UroCCR data

• Socio-demographic characteristics of the patient (age, sex),
• Tumor size,
• Tumor complexity score – RENAL,
• History of family cancer,
• Diagnosis of kidney cancer,
• Distance hospital home,
• Duration of the operation,
• Surgical history with general anesthesia, • Presence of an accompanying person during the consultation.

11.2 DATA COLLECTION INSTRUCTIONS

All information required by the protocol must be recorded in the medical record. Data must be collected as they are obtained and transcribed in a clear and legible manner. Medical data will be collected in the UroCCR database..

11.3 DATA MANAGEMENT AND CIRCUIT

11.3.1 DATA MANAGEMENT SOFTWARE

11.3.1.1 SOFTWARE

eCRF for quantitative data: the software used for data management is an eCRF accessible at the following address: https://uroccr.fr

The IT maintenance and development of the eCRF are managed by CREDIM (Centre de Recherche et Développement en Informatique Médicale) which is an IT platform created within the University of Bordeaux.

UroConnect: The maintenance and development of the UroConnect application are provided by the company Resilience based in France (https://www.resilience.care/).

3D modeling: The software used for 3D modeling is Synapse 3D from Fujifilm.

Nvivo Pro for qualitative data: The data will be transcribed into a word processing document (.docx type) or directly within the qualitative analysis support software Nvivo Pro 13®, then analyzed via the Nvivo Pro 13® software.

11.3.1.2 DATA HOSTING

CREDIM: The database management system used is Microsoft SQL server.

UroConnect: The data collected by UroConnect is stored in a service hosted in France (provider Ecritel).

3D modeling: The 3D models are hosted on the servers or on the stand-alone computer with the Synapse 3D software, on the Bordeaux University Hospital server (NextCloud) then on the computer of the TechnoShop technician in charge of carrying out the 3D printing.

Qualitative data entry software:

The following documents relating to this research are archived in accordance with Good Clinical Practices and current regulations:

• The protocol and any modifications to the protocol: by the sponsor and the SHS research team,

• All other documents and letters relating to the research by the sponsor: the SHS research team.

• The notebooks: by the SHS research team within the CeDS laboratory.

• The other source data and transcribed data will be kept on the computers of the SHS research team for up to 5 years after the end of the ANR-21-RHUS-0015 project, i.e. 2032.

11.3.1.3 DATA SECURITY

UroConnect: the data collected by UroConnect are stored in an HDS environment. eCRF for quantitative data: the server is located in a dedicated room, without windows. Entry to the secure room is by badge. The department doors are secured and locked in the evening. No computers are freely accessible, domain authentication is mandatory.

The management of access rights to the data collected by the eCRF is managed by CREDIM. Only the database managers, the project team and the auditors have direct access rights to the database.

The USMR will send CREDIM the list of people who must have access to the data. Statisticians and DMs will therefore have read-only access to the data.

Qualitative data (anonymized): all anonymized data (in audio or text or Nvivo format) will be stored on an external disk located at the CeDS, locked away for the duration of the study. The data will then be stored in archives on the University of Bordeaux network (CIRRUS system).

11.3.2 DATA ENTRY

UroCCR: Data entry is the responsibility of the center investigator in the eCRF. Any person other than the investigator performing the entry in the eCRF must be previously trained and delegated by the investigator to do so.

The questionnaires will be collected either on paper or directly via the UroCONNECT digital tool which will distribute, at an appropriate time, the various questionnaires defined by the protocol by automatic sending to patients.

The source data from observations (note-taking in a notebook) and interviews (digital audio support) will be transcribed, entered, coded and encoded exclusively by the SHS research team.

Thus, the data will be transcribed into a word processing document (of the .docx type) or directly within the Nvivo Pro 13® qualitative analysis support software, then analyzed via the Nvivo Pro 13® software.

11.3.3 DATA ENCODING

Prescribed treatments and clinical events are coded in the eCRF in order to perform data control and analysis.

The following dictionaries are used for coding medical terms:

- MedDRA (current version) FR/US,
- ATC version.

The UroCCR investigation team is in charge of data coding, under the responsibility of the investigator.

11.3.4 DATA CONTROL

Checks are scheduled to verify the consistency and completeness of the data entered in the eCRF. The list of checks to be implemented is defined jointly between the coordinating investigator and the USMR, in the study data validation plan.

The UroCCR MD and the coordinating ARC are responsible for managing correction requests, which they launch regularly.

The investigator makes the necessary corrections to resolve the correction requests.

11.3.5 DATA TRANSFER

Data transfers (sending, receiving) are carried out in accordance with the procedure in force at the USMR. The data transfer procedures must be defined in the Data Management Plan. For security reasons, data files are pseudonymised and then transferred via the secure CIRRUS or NextCloud platform. As part of this protocol:

• Data may be transferred to the Bordeaux University Hospital server (administered by the Bordeaux University Hospital DSIN) for the performance of tasks by the CeDS.

• A data transfer is also planned to the CeDS laboratory (University of Bordeaux) for additional analyses of qualitative data.

Other data transfers may be requested and approved according to the procedure in force at the USMR.

11.4 DATA CONFIDENTIALITY

In accordance with the legislative provisions in force, persons with direct access to the source data will take all necessary precautions to ensure the confidentiality of information relating to experimental drugs, research, the persons participating in it, and in particular with regard to their identity and the results obtained. These persons, like the investigators themselves, are subject to professional secrecy.

During the research or at its conclusion, the data collected on the persons participating in it and transmitted to the sponsor by the investigators (or any other specialized stakeholders) will be pseudonymized. They must under no circumstances clearly show the names of the persons concerned or their addresses.

Each participant will be assigned a confidential identification code consisting of a participant number (3 digits).

The sponsor will ensure that each person participating in the research has given their written consent for access to the individual data concerning them and strictly necessary for the quality control of the research.

11.5 RETENTION OF DOCUMENTS AND DATA RELATING TO RESEARCH

The following documents relating to this research are archived by the investigator in accordance with Good Clinical Practice, the decree of August 11, 2008 sets the retention period for documents relating to health research and the European regulation on medicines:

- for a period of 20 years following the end of the research (research not relating to a product mentioned in Article L.5311-1 of the Public Health Code),

• The protocol and any modifications to the protocol

• The observation notebooks (paper or electronic copies)

• The source files of participants who have signed consent

• All other documents and correspondence relating to the research

- for a period of 30 years following the end of the research,

• The original copy of the informed consents signed by the participants

All these documents are under the responsibility of the investigator during the regulatory archiving period.

No movement or destruction may be carried out without the sponsor's agreement. At the end of the regulatory archiving period, the sponsor will be consulted for destruction. All data, documents and reports may be subject to audit or inspection.

The data collected for the study will be accessible to persons authorized by the Bordeaux University Hospital, for two years after the last publication of the research results. They will be archived for 20 years after the end of the study in accordance with the regulations in force.

11.6 RANSFER OF DATA

Data management is provided by the CeDS. The conditions for the transfer of all or part of the research database are decided by the research promoter and are the subject of a written contract.

12. CONTROL AND QUALITY ASSESSMENT

12.1 DATA ACCESS

Acceptance of participation in the protocol implies that the investigators will make available the documents and individual data strictly necessary for monitoring, quality control and auditing of the research, to persons having access to these documents in accordance with the legislative and regulatory provisions in force.

12.2 QUALITY CONTROL

Quality control will be carried out by a clinical research associate mandated by the sponsor in accordance with the risk-based monitoring plan (logistics, impact, resources) defined for the research.

The latter defines the nature of the elements to be verified, the modalities and the frequency of visits to the investigation center.

Each visit will be the subject of a monitoring report in a written report sent to the principal investigator of the center.

12.3 AUDIT ET INSPECTION

An audit may be carried out at any time by persons mandated by the sponsor and independent of the persons conducting the research. Its objective is to verify the safety of participants and respect for their rights, compliance with applicable regulations and the reliability of the data.
An inspection may also be carried out by a competent authority (ANSM for France or another regulatory authority in the context of European research for example).
The audit, as well as the inspection, may apply to all stages of the research, from the development of the protocol to the publication of the results and the classification of the data used or produced in the context of the research.
The investigators agree to comply with the sponsor's requirements regarding an audit and with the competent authority for an inspection of the research.

13. ETHICAL AND REGLEMENTARY CONSIDERATIONS

13.1 COMPLIANCE WITH REFERENCE TEXTS

The sponsor and the investigator(s) undertake to ensure that this research is carried out in accordance with Law No. 2012-300 of March 5, 2012 relating to research involving human beings, as well as in accordance

with Good Clinical Practices (I.C.H. E6 (R2) of December 1, 2016 and decision of November 24, 2006) and the Declaration of Helsinki (which can be found in its full version on the website www.wma.net).

The research is conducted in accordance with this protocol. Except in emergency situations requiring the implementation of specific therapeutic procedures, the investigator(s) undertake to respect the protocol in all respects.

This research has received the favorable opinion of the Committee for the Protection of Persons (CPP) Sud Est IV and has been the subject of information with the ANSM. The Bordeaux University Hospital, the promoter of this research, has taken out a civil liability insurance contract with Lloyd's Insurance Company SA (represented by BEAH, agent) in accordance with the provisions of the Public Health Code.

The data required for this research are recorded in the UroCCR database, which obtained authorization from the National Commission for Information Technology and Civil Liberties (CNIL) on 12/04/2013 (authorization request no. 912578, decision DR-2013-206). The data in the UroCCR database are subject to computerized processing at CREDIM in accordance with the provisions of the law relating to information technology, files and freedoms (law n° 78-17 of 6 January 1978 relating to information technology, files and freedoms amended by law n° 2018-493 of 20 June 2018 relating to the protection of personal data) and the general data protection regulation (EU regulation 2016/679).

This research falls within the framework of the "Reference Methodology" MR-001 in application of the provisions of article 54 of the law of 6 January 1978 amended relating to information, files and freedoms. The Bordeaux University Hospital, the USMR of the Bordeaux University Hospital have signed a commitment to comply with this "Reference Methodology".

This research is registered in the ID-RCB database under number 2024-A00129-38. This research is registered on the site http://clinicaltrials.gov/

13.2 PROTOCOL MODIFICATIONS

Any substantial modification, i.e. any modification likely to have a significant impact on the protection of individuals, on the conditions of validity and on the results of the research, on the quality and safety of the products tested, on the interpretation of the scientific documents that support the conduct of the research or on the methods of conducting it, is the subject of a written amendment that is submitted to the sponsor; the latter must obtain, prior to its implementation, a favorable opinion from the CPP.

Non-substantial modifications, i.e. those that do not have a significant impact on any aspect of the research whatsoever, are communicated to the CPP for information purposes.

All modifications are validated by the sponsor, and by all research stakeholders concerned by the modification, before submission to the CPP. This validation may require the meeting of any committee set up for the research.

All modifications to the protocol must be brought to the attention of all investigators participating in the research. The investigators undertake to respect its content. Any modification that alters the care of participants or the benefits, risks and constraints of the research is the subject of a new information note and a new consent form, the collection of which follows the same procedure as that mentioned above.

14. FINAL REPORT

Within one year of the end of the research or its interruption, a final report will be drawn up and signed by the sponsor and the investigator. This report will be made available to the competent authority. The sponsor will transmit to the CPP and, where applicable, to the ANSM the results of the research in the form of a summary of the final report within one year of the end of the research.

15. RULES RELATING TO PUBLICATION

15.1 SCIENTIFIC COMMUNICATIONS

The final report is edited by the CeDS with the contribution of expertise from the USMR on methodological issues around quantitative data. This analysis gives rise to a written report that is submitted to the sponsor, who will forward it to the Committee for the Protection of Persons and to the competent authority.

Any written or oral communication of the results of the research must receive the prior agreement of the coordinating investigator and, where applicable, of any committee set up for the research.

The coordinating investigator undertakes to make the results of the research available to the public, whether negative and inconclusive or positive. The publication of the main results mentions the CHU de Bordeaux, all the investigators who included or followed participants in the research, methodologists, biostatisticians and data managers who participated in the research, members of the committee(s) set up for the research and the mention "this work has benefited from State aid managed by the National Agency for Research under the third PIA integrated into France 2030 bearing the reference ANR-21-RHUS-0015". For publications in SHS, these rules apply to acknowledgements with the mention "this work has benefited from State aid managed by the National Agency for Research under the third PIA integrated into France 2030 bearing the reference ANR-21-RHUS-0015". The international rules for writing and publication (The Uniform Requirements for Manuscripts of the ICMJE, April 2010) will be taken into account concerning the quality of authors.

15.2 COMMUNICATION OF RESULTS TO PARTICIPANTS

In accordance with Law No. 2002-303 of March 4, 2002, participants are informed, upon request, of the overall results of the research.

BIBLIOGRAPHY

1. Bernhard JC, Isotani S, Matsugasumi T, Duddalwar V, Hung AJ, Suer E, et al. Personalized 3D printed model of kidney and tumor anatomy: a useful tool for patient education. World J Urol. mars 2016;34(3):337-45.

2. Travaline JM, Ruchinskas R, D'Alonzo GE. Patient-physician communication: why and how. J Am Osteopath Assoc. janv 2005;105(1):13-8.

3. Curchod C. 1 - Identifier les facteurs de détérioration de la relation. In: Curchod C, éditeur. Relations soignants–soignés [Internet]. Paris: Elsevier Masson; 2009 [cité 3 août 2023]. p. 13-28. Disponible sur: https://www.sciencedirect.com/science/article/pii/B9782294705373500017

4. Rey S, Leduc A, Debussche X, Rigal L, Ringa V. Une personne sur dix éprouve des difficultés de compréhension de l'information médicale. Etudes Résultats. 2023;1269:8.

5. Stephanie CJ, Mathieu A, Aurore M, Monique MRT. Outpatients' perception of their preoperative information regarding their health literacy skills and their preoperative anxiety level: Protocol for a prospective multicenter cross-sectional study. Medicine (Baltimore). 21 mai 2021;100(20):e26018.

6. Köhler H, Dorozhkina R, Gruner-Labitzke K, de Zwaan M. Specific Health Knowledge and Health Literacy of Patients before and after Bariatric Surgery: A Cross-Sectional Study. Obes Facts. 2020;13(2):166-78.

7. Hälleberg Nyman M, Nilsson U, Dahlberg K, Jaensson M. Association Between Functional Health Literacy and Postoperative Recovery, Health Care Contacts, and Health-Related Quality of Life Among Patients Undergoing Day Surgery. JAMA Surg. août 2018;153(8):738-45.

8. Shen HN, Lin CC, Hoffmann T, Tsai CY, Hou WH, Kuo KN. The relationship between health literacy and perceived shared decision making in patients with breast cancer. Patient Educ Couns. 1 févr 2019;102(2):360-6.

9. Pieterse AH, Gulbrandsen P, Ofstad EH, Menichetti J. What does shared decision making ask from doctors? Uncovering suppressed qualities that could improve person-centered care. Patient Educ Couns. 1 sept 2023;114:107801.

10. Luckenbaugh AN, Moses KA. The impact of health literacy on urologic oncology care. Urol Oncol. avr 2022;40(4):117-9.

11. Marche H. Au-delà de l'autonomie du patient : l' « esprit de soin » dans les trajectoires de cancer avancé. Anthropol Santé Rev Int Francoph Anthropol Santé [Internet]. 11 mai 2015 [cité 11 mai 2023];(10). Disponible sur: https://journals.openedition.org/anthropologiesante/1595

12. Canfell OJ, Meshkat Y, Kodiyattu Z, Engstrom T, Chan W, Mifsud J, et al. Understanding the Digital Disruption of Health Care: An Ethnographic Study of Real-Time Multidisciplinary Clinical Behavior in a New Digital Hospital. Appl Clin Inform. 9 nov 2022;13(5):1079-91.

13. Scott ER, Singh A, Quinn A, Boyd K, Lallas CD. How I Do It: Cost-effective 3D printed models for renal masses. Can J Urol. oct 2021;28(5):10874-7.

14. Sørensen K, Van den Broucke S, Pelikan JM, Fullam J, Doyle G, Slonska Z, et al. Measuring health literacy in populations: illuminating the design and development process of the European Health Literacy Survey Questionnaire (HLS-EU-Q). BMC Public Health. 10 oct 2013;13(1):948.
15. Rouquette A, Nadot T, Labitrie P, Van den Broucke S, Mancini J, Rigal L, et al. Validity and measurement invariance across sex, age, and education level of the French short versions of the European Health Literacy Survey Questionnaire. PloS One. 2018;13(12):e0208091.
16. Kaufmann JC. Introduction. In: L'entretien compréhensif [Internet]. Paris: Armand Colin; 2016 [cité 15 nov 2022]. p. 9-11. (128; vol. 4e éd.). Disponible sur: https://www.cairn.info/l-entretiencomprehensif--9782200613976-p-9.htm
17. Fainzang S. La relation médecins-malades: information et mensonge. (France): Paris : PUF; 2006. 159 p. (Ethnologies).
18. Pierron JP. Une nouvelle figure du patient ? Les transformations contemporaines de la relation de soins. Sci Soc Santé. 2007;25(2):43-66.
19. Renault L. L'analyse qualitative entre disciplines. Polit Soc. 2020;1-2(1):43-53.
20. Morse JM. Approaches to Qualitative-Quantitative Methodological Triangulation. Nurs Res. avr 1991;40(2):120.
21. Balard F, Kivits J, Schrecker C, Volery. L'analyse qualitative en santé. In: Les recherches qualitatives en santé. Malakoff: Armand Colin; 2016. (Collection U).

ANNEXES

Appendix 1: HLS-EU16 Literacy SCALE

HLS-EU16 version Française (Version 2020)

Indiquez, sur une échelle de très facile à très difficile, dans quelle mesure il est facile pour vous de...

	très facile	facile	difficile	très difficile
... trouver des informations sur les traitements des maladies qui vous concernent ?	❑	❑	❑	❑
... savoir où obtenir l'aide d'un professionnel quand vous êtes malade ? (Par ex. médecin, infirmier, pharmacien ou psychologue)	❑	❑	❑	❑
... comprendre ce qu'un médecin vous dit ?	❑	❑	❑	❑
... comprendre les consignes de votre médecin ou pharmacien sur la manière de prendre vos médicaments ?	❑	❑	❑	❑
... savoir quand il serait utile d'avoir l'avis d'un autre médecin ?	❑	❑	❑	❑
... utiliser les informations que le médecin vous donne pour prendre des décisions concernant votre maladie ?	❑	❑	❑	❑
... suivre les consignes de votre médecin ou pharmacien ?	❑	❑	❑	❑
... trouver des informations sur comment faire en cas de problèmes psychologiques ? (Par ex. stress, dépression ou anxiété)	❑	❑	❑	❑
... comprendre les mises en gardes concernant l'impact sur la santé de certains comportements comme fumer, ne pas faire assez d'exercice et boire trop ?	❑	❑	❑	❑

Tournez la page s'il vous plaît...

Indiquez, sur une échelle de très facile à très difficile, dans quelle mesure il est facile pour vous de...

	très facile	facile	difficile	très difficile
... comprendre les informations sur les dépistages et examens recommandés ? (Par ex. dépistage du cancer colorectal, test de glycémie)	❑	❑	❑	❑
... évaluer la fiabilité des informations disponibles dans les médias sur ce qui est dangereux pour la santé ? (Par ex. journaux, télévision ou internet)	❑	❑	❑	❑
... savoir comment vous protéger des maladies à partir des informations disponibles dans les médias ? (Par ex. journaux, télévision ou internet)	❑	❑	❑	❑
... vous renseigner sur les activités bénéfiques pour votre santé et votre bien être ? (Par ex. relaxation, exercice physique, yoga)	❑	❑	❑	❑
... comprendre les conseils de votre famille ou de vos amis en matière de santé ?	❑	❑	❑	❑
... comprendre les informations disponibles dans les médias pour être en meilleure santé ?	❑	❑	❑	❑
... identifier quels sont les comportements de votre vie de tous les jours qui ont un impact sur votre santé ?	❑	❑	❑	❑

Fin du questionnaire.

Short version of the "European Health Literacy Survey Questionnaire" (Sørensen et al., 2013), validated in French by Rouquette in 2018 (Rouquette et al., 2018).
Available on:https://reflis.fr/wp-content/uploads/2020/07/HLSEU16-Francais-2020.pdf

APPENDIX 2 – Bernhard study questionanire (2016)

1/ What I know about the kidney, generally speaking	1/ Ce que je sais sur le rein, de manière générale	True / Vrai	False / Faux	Don't know / je ne sais pas
1.1- The kidney is a paired organ	1.1- Le rein est un organe pair			
1.2- The kidney function is to "clean" my blood	1.2- La fonction du rein est d'épurer le sang			
1.3- The kidney produces urines	1.3- Le rein produit l'urine			
1.4- The urine is collected in cavities inside of the kidney, called the collecting system	1.4- L'urine est recueillie dans des cavités situées à l'intérieur du rein, appelées système collecteur.			
1.5- The kidney is a highly vascularized organ; a lot of blood lows through the renal vessels	1.5- Le rein est un organe très vascularisé, avec un débit sanguin important			
1.6- The renal vessels can be described as an artery and a vein	1.6- Les vaisseaux du rein comprennent l'artère et la veine rénales			
1.7- To work properly the kidney needs blood coming through the renal artery	1.7- Pour fonctionner correctement, le rein a besoin de sang qui arrive par l'artère rénale			
1.8- When the kidneys are not working properly this leads to renal insufficiency	1.8- Lorsque les reins ne fonctionnent pas correctement, cela conduit à l'insuffisance rénale			
2/ What I know about my disease:	2/ Ce que je sais de ma maladie :	True / Vrai	False / Faux	Don't know / je ne sais pas
2.1- My kidney is bearing a tumor	2.1- Mon rein est porteur d'une tumeur			
2.2- The tumor is located in the mid part of my kidney	2.2- La tumeur est située dans la partie médiane de mon rein			
2.3- The tumor is in close contact with the kidney vessels	2.3- La tumeur est en contact étroit avec les vaisseaux du rein			
2.4- The tumor is in close contact with the collecting system	2.4- La tumeur est en contact étroit avec le système collecteur			
3/ What I understand about my planned surgery	3/ Ce que je comprends de l'intervention chirurgicale prévue	True / Vrai	False / Faux	Don't know / je ne sais pas
3.1- My surgeon will try to remove the tumor only	3.1- Mon chirurgien essaiera d'enlever uniquement la tumeur			
3.2- My surgeon will remove the entire kidney	3.2- Mon chirurgien va enlever tout le rein			
In case of tumor only removal my surgeon will have to cut the kidney itself to separate the tumor from surrounding healthy tissue. This may lead to: 3.3- bleeding with a risk of hemorrhage	Dans le cas d'une ablation de la tumeur uniquement, mon chirurgien devra couper le rein lui-même pour séparer la tumeur des tissus sains qui l'entourent. Cela peut entraîner 3.3- des saignements avec un risque d'hémorragie			
3.4- opening the collecting system with a risk of urine leakage	3.4- l'ouverture du système collecteur avec un risque de fuite d'urine			
3.5- To reduce the risk of hemorrhage at the time of tumor removal my surgeon may need to clamp (=interrupt blood-low) the renal artery	3.5- Pour réduire le risque d'hémorragie au moment de l'ablation de la tumeur, mon chirurgien peut être amené à			

	clamper (= interrompre le flux sanguin) l'artère rénale.			
3.6- Prolonged renal artery clamping is known to alter renal function so my surgeon will have to speed up the procedure to limit the length of clamping	3.6- Le clampage prolongé de l'artère rénale est connu pour altérer la fonction rénale, mon chirurgien devra donc accélérer la procédure pour limiter la durée du clampage			
3.7- In case of tumor only removal, the benefit is preservation of healthy kidney tissue	3.7- En cas d'ablation de la tumeur uniquement, l'avantage est la préservation du tissu rénal sain.			
3.8- Preserving healthy tissue from my tumor bearing kidney decreases the risk of renal insuficiency	3.8- Préserver le tissu sain de mon rein porteur de tumeur diminue le risque d'insuffisance rénale			

APPENDIX 3 – Satisfaction questionnaire (adapted from Bernhard 2016)

4/ Please, rate from 1 to 10 (1 = no help at all -> 10 = of a great help) to what extent the presentation of your personalized 3D Kidney model helped you in :

- 4.1 - Learning about the kidney itself

  1 2 3 4 5 6 7 8 9 10

  No help at all — Of a great help

- 4.2 - Learning about your disease

  1 2 3 4 5 6 7 8 9 10

  No help at all — Of a great help

- 4.3 - Understanding the surgery you will undergo

  1 2 3 4 5 6 7 8 9 10

  No help at all — Of a great help

- 4.4 - Understanding the risk of complications related to the surgery you will undergo

  1 2 3 4 5 6 7 8 9 10

  No help at all — Of a great help

4/ S'il vous plaît, notez de 1 à 10 (1 = pas d'aide du tout -> 10 = d'une grande aide) dans quelle mesure la présentation du modèle de Rein imprimé en 3D vous a aidé à :

- 4.1 – Apprendre « des notions » sur le rein lui-même

  De 1 à 10, 1 signifie ne vous a pas aidé, 10 vous a été d'une grande aide
- 4.2 – Comprendre votre maladie ;

  De 1 à 10, 1 signifie ne vous a pas aidé, 10 vous a été d'une grande aide
- 4.3 - Comprendre la chirurgie prévue,

  De 1 à 10, 1 signifie ne vous a pas aidé, 10 vous a été d'une grande aide

- 4.4 - Comprendre le risque de complications liées à l'intervention chirurgicale prévue
  De 1 à 10, 1 signifie ne vous a pas aidé, 10 vous a été d'une grande aide
