# Supplementary material for: Effects of a personalized or generic three-dimensional tumoral kidney model on patient experience and caregiver-patient interactions, before and after partial nephrectomy, a randomized trial (Rein 3D Print Personalize—UroCCR 114)
Source: PLoS One. 2025 Aug 18;20(8):e0323515. doi: 10.1371/journal.pone.0323515 (PMC12360608; doi:10.1371/journal.pone.0323515)
Supplement: S11 File — (PDF) [file pone.0323515.s011.pdf]

Effects of a personalized or generic three-dimensional tumoral kidney model on patient experience and caregiver-patient interactions, before and after partial nephrectomy (Rein 3D Print Personalize UroCCR 114)

Kidney 3D PRINT PERSONALIZE

UroCCR N° 114

Study Code : CHUBX 2023/78

Research Category: Category 2 - Minimal Risk and Constraint Research)

Version n° 2.0 du 04/06/2024

ID-RCB NUMBER : 2024-A00129-38

Cette recherche interventionnelle a obtenu le financement de ANR-21-RHUS-0015

Sponsor:
Centre Hospitalier Universitaire de Bordeaux
12, rue Dubernat
33400 Talence
FRANCE

Coordinating Investigator:
Dr Gaëlle MARGUE
CHU de Bordeaux – Hôpital Pellegrin
Service de Chirurgie Urologique et Transplantation
Place Amélie Raba-Léon
33076 Bordeaux Cedex
Tél. : 05 57 82 17 62
Courriel : gaelle.margue@chu-bordeaux.fr

Scientific Lead:
Pr Jean-Christophe BERNHARD
CHU de Bordeaux – Hôpital Pellegrin
Service de Chirurgie Urologique et Transplantation
Place Amélie Raba-Léon
33076 Bordeaux Cedex
Tél. : 05 57 82 17 62
Courriel: jean-christophe.bernhard@chu-bordeaux.fr

Methodology Support Center :
Pr Laura RICHERT
Unité de Soutien Méthodologique à la Recherche Clinique et Epidémiologique du CHU de Bordeaux
Case 75, 146 rue Léo Saignat
33076 Bordeaux Cedex
Tel.: 05 57 57 11 81 - Fax: 05 57 57 15 78
Courriel: laura.richert@chu-bordeaux.fr

Centre de Gestion des données
Mme Marthe-Aline JUTAND
CeDS EA 74-40
3 ter Place de la Victoire
33076 Bordeaux Cedex
Tel: +33 (0)5 57 57 19 92 / 06 13 05 78 61
Courriel : marthe-aline.jutand@u-bordeaux.fr

Version 4.0 was written on the 18/01/2022, protocole-form : GIRCI SOHO

Update registry

Version	Date	update
1.0	23/01/2024	initial CPP Version
1.0	29/03/2024	Modified Version
2.0	04/06/2024	Re-Modified Version

PROTOCOL SIGNATURE

« Effects of a Personalized or Generic Three-Dimensional Tumoral Kidney Model on Patient Experience and Caregiver-Patient Interactions Before and After Partial Nephrectomy »

Kidney 3D Print Personalize

UroCCR N°114

Code promoteur : CHUBX 2023/78

<u>Promoteur</u>
Centre Hospitalier Universitaire de Bordeaux
12, rue Dubernat
33 400 Talence

à Talence, le :

Le Directeur Général par intérim du CHU de Bordeaux
A. THOMAS
Et par délégation, le Directeur de la Recherche Clinique et de l'Innovation,
G. DULUC
Signature

<u>Investigateur principal</u>
Dr Gaëlle MARGUE
CHU de Bordeaux – Hôpital Pellegrin
Service de Chirurgie Urologique et Transplantation
Place Amélie Raba Léon
33076 Bordeaux Cedex
Tel : 05 57 82 17 62
Courriel : gaelle.margue@chu-bordeaux.fr

à Bordeaux, le :

Dr Gaëlle MARGUE
Signature

PRINCIPAUX CORRESPONDANTS

Investigateur principal	Dr Gaëlle MARGUE<br>CHU de Bordeaux - Hôpital Pellegrin<br>Place Amélie Raba Léon<br>33076 Bordeaux Cedex

Tél. : 05 57 82 17 62
Courriel : gaelle.margue@chu-bordeaux.fr

Responsable scientifique
Pr Jean-Christophe BERNHARD
CHU de Bordeaux - Hôpital Pellegrin
Place Amélie Raba Léon
33076 Bordeaux Cedex
Tél. : 05 57 82 17 62
Courriel : jean-christophe.bernhard@chu-bordeaux.fr

Programme Manager
Mme Solène RICARD
Service d'Urologie et Transplantation Rénale
CHU de Bordeaux – Pellegrin
33076 Bordeaux Cedex
Tél. : 05 57 82 12 94
Courriel : solene.ricard@chu-bordeaux.fr

Attachée de Recherche Clinique
Mme Clémence MORICE
Service d'Urologie et Transplantation Rénale
CHU de Bordeaux – Pellegrin
33076 Bordeaux Cedex
Tél. : 05 57 82 23 94 - Fax : 05.56.79.56.51
Courriel : clemence.morice@chu-bordeaux.fr

Unité de Soutien Méthodologique à la Recherche Clinique et épidémiologique
Service d'information médicale, Pôle Santé publique, CHU Bordeaux
146 rue Léo Saignat, case n°75
33076 Bordeaux Cedex
Tel : 05 57 57 11 29 / 14 42 - Fax : 05 57 57 15 78
Coordination Méthodologique :
Pr Laura RICHERT
Courriel : laura.richert@chu-bordeaux.fr
Biostatisticienne :
Mme Roxane COUËRON
Courriel : roxane.coueron@chu-bordeaux.fr

Unité de la Recherche et de l'Innovation en Soins et Sciences Humaines (URISH)
Mme Hélène HOARAU
Direction des soins et Direction de la recherche clinique et de l'innovation

12, rue Dubernat
33404 Talence Cedex
Tél. : 06 37 83 92 85
Courriel : helene.hoarau@chu-bordeaux.fr

Promoteur
Centre Hospitalier Universitaire de Bordeaux
12 rue Dubernat
33400 Talence

Responsable de la recherche au niveau du promoteur
Gilles DULUC - Directeur de la Recherche Clinique et de l'Innovation
Dr Anne GIMBERT - Responsable « Promotion interne »
Tél : 05 57 82 08 34 – Fax : 05 56 79 49 26
Courriel : anne.gimbert@chu-bordeaux.fr

Responsable d'Etudes Cliniques
Mme Corinne CASTERMANS
Direction de la Recherche Clinique et de l'Innovation du CHU de Bordeaux
12 rue Dubernat
33404 Talence Cedex
Tél. : 05 57 82 08 53
Courriel : corinne.castermans@chu-bordeaux.fr

Unité de sécurité et de vigilance de la recherche clinique
Direction de la recherche clinique et de l'innovation
12, rue Dubernat
33404 Talence Cedex
Tél: 05 57 82 16 26 - Fax: 05 57 82 12 62
Courriel : vigilance.essais-cliniques@chu-bordeaux.fr

Lab. Culture et Diffusion des Savoirs (CeDS)
Mme Marthe-Aline JUTAND et Mme Sarah MASANET
Laboratoire CeDS EA-7440
3 ter, Place de la Victoire
33076 Bordeaux Cedex
Tél. : 05 57 57 19 92 - Mobile : 06 13 05 78 61
Courriel : marthe-aline.jutand@u-bordeaux.fr

Coordinatrice outil UroCONNECT
Mme Anne CALLEDE
Service d'Urologie et Transplantation Rénale
CHU de Bordeaux – Pellegrin
33076 Bordeaux Cedex
Tél. : 05 57 79 60 13 - Fax : 05.56.79.56.51
Courriel : anne.callede@chu-bordeaux.fr

SOMMAIRE

RESUME DE LA RECHERCHE 9
ABSTRACT 14
1. JUSTIFICATION SCIENTIFIQUE ET DESCRIPTION GENERALE 17
1.1. ETAT ACTUEL DES CONNAISSANCES 17 1.2. HYPOTHESES DE LA RECHERCHE ET RESULTATS ATTENDUS 18 1.3. JUSTIFICATION DU FAIBLE NIVEAU D'INTERVENTION 18 1.4. RAPPORT BENEFICE / RISQUE 19 1.5. RETOMBEES ATTENDUES 20 2. OBJECTIFS DE LA RECHERCHE 20 2.1. OBJECTIF PRINCIPAL 20 2.2. OBJECTIFS SECONDAIRES 20
3. CRITERES DE JUGEMENT 21
3.1. CRITERE DE JUGEMENT PRINCIPAL 21 3.2. CRITERES DE JUGEMENT SECONDAIRES 21
4. CONCEPTION DE LA RECHERCHE 22
4.1. JUSTIFICATION DES CHOIX METHODOLOGIQUES 22 4.2. SCHEMA DE LA RECHERCHE 23 4.3. METHODES POUR LA RANDOMISATION 25
5. CRITERES D'ÉLIGIBILITE 25 5.1. CRITERES D'INCLUSION 25 5.2. CRITERES DE NON INCLUSION 25
5.3. FAISABILITE ET MODALITES DE RECRUTEMENT 25
6. STRATEGIE(S)/PROCEDURE(S)/PRODUITS DE LA RECHERCHE 26
6.1. STRATEGIE 26
6.2. PROCEDURE EXPERIMENTALE ET DE COMPARAISON 26 6.2.1. Groupe Modele 3D Imprimé Personnalise 26 6.2.2. Groupe Modèle 3D Imprimé Générique 27
7. DEROULEMENT DE LA RECHERCHE 27 7.1. CALENDRIER DE LA RECHERCHE 27
7.2. TABLEAU RECAPITULATIF DU SUIVI PARTICIPANT 28 7.3. VISITE D'INCLUSION (T0) 29
7.3.1. Recueil du consentement 29
7.3.2. Déroulement de la visite 29
7.3.3. Visite/démarche de randomisation 30
7.4. VISITES DE SUIVI 30
7.4.1. Période préopératoire (T1) 30
7.4.2. Consultation d'échange et d'information (T2) 31
7.4.3. Seconde période préopératoire (T3) 31
7.4.4. Chirurgie (T4) 31
7.4.5. Consultation post-opératoire (T5) 31 7.5. VISITE DE FIN DE LA RECHERCHE (T6) 31
7.6. REGLES D'ARRET 32
7.6.1. Arrêt de la participation d'une personne a la recherche 32
7.6.2. Arrêt de la recherche 32
7.7. DEVIATIONS AU PROTOCOLE 33
7.7.1. Arrêt prématuré et définitif de la procédure de la recherche 33
7.7.2. Participant perdu de vue 33
7.7.3. Participant inclus à tort 33
7.8. PARTICIPATION SIMULTANEE A D'AUTRES RECHERCHE, PERIODE D'EXCLUSION, INDEMNISATION ET INSCRIPTION AU FICHIER VRB 33
8. GESTION DES EVENEMENTS INDESIRABLES / EFFETS INDESIRABLES / INCIDENTS 33
9. ASPECTS STATISTIQUES 34 9.1. TAILLE D'ETUDE 34
9.2. METHODES STATISTIQUES EMPLOYEES 34 9.2.1. Stratégie d'analyse 34
9.2.2. Patients inclus dans l'analyse 35
9.2.3. Risque de première espèce 35
9.2.4. Méthodes statistiques descriptives 35

9.2.5. Méthodes statistiques comparatives 36
9.2.6. Méthodes d'analyse de données qualitatives 36
9.2.7. Logiciels utilisés 36
9.3. PLAN D'ANALYSE 36
9.3.1. Description des inclusions, des déviations et du suivi 36
9.3.2. Caractéristiques des patients à l'inclusion 36
9.3.3. Analyse de l'objectif principal 37 9.3.4. Analyse des objectifs secondaires 37
10. SURVEILLANCE DE LA RECHERCHE 38
10.1. CONSEIL SCIENTIFIQUE 38
10.1.1. Composition 38
10.1.2. Rythme des réunions 38
10.1.3. Rôle 38
10.2. Comité INDEPENDANT DE SURVEILLANCE 39
11. GESTION ET TRAITEMENT DES DONNEES ET DOCUMENTS SOURCES 39
11.1. DONNEES ET DOCUMENTS SOURCE 39 11.2. CONSIGNES POUR LE RECUEIL DES DONNEES 39 11.3. GESTION ET CIRCUIT DES DONNEES 39
11.3.1. Logiciel de gestion de données 39
11.3.1.1. Logiciel utilisé 39
11.3.1.2. Hébergement des données 40
11.3.1.3. Sécurité des données 40
11.3.2. Saisie des données 40
11.3.3. Codage des données 41
11.3.4. Contrôles des données 41
11.3.5. Transfert des données 41
11.4. CONFIDENTIALITE DES DONNEES 41 11.5. CONSERVATION DES DOCUMENTS ET DES DONNEES RELATIFS A LA RECHERCHE 42 11.6. CESSION DES DONNEES 42
12. CONTROLE ET ASSURANCE QUALITE 43
12.1. ACCES AUX DONNEES 43 12.2. CONTROLE QUALITE 43 12.3. AUDIT ET INSPECTION 43
13. CONSIDERATIONS ETHIQUES ET REGLEMENTAIRES 44
13.1. CONFORMITE AUX TEXTES DE REFERENCE 44 13.2. MODIFICATIONS AU PROTOCOLE 44
14. RAPPORT FINAL 45
15. REGLES RELATIVES A LA PUBLICATION 45 15.1. COMMUNICATIONS SCIENTIFIQUES 45
15.2. COMMUNICATION DES RESULTATS AUX PARTICIPANTS 45
REFERENCES BIBLIOGRAPHIQUES 46

LISTE DES ABREVIATIONS

ANSM : Agence Nationale de Sécurité du Médicament et des produits de santé

CeDS : Culture et Diffusion des Savoirs

CNIL : Commission Nationale de l'Informatique et des Libertés

CPP : Comité de Protection des Personnes

CREDIM : Centre de Recherche et Développement en Informatique Médicale

DM : Data Manager

DREES : Direction de la Recherche, des Etudes, de l'Evaluation et des Statistiques

HDS : Hébergeurs de Données de Santé

HLSEU-Q16 : European Health Literacy Survey Questionnaire

ITT : Intention-de-traiter

IUT : Institut Universitaire de Technologie

OMS : Organisation Mondiale de la Santé

R3DP-P : Etude Rein 3D Print-Personalize

RCP : Réunion de Concertation Pluridisciplinaire

SHS : Sciences Humaines et Sociales

URISH : Unité de la Recherche et de l'Innovation en Soins et Sciences Humaines

UroCCR : Réseau français de recherche sur le cancer du rein

USMR : Unité de Soutien Méthodologique à la Recherche Clinique et Epidémiologique

RESUME DE LA RECHERCHE

PROMOTEUR	Centre Hospitalier Universitaire de Bordeaux<br>12, rue Dubernat, 33400 Talence, France
INVESTIGATEUR COORDONNATEUR	Dr Gaëlle MARGUE
RESPONSABLE SCIENTIFIQUE	Pr Jean-Christophe BERNHARD
ACRONYME ET TITRE	R3DP-P - Impacts d'un modèle tridimensionnel personnalisé ou générique de rein tumoral sur l'expérience des patients et les interactions entre professionnels et patients avant et après néphrectomie partielle
JUSTIFICATION / CONTEXTE	Une étude pilote de 2015 a montré le bénéfice de l'utilisation de modèles personnalisés de rein imprimés en 3D comme outil de médiation éducatif lors de la consultation préopératoire de patients programmés pour une néphrectomie partielle robot-assistée. La compréhension de la pathologie et de la chirurgie était ainsi facilitée. Le patient pouvait mieux saisir les enjeux chirurgicaux de sa maladie entraînant ainsi une meilleure satisfaction au cours de sa prise en charge.<br>Les patients pris en charge sont issus de cultures et de milieux sociaux divers. Il est donc important de tenir compte de cette hétérogénéité dans la relation soignant-soigné, posant ainsi les bases de la médecine personnalisée. La mesure de la littératie en santé permet de repérer et de décrire cette hétérogénéité des profils-patient. Il a ainsi été montré que les patients à faible niveau de littératie ont un niveau d'anxiété plus élevé, notamment par rapport à la chirurgie, ainsi qu'un moins bon niveau de récupération post-opératoire. De nombreuses études ont été menées afin de développer des stratégies d'amélioration de la littératie en santé des patients et de la méthode d'information des soignants. Elles soulignent, plus spécifiquement, l'importance d'améliorer la compréhension des informations communiquées aux patients.<br>L'utilisation d'un modèle de rein imprimé en 3D, personnalisé ou générique, au cours d'une consultation préopératoire d'information spécifique avec le patient, pourrait donc modifier la compréhension de sa pathologie, de sa prise en charge ainsi que ses interactions avec l'ensemble des professionnels de santé.
OBJECTIFS	Objectif principal :<br>Etudier les effets de l'utilisation d'un modèle personnalisé de rein imprimé en 3D versus un modèle générique de rein imprimé en 3D comme outil de médiation, tout au long du parcours de soins, sur l'expérience des patients et leurs interactions avec les professionnels, avant et après néphrectomie partielle.

	Objectifs secondaires :<br>1. Décrire par des études qualitatives les interactions entre les professionnels et les patients en spécifiant les situations d'échanges et leurs environnements.<br>2. Comparer par des études qualitatives les différences de discours et les usages des termes employés lors de l'utilisation des différents outils au cours des interactions entre patients, professionnels et entourage.<br>3. Etudier par une approche qualitative l'utilisation du modèle de rein imprimé en 3D introduit lors d'une consultation d'information médicale jusqu'à la visite post-opératoire.<br>4. Comparer la compréhension du patient concernant l'anatomie et la stratégie chirurgicale selon le type d'outil, personnalisé ou générique, avant et après l'opération à partir des questionnaires et entretiens.<br>5. Comparer le niveau de littératie en santé entre les deux groupes et aux différents temps (HLSEU-Q16 : European Health Literacy Survey Questionnaire).<br>6. Décrire par des études qualitatives les modifications perçues par les professionnels dans la prise en charge des patients suite à l'intégration de l'outil de médiation auprès des patients.
CRITERES DE JUGEMENT	CRITERE de JUGEMENT PRINCIPAL :<br>Critère mixte permettant de décrire l'expérience patient à partir des entretiens. Les observations ainsi que les trois questionnaires utilisés viennent compléter les analyses des données qualitatives. L'expérience des patients sera également analysée au regard des données cliniques et démographiques issues de la base de données UroCCR.<br><br>CRITERES de JUGEMENT SECONDAIRES :<br>1. Déroulement des interactions entre professionnels et patients : description du déroulé des interactions selon le modèle utilisé, en spécifiant la pratique ainsi que le vocabulaire et le contenu du discours employé.<br>2. Fréquence des termes employés en adéquation avec l'utilisation des différents outils lors des interactions.<br>3. Usage du modèle 3D pendant la prise en charge : description de la vie du modèle pendant l'ensemble de la prise en charge (de la visite d'inclusion à la visite post opératoire).<br>4. Niveau de compréhension de l'anatomie rénale et des enjeux chirurgicaux.<br>5. Evolution du score moyen du questionnaire de littératie HLSEU-Q16 mesuré (littératie en santé).<br>6. Effets auprès des professionnels : évaluation qualitative des modifications de pratiques suite à l'intégration des modèles 3D dans la pratique du service.

SCHEMA DE LA RECHERCHE	Étude monocentrique, mixte (qualitative et quantitative), reposant sur des observations (situations d'interaction), des entretiens (professionnels et patients) et des questionnaires (patients).

	Patients<br>- Etude interventionnelle, prospective randomisée selon un ratio 1:1, en 2 bras parallèles ouverts : l'un ayant une visite préopératoire avec un modèle de rein imprimé en 3D personnalisé, l'autre avec un modèle de rein imprimé en 3D générique<br><br>Professionnels<br>Etude non interventionnelle de l'expérience des professionnels lors de la prise en charge de patients ayant bénéficié d'une visite préopératoire utilisant un modèle de rein imprimé en 3D personnalisé ou générique.
CRITERES D'INCLUSION	Patients<br>- Age ≥ 18 ans<br>- Prise en charge chirurgicale programmée par néphrectomie partielle coelioscopique avec assistance robotique (1ère prise en charge pour une tumeur du rein unilatérale ou bilatérale)<br>- Consentement libre, éclairé et signé pour la base de données UroCCR<br>- Consentement libre, éclairé et signé pour le protocole Rein3D Personalize<br>- Personne affiliée ou bénéficiaire d'un régime de sécurité sociale<br><br>Professionnels<br>- Professionnel exerçant auprès des patients pris en charge dans le service d'Urologie, d'andrologie et transplantation rénale du CHU de Bordeaux depuis au moins deux mois avant l'implémentation des outils 3D<br>- Consentement libre, éclairé et signé

CRITERES DE NON INCLUSION	Patients<br>- Métastatiques au moment de l'inclusion<br>- Antécédent de cancer du rein<br>- Rein unique au moment de l'inclusion<br>- Absence de scanner préopératoire ou scanner de mauvaise qualité ne permettant pas la réalisation d'une modélisation 3D fiable<br>- Personne faisant l'objet d'une mesure de protection légale (sauvegarde de justice, tutelle ou curatelle)<br>- Difficultés à comprendre et à s'exprimer en français<br><br>Professionnels<br>- Professionnel en stage d'une durée de moins de 6 mois - Professionnel sans contact avec les patients
STRATEGIES/PROCEDURES DE LA RECHERCHE	Patients<br>Lors de l'inclusion dans l'étude, les patients seront randomisés en 2 bras, correspondants aux différents supports d'information utilisés lors de la consultation d'information :
	1. Groupe modèle 3D imprimé personnalisé : temps d'échange avec pour support d'information le modèle tridimensionnel physique imprimé du rein à opérer<br>2. Groupe modèle 3D imprimé générique : temps d'échange avec pour support d'information un modèle tridimensionnel physique imprimé, issu d'une mallette de huit modèles, et correspondant au plus près à la situation du patient<br><br>Professionnels<br>Pas d'intervention spécifique

DEROULEMENT DE L'ETUDE	Patients : 60 inclusions<br>- Une première consultation avec le chirurgien entre 1 et 6 mois avant la chirurgie pour inclusion du patient<br>- Un entretien avec la chercheuse en SHS dans les 15 jours suivant la visite d'inclusion, ainsi que la passation du questionnaire HLSEUQ16, et du questionnaire de connaissances.<br>- Une seconde consultation avec le chirurgien pour information à l'aide du modèle 3D imprimé personnalisé ou générique selon le groupe de randomisation, entre 7 et 30 jours avant la chirurgie<br>- Un second entretien avec la chercheuse SHS dans les 15 jours suivant la seconde consultation, ainsi que la passation du questionnaire de connaissances.<br>- Une consultation post-opératoire à 1 mois de la chirurgie suivie d'un dernier entretien avec la chercheuse SHS complété de la passation du questionnaire HLSEUQ16, du questionnaire de connaissances ainsi que du questionnaire de satisfaction.<br><br>Professionnels : 30 inclusions<br>- 1 entretien individuel par professionnel dans l'année suivant le début de l'étude
TAILLE D'ETUDE	60 patients et 30 professionnels
DUREE DE LA RECHERCHE	Durée de la période d'inclusion : 18 mois<br>Durée de participation pour les patients : entre 3 et 6 mois<br>Durée de participation pour les professionnels : 24 mois<br><br>Durée totale de la recherche : 38 mois (3 ans et 2 mois) inclusion, suivi, analyse et valorisation.
ANALYSE STATISTIQUE DES DONNEES	Les données socio-démographiques et cliniques des patients seront décrites en termes de fréquences pour les données qualitatives et de moyennes et écart-types pour les données quantitatives permettant ainsi la comparaison entre les deux groupes de patients.<br>Les données issues des questionnaires remplis par les patients feront l'objet d'analyse descriptive (distribution des scores obtenus par les patients pour le questionnaire littératie HLS-EU16 au début et à la fin, description du questionnaire de satisfaction au dernier temps). Cette

	analyse descriptive sera réalisée de manière globale et par groupe de randomisation.<br>Une analyse de comparaison entre les groupes de randomisation sera réalisée, systématiquement sans ajustement et avec ajustement sur les facteurs pronostiques initiaux dont la répartition pourrait être, malgré la randomisation, déséquilibrée.<br>La description de l'expérience patient sera réalisée à partir de l'analyse des contenus des entretiens de manière transversale pour chaque temps, mais aussi par une analyse longitudinale. L'évolution de la compréhension de la maladie et de la chirurgie sera étudiée par l'analyse des contenus des entretiens et des questionnaires et une comparaison sera réalisée entre les deux groupes.<br>Les entretiens des professionnels feront également l'objet d'une analyse de contenu.<br>Toutes les analyses seront réalisées avec le support du logiciel Nvivo et effectuées par les chercheurs en SHS.<br>Les données seront analysées par l'équipe de chercheuses en SHS pour assurer l'analyse croisée des données qualitatives et quantitatives.<br>L'analyse principale sera effectuée par sous-traitement pour les patients, c'est à dire que les données de tous les participants doivent être analysées au regard du modèle 3D imprimé réellement administré, même si la randomisation en a décidé autrement, sur données disponibles.
RETOMBEES ATTENDUES	Les retombées attendues de l'étude mixte (qualitative et quantitative) R3DP-P sont :<br>Pour les patients ○ Acquérir des connaissances sur l'apport de la matérialisation de la pathologie dans sa compréhension.<br>○ Evaluer l'appropriation de l'outil 3D comme outil de médiation et de compréhension (littératie) par les patients.<br>○ Améliorer le vécu de son hospitalisation et de l'intervention.<br><br>Pour les professionnels ○ Evaluer l'appropriation de l'outil 3D comme outil de médiation par les professionnels.<br><br>Pour le système de santé ○ Identifier des spécificités d'usages selon la personnalisation ou non du modèle et évaluer le bénéfice de la personnalisation de l'outil 3D ainsi que l'intérêt de son implémentation en pratique courante.

ABSTRACT

This research has been registered in http://www.clinicaltrials.gov/ under the n° NCT06379698

Effects of a personalized or generic three-dimensional tumoral kidney model on patient experience and professional-patient interactions, before and after partial nephrectomy. R3DP-P

The University Hospital of Bordeaux is the sponsor of this research.

This research will be conducted with the support of ANR-21-RHUS-0015.

Brief summary: We aim to compare preoperative information and patient experience using a personalized versus a generic 3D printed models of patients' tumoral kidney before and after nephron-sparing surgery. The main outcome measure will be based on semi-structured interviews with the patient and the carers.

Detailed description: Use of tools to decrease anxiety and enhance understanding prior to surgery is a key point in comprehensive care that is way not enough promoted for now.
A pilot study from 2015 demonstrated the benefits of using personalized 3D-printed kidney models as educational mediation tools during the pre-operative consultation of patients scheduled for robot-assisted partial nephrectomy. The patient's understanding of his pathology and of the surgery was thus facilitated, leading to greater satisfaction during treatment.

For personalized medicine, it is important to consider the heterogeneity in culture and social backgrounds of patients in the doctor-patient relationship. Measuring health literacy help to identify and describe this heterogeneity in patient profiles. Patients with low literacy levels have higher levels of anxiety, particularly regarding surgery, and poorer post-operative recovery. Some studies highlight the importance of improving patient understanding with strategies to improve patients' health literacy and information provided by carers.

The aim of this study is therefore to investigate the benefits of using a personalized 3D-printed kidney model versus a generic 3D-printed kidney model as a mediation tool, all along the care pathway, on patients' experience and their interactions with carers, before and after partial nephrectomy.

To achieve this aim, 60 patients planned for robot-assisted laparoscopic partial nephrectomy will be randomized, in a 1:1 ratio, between the use of a personalized 3D kidney model and a generic 3D kidney model.

3D models will be presented to the patients according to the allocated study group during a preoperative education consultation. All patients will complete questionnaires about their health literacy level, their knowledge of kidney anatomy and tumor and their satisfaction about the using 3D-printed kidney models as educational mediation tools. Semi-structured interviews will be conducted at three different times: between the first consultation with the surgeon and the preoperative education consultation, between this second consultation and the surgery and after the post-operative consultation.

Primary outcome:

To study the effects of using a personalized versus a generic 3D-printed kidney model as a mediation tool on patients' experience and interactions with carers, before and after partial nephrectomy.

This is a mixed-type study designed to combine data from different collection tools (observations, interviews and questionnaires). The criteria studied are:

For the patient:

- Qualitative assessment of the patient experience
- Changes in the average score of the HLSEU-Q16 literacy questionnaire - Level of understanding of renal anatomy and of the surgical issues

For the carers:

- Qualitative assessment of changes in practice following the integration of 3D models into the department

Secondary outcomes:

1. Description of interactions between carers and patients, specifying the situations and the environments, the practice, vocabulary and content of the discourse used during the entire care process.
2. Comparison of the differences in speech and terminology used when using the different tools during interactions between patients, carers and patients' entourage with systematic analysis of interviews.
3. Studying the use of the 3D-printed kidney model introduced during the medical information consultation up to the post-operative visit by describing the life of the model throughout the entire care process (from the inclusion visit to the post-op visit).
4. Comparison of the patient's understanding of anatomy and surgical strategy according to the type of tool (personalized vs generic 3D printed model) before and after the surgery (T1, T3 and T6).
5. Comparison of the level of health literacy between the two groups at different times with changes in the average score on the literacy questionnaire (HLSEU-Q16: European Health Literacy Survey Questionnaire; T1 and T6).
6. Description of the changes perceived by carers in patients management following the integration of the mediation tool with patients by qualitatively assessing changes in practices following the integration of 3D models during the entire care process.

Study design: Monocentric, randomized and controlled clinical trial Eligibility criteria:

- Patients:
  - Adult patients (≥ 18 years of age)
  - Scheduled for surgical management by laparoscopic robotic-assisted partial nephrectomy (1st management for a unilateral or bilateral kidney tumor)
  - Expressed consent for integration in the UroCCR database o Expressed consent for participation in the Rein-3D Personalize study o Patients affiliated or benefiting from social security system

- Carers:
  - Carers working with patients treated in the Urology, Andrology and Renal Transplant Department of the Bordeaux University Hospital for at least two months prior to the implementation of 3D models
  - Free, informed and signed consent

Arm number or label and arm type:

- Group 1: 3D Printed Personalized Model Group: exchange time with the printed threedimensional model of the patient's kidney as information support.
- Group 2: 3D Printed Generic Model Group: exchange time with the printed threedimensional generic kidney model, approximating the patient's situation, as information support.

Number of subjects: 60 patients and 30 carers -

Statistical analysis :

A mixed analysis of the data will be realised with a triangulation approach. The sociodemographic data and the medical profiles of the patients will be described (in terms of frequencies for the qualitative data and with means and standard deviations for the quantitative data) and will be compared between the two groups. Data from patients questionnaires will be analysed descriptively (distribution of patient scores for the HLS-EU16 litteracy questionnaire at start and end and at the end distribution of satisfaction with use of the 3D model.). The analysis of the patients' experience will be based on the content of the cross-sectional interviews at each stage, but also on a longitudinal analysis. The evolution of the understanding of the disease and the surgery will be studied by analysing the content of the interviews and questionnaires and a comparison between the two groups will be made.

Content of the carers interviews will also be analysed.

Conditions: kidney cancer surgery

Keywords: Renal-Cell Carcinoma, Nephron-Sparing Surgery, 3D Model, 3D Printing, Personalized Medicine, Literacy Questionnaire, Interviews.

1. JUSTIFICATION SCIENTIFIQUE ET DESCRIPTION GENERALE

1.1. ETAT ACTUEL DES CONNAISSANCES

En 2015, une étude pilote a évalué l'utilisation d'un modèle de rein personnalisé imprimé en 3D chez 7 patients anglophones pour lesquels une néphrectomie partielle était envisagée (1). Il a ainsi été montré que ces modèles 3D pouvaient être utilisés comme outils de médiation éducatifs afin de faciliter la compréhension par le patient de sa pathologie et de sa prise en charge chirurgicale.

Une meilleure communication permettrait au patient de mieux saisir les enjeux de la chirurgie et lui procurerait une meilleure satisfaction quant au déroulement des visites et du suivi médical (2).
Cependant, comme l'indique l'étude de Curchod et al., « les soins donnés dans notre société s'adressent à des personnes de cultures et de milieux sociaux divers et variés » (3). Il est donc essentiel de tenir compte de cette hétérogénéité dans la relation soignant-soigné, définissant ainsi la notion de médecine personnalisée. La littératie en santé, définie par l'OMS comme « les aptitudes cognitives et sociales qui déterminent la motivation et la capacité des individus à obtenir, comprendre et utiliser des informations d'une façon qui favorise et maintienne une bonne santé » permet de repérer et décrire cette hétérogénéité des profils-patient. Selon une étude de la DREES (Direction de la recherche, des études, de l'évaluation et des statistiques), publiée en mai 2023, 10,7 % de la population estime avoir des difficultés de compréhension de l'information médicale (4). Dans le cas d'une prise en charge chirurgicale, il a été montré que les patients avec un faible niveau de littératie avaient un niveau d'anxiété plus élevé (5), notamment par rapport à l'intervention (6) et un moins bon niveau de récupération postopératoire (7). De nombreuses études sont menées pour développer des stratégies d'amélioration de la littératie en santé des patients en cancérologie et des méthodes d'information des soignants (8–10). Elles soulignent l'importance d'améliorer la compréhension des informations données aux patients.

Lors de sa prise en charge à l'hôpital, un patient diagnostiqué d'une tumeur rénale va rencontrer différents professionnels de santé. Les interactions qui vont avoir lieu entre le patient et chaque professionnel vont dépendre du lieu, du contexte, des caractéristiques personnelles du patient (âge, genre, profession, niveau de littératie…) mais aussi des diverses conceptions que le professionnel a du malade en tant que sujet. Par exemple, si le patient est vu comme acteur de ses soins, c'est à dire pouvant acquérir et développer des compétences de soins au travers de la relation professionnel-soigné, alors les attentes des professionnels envers ce patient et ses compétences relationnelles seront importantes (11).

L'introduction de nouvelles technologies dans la prise en charge du patient peut donc modifier les comportements des professionnels (12). Ainsi, l'utilisation d'un modèle de rein imprimé en 3D peut modifier la compréhension par le patient de sa pathologie et de sa prise en charge ainsi que ses interactions avec l'ensemble des professionnels.
En 2022, l'étude de Scott et al., basée sur un protocole similaire à l'étude de Bernhard et al. (1) et utilisant le même questionnaire de compréhension, a confirmé l'impact de ces modèles personnalisés sur la compréhension du patient tout en soulevant l'impact financier de leur

fabrication (13). L'étude de Bernhard et al. a été réalisée avec des modèles 3D imprimés personnalisés, c'est-à-dire une reproduction du rein du patient réalisée à partir du scanner préopératoire (1). Néanmoins, les coûts et les délais de production des modèles peuvent rendre difficile leur intégration en pratique clinique lors de la consultation préopératoire. Il paraît donc intéressant d'évaluer l'utilisation de différents modèles non personnalisés mais représentant les différentes situations tumorales classiquement retrouvées.

L'ajout d'une consultation préopératoire d'information spécifique, utilisant un modèle de rein imprimé en 3D personnalisé ou générique, peut impacter différemment (en fonction du modèle), l'expérience du patient, son niveau de littératie ainsi que ses interactions avec les professionnels de santé.
Cette étude s'inscrit dans le cadre du programme RHU Digital Urology 3D qui comprend plusieurs études visant à évaluer l'apport des modèles 3D chez les patients pris en charge par néphrectomies partielles laparoscopiques robot-assistée (exemple : l'étude ANXIETY menée par le CHU de Bordeaux, NCT06035211). L'étude Rein 3D Print-Personalize (R3DP-P) évaluera les situations informationnelles tout au long de la prise en charge du patient dans le service d'Urologie, en fonction du type de modèle utilisé lors de la consultation pré-opératoire d'information (générique ou personnalisé), à travers une approche anthropologique.

1.2. HYPOTHESES DE LA RECHERCHE ET RESULTATS ATTENDUS

L'étude R3DP-P évalue l'aspect personnalisé de l'outil imprimé en 3D, son intérêt pour le patient (diagnostic d'une tumeur rénale accessible à un traitement chirurgical conservateur) et son impact sur sa compréhension de la maladie et de sa prise en charge ainsi que sur ses relations avec les professionnels de santé.

L'hypothèse principale est que l'aspect personnalisé du modèle imprimé en 3D entraîne l'utilisation du modèle comme outil de médiation lors des interactions sociales du patient avec ses proches et avec les professionnels de santé pour évoquer sa maladie, ou encore lors de l'élaboration de ses connaissances. Cette utilisation traduirait une appropriation du modèle 3D personnalisé.
De plus, on suppose que l'observation et la manipulation par le patient de la représentation 3D de « son » rein lui permettrait de mieux appréhender sa pathologie tout au long du parcours de soins.

L'étude R3DP-P est basée sur une approche d'anthropologie didactique de la santé, où la réflexivité du chercheur principal et le terrain sont le moteur de la méthode scientifique. Ainsi, les hypothèses de travail sont les suivantes :

- Hypothèse 1 : Le modèle de rein personnalisé imprimé en 3D permet au patient de « mieux » appréhender sa pathologie et l'intervention chirurgicale.
- Hypothèse 2 : Le modèle de rein personnalisé imprimé en 3D facilite la communication sur la pathologie avec son entourage.
- Hypothèse 3 : Le modèle rénal personnalisé imprimé en 3D améliore l'alliance thérapeutique entre les patients et les professionnels.
- Hypothèse 4 : Le modèle rénal personnalisé imprimé en 3D entraîne une modification des pratiques informationnelles des professionnels.

1.3. JUSTIFICATION DU FAIBLE NIVEAU D'INTERVENTION

La nature de l'étude R3DP-P ne comporte pas de risques et est associée à des contraintes minimes pour les patients.
Cette recherche n'entraîne aucune modification des indications et stratégies de prise en charge clinique du patient, telles que validées par l'équipe chirurgicale selon les recommandations en vigueur et la conclusion de la réunion de concertation pluridisciplinaire (RCP).
Seule une consultation d'information préopératoire est ajoutée à sa prise en charge conventionnelle, 7 à 30 jours avant l'opération, délivrée par un médecin : il s'agira d'un temps spécifique médié par un modèle 3D imprimé et dédié à l'information du patient.

En ce qui concerne les professionnels qui auront donné leur accord de participation, les entretiens seront réalisés par les chercheuses en SHS à leur meilleure convenance et dans le lieu de leur choix afin de faciliter la parole, de ne pas interférer avec les prises en charge et ne pas bouleverser leur quotidien.

La méthode de recherche est basée sur des entretiens et des questionnaires de compréhension de la pathologie ainsi que des observations. Si au décours d'un entretien avec un patient ou un professionnel une chercheuse note un besoin de soutien psychologique, elle orientera le participant vers un professionnel de santé.

Les chercheuses SHS auront une place d'observatrices pour comprendre les différentes situations d'interaction entre patients et professionnels et s'assureront de ne jamais perturber les situations (notamment les consultations). Une demande orale sera toujours effectuée pour recueillir l'accord des protagonistes de la situation observée.

1.4. RAPPORT BENEFICE / RISQUE

Le principal bénéfice attendu est l'amélioration de la compréhension de l'information préopératoire, condition essentielle à une meilleure préparation avant la chirurgie.

Patients
Par ailleurs, l'ajout d'une consultation médiée dans le parcours de soins peut être considérée comme un bénéfice car elle offre la possibilité au patient de poser toutes ses questions avant l'opération. Elle oblige néanmoins le patient à revenir au sein du centre de soins pour un temps supplémentaire.

L'intervention de l'étude consistant à participer à des entretiens de recherche et à répondre à des questionnaires (non diagnostiques), il n'existe aucun risque physique au cours de la participation.
Néanmoins, il est possible que le fait de poser des questions en lien avec la présence de tumeurs rénales entraîne d'autres interrogations. En effet, les questions interrogent le ressenti du patient. On peut imaginer que le patient démarre alors un cheminement de type introspectif qu'il n'avait pas eu auparavant et qui peut avoir des conséquences psychologiques que l'on estime modérées. Il est à souligner que la prise en charge globale du patient s'inscrit dans un réseau de soins

comportant un accompagnement soignant rapproché ainsi que des interlocuteurs (notamment des psychologues) disponibles tout au long de son suivi.

Professionnels
Le protocole de l'étude R3DP-P, qualitatif et non interventionnel, repose sur des observations et des entretiens n'ayant pas d'effet attendu sur les professionnels ou la prise en charge des patients.

1.5. RETOMBEES ATTENDUES

Les retombées attendues de l'étude mixte (qualitative et quantitative) R3DP-P sont :

- Pour les patients
  - Acquérir des connaissances sur l'apport de la matérialisation de la pathologie dans sa compréhension.
  - Evaluer l'appropriation de l'outil 3D comme outil de médiation et de compréhension (littératie) par les patients.
  - Améliorer le vécu de l'hospitalisation et de l'intervention.

- Pour les professionnels
  - Evaluer l'appropriation de l'outil 3D comme outil de médiation par les professionnels.

- Pour le système de santé :
  - Identifier des spécificités d'usages selon la personnalisation ou non du modèle et évaluer le bénéfice de la personnalisation de l'outil 3D ainsi que l'intérêt de son implémentation en pratique courante.

2. OBJECTIFS DE LA RECHERCHE

2.1. OBJECTIF PRINCIPAL

Etudier par des études qualitatives les effets de l'utilisation d'un modèle personnalisé de rein imprimé en 3D versus un modèle générique de rein imprimé en 3D comme outil de médiation, tout au long du parcours de soins, sur l'expérience des patients et leurs interactions avec les professionnels, avant et après néphrectomie partielle.

2.2. OBJECTIFS SECONDAIRES

1. Décrire par des études qualitatives les interactions entre les professionnels et les patients en spécifiant les situations d'échanges et leurs environnements.
2. Analyser par des études qualitatives les différences de discours et les usages des termes employés lors de l'utilisation des différents outils au cours des interactions entre patients, professionnels et entourage.
3. Etudier par une approche qualitative l'utilisation du modèle de rein imprimé en 3D introduit lors d'une consultation d'information médicale jusqu'à la visite post-opératoire.

4. Comparer la compréhension du patient concernant l'anatomie et la stratégie chirurgicale selon le type d'outil, personnalisé ou générique, avant et après l'opération à partir des questionnaires et entretiens.
5. Comparer le niveau de littératie en santé entre les deux groupes et aux différents temps (HLSEU-Q16 : European Health Literacy Survey Questionnaire).
6. Décrire par des études qualitatives les modifications perçues par les professionnels dans la prise en charge des patients suite à l'intégration de l'outil de médiation auprès des patients Afin de répondre aux objectifs présentés ci-dessus l'étude se décline sur deux populations : les patients et les professionnels.

3. CRITERES DE JUGEMENT

3.1. CRITERE DE JUGEMENT PRINCIPAL

Evaluation qualitative de l'expérience patient
Il ne s'agit pas d'un critère unique mais d'un ensemble d'éléments permettant de décrire l'expérience patient à partir des entretiens. Les observations ainsi que les trois questionnaires utilisés (questionnaires de littératie, de connaissances et de satisfaction : annexes 1, 2 et 3) viennent compléter les analyses des données qualitatives. L'expérience des patients récoltée sera également analysée au regard des données cliniques et démographiques issues de la base de donnée UroCCR.

3.2. CRITERES DE JUGEMENT SECONDAIRES

Déroulement des interactions entre professionnels et patients : description du déroulé des interactions selon le modèle utilisé, en spécifiant la pratique ainsi que le vocabulaire et le contenu du discours employé.

Fréquence des termes employés en adéquation avec l'utilisation des différents outils lors des interactions : comparaison des différences de discours et de terminologie lors de l'utilisation des différents outils au cours des interactions entre les patients, les soignants et l'entourage des patients avec l'analyse systématique des entretiens.

Usage du modèle 3D pendant la prise en charge : description de la vie du modèle pendant l'ensemble de la prise en charge (de la visite d'inclusion à la visite post opératoire).

Niveau de compréhension de l'anatomie rénale et des enjeux chirurgicaux mesuré à partir de la traduction du questionnaire proposé par Bernhard et al. (1) (annexe 2). Ce questionnaire est composé de 4 parties : les dimensions physiologique et anatomique, la connaissance de la maladie et des caractéristiques de la tumeur, la compréhension de la procédure chirurgicale et des risques de complication associés. Ces dimensions seront étudiées avant et après la présentation du modèle et comparées selon le modèle utilisé. La dernière partie du questionnaire reposant sur l'évaluation de la satisfaction du patient sera présentée à la fin de la période postconsultation post-opératoire (T6) (Annexe 3).

Evolution du score moyen du questionnaire de littératie HLSEU-Q16 mesuré (littératie en santé)
L'évolution du score de littératie en fonction du type d'outil de médiation utilisé au cours de la consultation d'information sera évaluée par la mesure du HLSEU-Q16 à l'inclusion et à 1 mois post-opératoire. Ce questionnaire est basé sur un modèle incluant quatre compétences liées au traitement de l'information en santé : l'accès, la compréhension, l'évaluation et l'application de l'information sur la santé (annexe 1). Ces quatre compétences sont explorées dans trois contextes de santé : soins en santé, préventions des maladies et promotion de la santé. Le HLSEU-Q16 est composé de 16 items. Toutes les dimensions sont explorées à l'exception de la dimension « appliquer l'information en promotion de la santé ». Cette version courte a été validée en français par Rouquette et al. en 2018 (15).
Chaque item est noté sur une échelle à quatre modalités : « très facile », « facile », « difficile » et « très difficile ». Pour la cotation, le score de chaque item est ramené à un score binaire, les modalités « très facile » et « facile » cotant pour 1 alors que « difficile » et « très difficile » pour 0. Le score total peut alors varier de 0 à 16. Pour faciliter l'interprétation, ils peuvent être classés en trois niveaux de littératie : inférieur ou égal à 8 traduisant un niveau inadéquat, de 9 à 12 traduisant un niveau problématique et strictement supérieur à 12, un niveau adéquat.
La répartition des trois niveaux de score sera décrite en préopératoire et en post-opératoire dans chaque bras.
Validée en français, cette échelle courte du « European Health Literacy Survey Questionnaire » (14) présente des qualités psychométriques acceptables et permet un temps de passation réduit pour les participants, ces derniers ayant plusieurs questionnaires à remplir sur le même temps dans le cadre de l'étude.

Effets auprès des professionnels
Evaluation qualitative des modifications de pratiques suite à l'intégration des modèles 3D dans la pratique du service.

4. CONCEPTION DE LA RECHERCHE

4.1. JUSTIFICATION DES CHOIX METHODOLOGIQUES

Choix de la démarche mixte

L'étude a pour objectif la compréhension de l'expérience des patients lors de l'introduction d'un nouvel outil dans la communication soignant-patient, tout au long du parcours de soin. L'introduction d'un nouvel outil de médiation dans la prise en charge du patient peut impacter les interactions patients-professionnels. Il est donc nécessaire de comprendre comment cet outil, qu'il soit personnalisé ou générique, va accompagner le patient tout au long de sa prise en charge.

C'est pourquoi une démarche compréhensive des interactions entre soigné/professionnel et soigné/entourage, au cours des processus informationnels et éducatifs médiés par un outil personnalisé ou générique, est indiquée comme schéma d'étude.

Aussi, l'étude R3DP-P s'ancre dans une approche compréhensive des interactions entre patients et professionnels, telle que développée en anthropologie de la santé (16,17) :

- Approche ethnographique (c'est-à-dire basée sur des observations de situations d'information notamment lors des interactions entre professionnels/soignés, professionnels/professionnels, soignés/soignés…) et plus globalement anthropologique (à partir d'entretiens semi-directifs individuels explorant l'expérience des patients, l'éducation en santé, la littératie en santé) (18).
- Approche en Sciences de l'éducation (à partir des mêmes entretiens et observations) pour comprendre les situations d'information qui se déclinent au cours du circuit du patient, décrire les circuits de diffusions des savoirs et de construction de connaissances des différents acteurs, pour comprendre le travail de transposition réalisé par les professionnels lors des interactions avec les patients (19).

Par ailleurs, une approche plus quantitative vient compléter l'approche compréhensive et didactique et permet d'interroger l'impact de l'intégration du modèle sur la compréhension de la pathologie par le patient et son niveau de littératie en santé. Cette approche sera réalisée via le questionnaire validé de littératie HLSEU-Q16, le questionnaire de compréhension de la pathologie et de satisfaction, créé en interne, et repris au niveau international dans l'étude de Scott (13) (annexe 1, 2 et 3).

4.2. SCHEMA DE LA RECHERCHE

Il s'agit d'une étude monocentrique, menée au sein du service d'Urologie, d'andrologie et transplantation rénale du CHU de Bordeaux. C'est une étude mixte (qualitative et quantitative) reposant sur des observations (situations d'interaction), des entretiens (professionnels et patients) et des questionnaires (patients).

Patients
- Etude interventionnelle, prospective randomisée, en 2 bras parallèles ouverts : l'un ayant une visite préopératoire avec un modèle de rein imprimé en 3D personnalisé, l'autre avec un modèle de rein imprimé en 3D générique, - Inclusion prospective.

Les patients seront randomisés avec un ratio 1:1 à deux groupes
: - Groupe 1 : Groupe Modèle 3D imprimé personnalisé,
- Groupe 2 : Groupe Modèle 3D imprimé générique.

Professionnels
Etude non interventionnelle de l'expérience des professionnels lors de la prise en charge de patients ayant bénéficié d'une visite préopératoire utilisant un modèle de rein imprimé en 3D personnalisé ou générique. A noter qu'une étude de l'expérience des professionnels avant l'implémentation des modèles 3D a été réalisée dans le cadre du projet Rein-3D Print PERSONALIZE– Pro Before– Hors Loi Jardé (numéro de référence CER-BDX 2023 – 64).

Schéma de la recherche

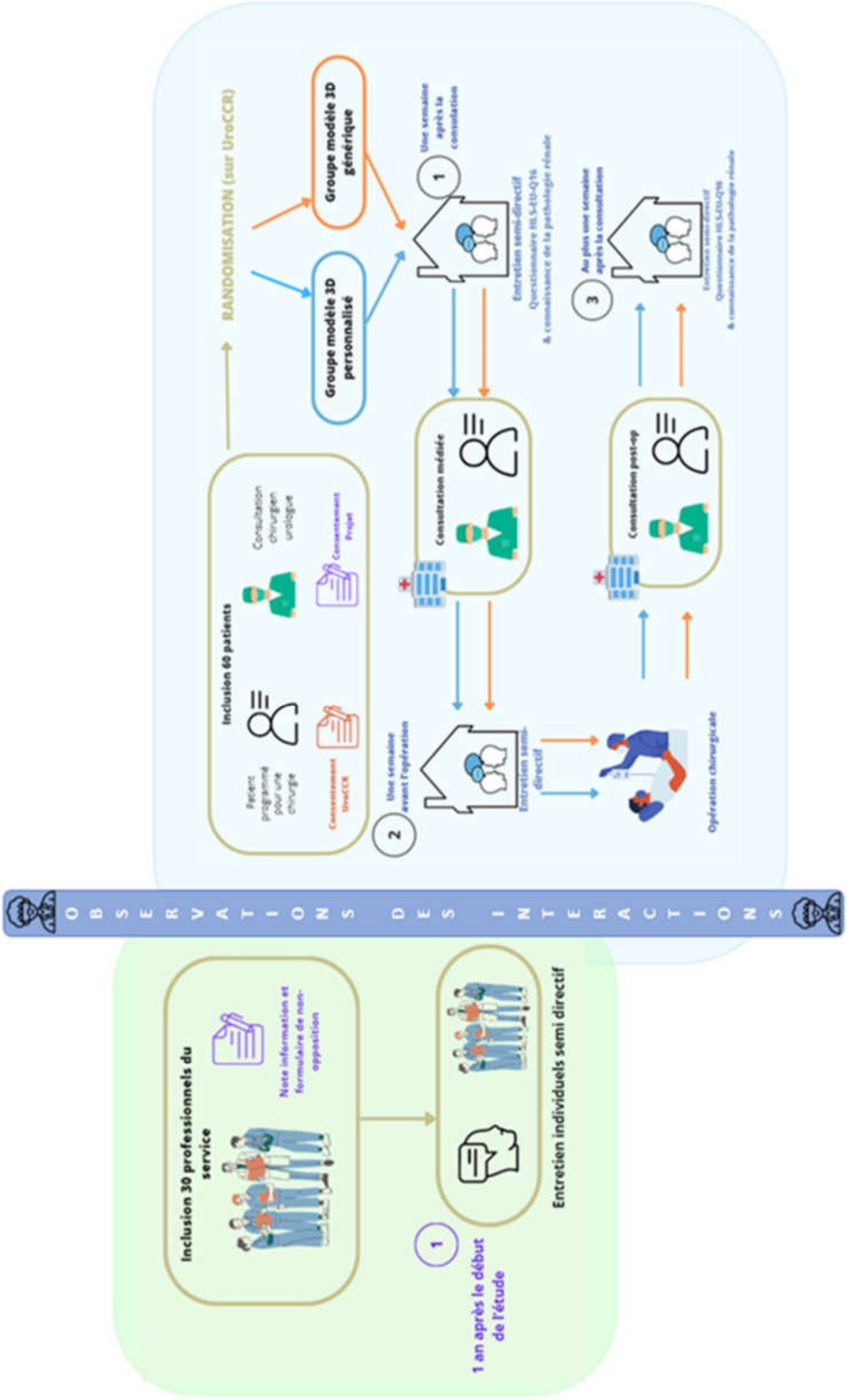
RANDOMISATION (sur UroCCR)
Groupe modèle 3D personnalisé
Groupe modèle 3D générique
Inclusion 60 patients
Patient programmé pour une chirurgie
Consultation chirurgien urologue
Consentement UroCCR
Consentement Projet
1
Une semaine après la consultation
Entretien semi-directif
Questionnaire HLS-EU-Q16
& connaissance de la pathologie rénale
Consultation médiée
2
Une semaine avant l'opération
Entretien semi-directif
Opération chirurgicale
Consultation post-op
3
Au plus une semaine après la consultation
OBSERVATIONS DES INTERACTIONS
Inclusion 30 professionnels du service
Note information et formulaire de non-opposition
1
1 an après le début de l'étude
Entretien individuels semi directif

4.3. METHODES POUR LA RANDOMISATION

La liste de randomisation est établie par la statisticienne du Centre de Méthodologie (USMR du CHU de Bordeaux) avant le début de la recherche.

Les effectifs des 2 groupes de stratégie sont équilibrés avec un ratio 1:1, sans facteur de stratification. Un document décrivant la procédure de randomisation est conservé de manière confidentielle au sein du Centre de Méthodologie et de Gestion des données.

5. CRITERES D'ÉLIGIBILITE

5.1. CRITERES D'INCLUSION

Patients

- Age ≥ 18 ans,
- Prise en charge chirurgicale programmée par néphrectomie partielle coelioscopique avec assistance robotique (1ère prise en charge pour une tumeur du rein unilatérale ou bilatérale),
- Consentement libre, éclairé et signé pour la base de données UroCCR,
- Consentement libre, éclairé et signé pour le protocole Rein-3D Personalize,
- Personne affiliée ou bénéficiaire d'un régime de sécurité sociale.

Professionnels

- Professionnel exerçant auprès des patients pris en charge dans le service d'Urologie, d'andrologie et transplantation rénale du CHU de Bordeaux depuis <u>au moins deux mois</u> avant l'implémentation des outils 3D,
- Consentement libre, éclairé et signé.

5.2. CRITERES DE NON INCLUSION

Patients

- Métastatiques au moment de l'inclusion,
- Antécédent de cancer du rein,
- Rein unique au moment de l'inclusion,
- Absence de scanner préopératoire ou scanner de mauvaise qualité ne permettant pas la réalisation d'une modélisation 3D fiable,
- Personne faisant l'objet d'une mesure de protection légale (sauvegarde de justice, tutelle ou curatelle),
- Difficultés à comprendre et à s'exprimer en français.

Professionnels

- Professionnel en stage d'une durée de moins de 6 mois,
- Professionnel sans contact avec les patients.

5.3. FAISABILITE ET MODALITES DE RECRUTEMENT

Il s'agit d'une étude monocentrique incluant des patients pris en charge dans le service d'Urologie, d'andrologie et transplantation rénale du CHU de Bordeaux. Les patients sont tous inclus dans la base de

données UroCCR et, au vu des inclusions prospectives enregistrées sur l'année 2022 dans la base de données, le potentiel d'inclusion est largement supérieur aux 4 patients/mois attendus (223 patients opérés d'une néphrectomie partielle robot assistée en 2022).

Modalites de recrutement

Patients

L'inclusion des patients sera prospective et se fera à l'issue de la consultation avec l'urologue.

Tous les patients adultes répondant aux critères d'inclusion et programmés pour une néphrectomie partielle par voie laparoscopique avec assistance robotique pour tumeur du rein se verront proposer l'étude.

L'investigateur principal donnera au participant une note d'information relative à l'étude et un formulaire de consentement.

Professionnels

Tous les professionnels répondant aux critères d'inclusion prenant en charge des patients suivis dans le service d'Urologie, d'andrologie et transplantation rénale du CHU de Bordeaux seront inclus.
Les chercheuses SHS ou l'investigateur principal présenteront eux-mêmes l'étude aux professionnels et leur donneront la note d'information relative à l'étude comportant un formulaire de consentement.

6. STRATEGIE(S)/PROCEDURE(S)/PRODUITS DE LA RECHERCHE

6.1. STRATEGIE

Patients

Lors de l'inclusion dans l'étude les patients seront randomisés en 2 bras, correspondants aux différents supports d'information utilisés lors de la consultation d'information :

1. Groupe modèle 3D imprimé personnalisé : temps d'échange avec pour support d'information le modèle tridimensionnel physique imprimé du rein à opérer
2. Groupe modèle 3D imprimé générique : temps d'échange avec pour support d'information un modèle tridimensionnel physique imprimé, issu d'une mallette de huit modèles, et correspondant au plus près à la situation

Population professionnels

Pas d'intervention spécifique

6.2. PROCEDURE EXPERIMENTALE ET DE COMPARAISON

6.2.1 Groupe Modele 3D Imprime Personnalise

Pour les patients randomisés dans le groupe « personnalisé », une modélisation 3D du rein et de la tumeur sera réalisée par le chirurgien, à partir du scanner préopératoire, à l'aide du logiciel Synapse 3D (Fujifilm). L'impression de ce modèle sera ensuite réalisée à l'aide de l'imprimante Stratasys acquise en partenariat avec l'IUT de Bordeaux et située sur le site de l'IUT (envoi des fichiers 3D (.stl) anonymisés par le logiciel à l'ingénieur de l'IUT en charge des impressions). Le délai d'impression du modèle 3D est d'environ 15 jours après la réalisation des scanners.

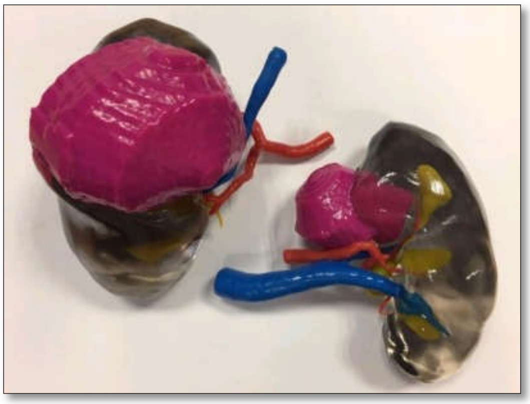

Exemple de modélisation 3D imprimée

Pour le groupe 1, le modèle personnalisé imprimé en 3D est présenté au patient par le chirurgien lors d'une consultation dédiée entre J-30 et J-7 avant l'opération, accompagné de commentaires anatomiques et de stratégies chirurgicales simples.

Pendant cette visite le patient pourra alors prendre en main et manipuler le modèle personnalisé.

6.2.2 GROUPE MODELE 3D IMPRIME GENERIQUE

Pour les patients randomisés dans le groupe « générique », le chirurgien dispose d'une mallette composée de 8 modèles de rein imprimés en 3D (réalisée à l'aide de l'imprimante Stratasys acquise en partenariat avec l'IUT de Bordeaux et située sur le site de l'IUT).

Ces 8 modèles permettent de couvrir :

- Les différentes localisations : supérieure, équatoriale et inférieure ; antérieure et postérieure ; bord externe et bord interne,
- Les tumeurs hilaires,
- La caractéristique endophytique ou exophytique,
- Les différentes tailles : cT1a, cT1b et cT2,
- La vascularisation modale ou la présence d'une 2ème artère.

Le chirurgien sélectionnera parmi les modèles génériques celui se rapprochant le plus de la situation du patient en fonction de la taille et de la localisation de la tumeur du rein. Le modèle générique imprimé en 3D, sera alors présenté au patient par le chirurgien lors d'une consultation dédiée entre J-30 et J-7 avant l'opération, accompagné de commentaires anatomiques et de stratégies chirurgicales simples. Le patient pourra alors prendre en main et manipuler le modèle générique.

7. DEROULEMENT DE LA RECHERCHE

7.1 CALENDRIER DE LA RECHERCHE

Patients

- Durée de la période d'inclusion - 18 mois, -
  Durée de la participation – entre 3 et 6 mois.

Population professionnels

Participation tout au long de l'étude, dans le cadre de l'observation des parcours des patients. Durée de participation pour les professionnels : 24 mois.

Durée totale de la recherche : 38 mois (inclusion, suivi, analyse et valorisation).

7.2 TABLEAU RECAPITULATIF DU SUIVI PARTICIPANT

Patients

	Inclusion (première consultation avec le chirurgien) T0	Période préopératoire T1	Consultation d'échange et d'information T2	Seconde période préopératoire T3	Chirurgie T4	Consultation post-opératoire T5	Période post consultation post opératoire T6
	J0-180 à J0-30	T0+15j max	J0-30j à J0-7j	Au plus T2 +15j	J0	J0+15j à J0 +45j	T5+15max
Information et recueil du consentement (R)	✓						
Informations concernant la consultation (patient accompagné par qui, durée de la consultation) (R)	✓		✓			✓	
Randomisation (R)	✓						
Scanner abdominopelvien avec injection de produit de contraste, si nécessaire (S) *	✓						
Modélisation 3D (R)		✓					
Impression 3D (R)		✓					
Informations préopératoires (R)			✓				
Chirurgie (S)					✓		
HLSEU-Q16 – mesure du niveau de littératie en santé – 10' (R)		✓					✓
Questionnaire connaissances – 10' (R)		✓		✓			✓
Questionnaire de satisfaction – 5' (R)							✓
Entretien semi-directif réalisé par un chercheur en SHS après les questionnaires		✓		✓			✓

(R) : recherche
(S) : soins courants
* 15 jours devant être pris en compte pour l'impression et la transmission du modèle 3D imprimé, l'imagerie doit avoir été réalisée au moins 15 jours avant la consultation d'information.

- Schéma temporel du suivi patient

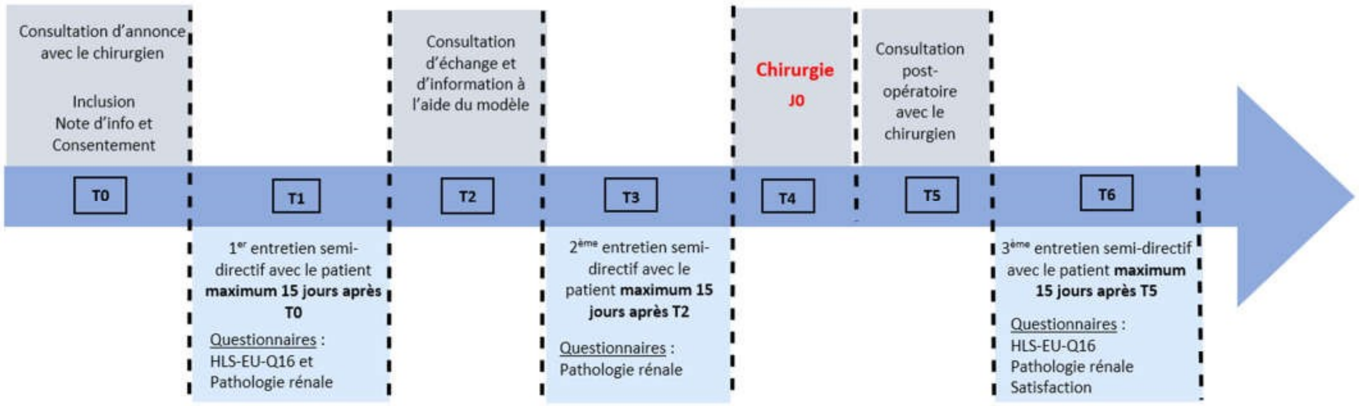

Professionnels

	Présentation de l'étude à l'ensemble de l'équipe - T0		Entretien avec professionnel
	Lors de la mise en place de l'étude	Entre la mise en place et 1 an après la première inclusion	1 an après la première inclusion de patient
Information	✓		
Recueil du consentement (R)		✓	
Entretien semi-directif réalisé par un chercheur en SHS (R)			✓

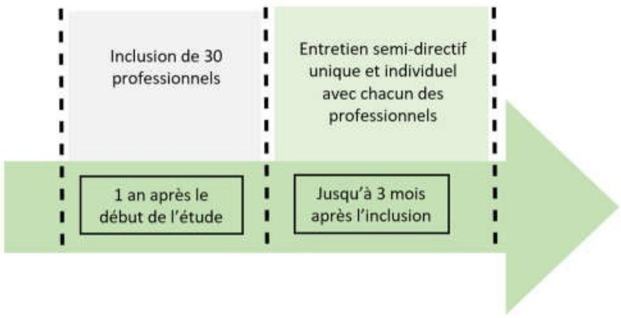

7.3 VISITE D'INCLUSION (T0)

7.3.1 RECUEIL DU CONSENTEMENT

Lors de la visite d'inclusion, le médecin investigateur informe le participant et répond à toutes ses questions concernant l'objectif, la nature des contraintes, les risques prévisibles et les bénéfices attendus de la recherche. Il précise également les droits du participant dans le cadre d'une recherche et vérifie les critères d'éligibilité.

Un exemplaire de chaque note d'information (Rein-3D PERSONALIZE et UroCCR) et du formulaire de consentement est alors remis au participant par le médecin investigateur. Après cette séance d'information, le participant dispose d'un délai de réflexion. Si le participant donne son accord de participation, ce dernier et l'investigateur inscrivent leurs noms et prénoms en clair, datent et signent les

deux formulaires de consentement en deux exemplaires originaux (pour la participation au projet Rein-3D PERSONALIZE et pour la collecte les données nécessaires à l'analyse dans la base de données UroCCR) Ceux-ci doivent être signés avant la réalisation de tout examen clinique au paraclinique nécessité par la recherche.

Les différents exemplaires de la note d'information et du formulaire de consentement sont alors répartis comme suit :

- Un exemplaire original de chaque note d'information et de chaque consentement signé est remis au participant.
- Les autres exemplaires originaux sont conservés par l'investigateur (même en cas de déménagement du participant pendant la durée de la recherche) dans un lieu sûr inaccessible à des tiers.

7.3.2 Deroulement de la visite

Patients

La visite d'inclusion est assurée par l'investigateur. Avant tout examen lié à la recherche, l'investigateur recueille le consentement libre et éclairé du patient (ou de son représentant légal le cas échéant).

Les patients inclus sont reçus dans le service d'urologie, dans le cadre de la prise en charge de leur pathologie. La visite d'inclusion est assurée par l'investigateur. Un membre de l'équipe de recherche SHS pourra participer à la visite en tant qu'observateur.

Professionnels

Une réunion collective de présentation de l'étude est effectuée un mois avant le début des entretiens par l'investigateur et l'équipe de recherche SHS qui présentent l'étude sur des temps formels ou informels institués dans le service auprès des professionnels.

L'équipe de recherche SHS agira sous la responsabilité de l'investigateur.

Les chercheuses SHS informent et répondent à toutes les questions concernant l'objectif, la nature des contraintes, les risques prévisibles et les bénéfices attendus de la recherche. Elles précisent également la possibilité pour le participant de se retirer à tout moment de la recherche, et vérifient les critères d'éligibilité. Un exemplaire de la note d'information (avec les coordonnées de l'équipe de recherche SHS et de l'attachée de recherche clinique du projet Rein 3D Print PERSONALIZE) et du formulaire de consentement est alors remis au professionnel par l'équipe de recherche. L'équipe de recherche recueille ensuite le consentement de participation.

- Un exemplaire original de la note d'information et du consentement signé est remis au participant.
- L'autre exemplaire original sera conservé par l'investigateur (même en cas de déménagement du participant pendant la durée de la recherche) dans un lieu sûr inaccessible à des tiers.

7.3.3 Visite/demarche de randomisation

La randomisation des patients sera réalisée juste après la consultation d'inclusion. Elle s'effectuera directement sur l'interface web de la base de données UroCCR.

Lorsqu'un investigateur souhaitera effectuer la randomisation, après vérification préalable de l'éligibilité du participant, il se connectera avec ses codes sur le site Internet: http:uroccr.fr. Il complètera l'onglet « Recherche / Rein-3D PERSONALIZE » et confirmera tous les critères d'éligibilité du patient. Après validation du contenu, l'inclusion et la randomisation seront effectuées. L'interface communiquera immédiatement à l'investigateur le numéro unique du patient dans la recherche et le résultat de la

randomisation. Le résultat de la randomisation ne sera communiqué au patient qu'à la Consultation d'échange et d'information (T2).

7.4 VISITES DE SUIVI

7.4.1 PERIODE PREOPERATOIRE (T1)

Les patients sont vus, entre la visite d'inclusion et la consultation d'information, par un membre de l'équipe de recherche SHS à l'hôpital ou à leur domicile selon leurs préférences au maximum à T0 +15j. Ce temps d'échange correspond à un entretien en face à face durant lequel seront abordés :

- Recueil de l'expérience du patient depuis les premiers symptômes ayant conduit à la visite au service d'urologie,
- Recueil de données socio-démographiques,
- Recueil concernant les échanges avec l'entourage au sujet de la maladie,
- Questionnaire de littératie HLSEU-Q16 recueilli via une tablette sur UroConnect,
- Questionnaire de compréhension de l'anatomie rénale et des enjeux chirurgicaux via une tablette sur UroConnect.

7.4.2 CONSULTATION D'ECHANGE ET D'INFORMATION (T2)

Les patients sont vus entre 30 jours et 7 jours avant l'opération par un membre de l'équipe chirurgicale. Ce temps d'échange a pour objectif de permettre au patient d'exprimer ses interrogations, faire part de ses doutes ou incompréhensions en lien avec sa situation médicale et l'intervention programmée. Elle pourra déboucher sur des explications sur l'anatomie et sur les stratégies chirurgicales simples par le membre de l'équipe chirurgicale participant à cette entrevue.
Les deux groupes interventionnels (Groupe Modèle 3D Imprimé personnalisé et Groupe Modèle 3D Imprimé générique) se voient présenter le modèle comme support de discussion.
Les explications données aux patients ne sont pas standardisées dans le cadre de l'étude afin de se rapprocher de la pratique habituelle.
La durée de cette visite ainsi que la présence ou non d'un accompagnant seront recueillis.
Un membre de l'équipe de recherche SHS pourra participer à la visite en tant qu'observateur.

7.4.3 SECONDE PERIODE PREOPERATOIRE (T3)

Les patients sont vus entre la consultation d'information et l'opération par un membre de l'équipe de recherche SHS à l'hôpital ou à leur domicile selon leurs préférences au maximum à T2 +15j. Ce temps d'échange correspond à un entretien en face à face durant lequel seront abordés les éléments suivants :

- Recueil de l'expérience du patient depuis le dernier entretien,
- Recueil concernant les échanges avec l'entourage au sujet de la maladie,
- Questionnaire de compréhension de l'anatomie rénale et des enjeux chirurgicaux avec tablette sur UroConnect.

7.4.4 CHIRURGIE (T4)

Sans spécificité pour l'étude mais, comme pour tout patient inclus dans UroCCR, les caractéristiques et le déroulé technique de l'intervention chirurgicale seront collectés. Il n'y aura, du fait de l'étude, aucune modification de la technique chirurgicale de néphrectomie partielle robot-assistée telle qu'envisagée par le chirurgien.

7.4.5 Consultation post-operatoire (T5)

Le bilan post-opératoire sera collecté. Il n'y aura, du fait de l'étude, aucune modification de la consultation post-opératoire si ce n'est la présence potentielle d'un membre de l'équipe de recherche SHS pendant la consultation.

7.5 VISITE DE FIN DE LA RECHERCHE (T6)

Patients

Le recueil de données de la recherche se termine au plus tard 15 jours après la dernière consultation postopératoire.

Les patients seront vus par un membre de l'équipe de recherche SHS à l'hôpital ou à leur domicile, selon leurs préférences. Ce temps d'échange correspond à un entretien en face à face durant lequel seront abordés les éléments ci-dessous :

- Recueil de l'expérience du patient depuis le dernier entretien,
- Recueil concernant les échanges avec l'entourage au sujet de la maladie,
- Questionnaire de littératie HLSEU-Q16 via une tablette sur UroConnect,
- Questionnaire de compréhension de l'anatomie rénale et des enjeux chirurgicaux via une tablette sur UroConnect,
- Questionnaire de satisfaction via une tablette sur UroConnect.

Professionnels

Un entretien permettant le recueil de l'expérience du professionnel suite à l'implémentation des outils 3D Print dans sa relation avec les patients et leurs entourages sera réalisé au sein du service d'urologie au plus tard un an après l'inclusion du premier patient.

7.6 REGLES D'ARRET

7.6.1 Arret de la participation d'une personne a la recherche

Le participant qui souhaite abandonner ou retirer son consentement de participation à la recherche (comme il est en droit de le faire à tout moment) n'est plus suivi dans le cadre du protocole, mais doit faire l'objet de la meilleure prise en charge possible compte tenu de son état de santé et de l'état des connaissances du moment.

Un abandon est une décision d'un participant inclus de faire valoir son droit d'interrompre sa participation à une recherche, à tout moment au cours du suivi, sans qu'il n'encoure aucun préjudice de ce fait et sans avoir à se justifier.

Un retrait de consentement est une décision d'un participant de revenir sur sa décision de participer à une recherche et de faire valoir son droit d'annuler son consentement éclairé, à tout moment au cours du suivi et sans qu'il n'encoure aucun préjudice de ce fait et sans avoir à se justifier.

L'investigateur doit identifier la cause de l'abandon/du retrait et évalue s'il est possible de recueillir la variable sur laquelle porte le critère de jugement principal au moment de l'abandon/du retrait. Les abandons/retraits doivent être notifiés rapidement au centre investigateur coordonnateur, au promoteur et au centre de méthodologie et de gestion des données. Leurs raisons et leur date doivent être documentées dans le cahier d'observation et dans le dossier médical du participant.

7.6.2 Arret de la recherche

Fin de la recherche ou arrêt prévu de la recherche : terme de la participation de la dernière personne qui se prête à la recherche aussi appelé dernière visite du dernier participant inclus dans la recherche.

Lorsque la recherche a atteint son terme prévu (arrêt prévu), la fin de la recherche doit être déclarée à l'ANSM et au CPP dans un délai de 90 jours.

Arrêt anticipé de la recherche : la recherche clinique est arrêtée (définitivement) de façon anticipée. C'est le cas, notamment, lorsque le promoteur décide :

- de ne pas commencer la recherche malgré l'obtention de l'avis favorable d'un CPP ;
- de ne pas reprendre la recherche après l'avoir interrompu temporairement ou après sa suspension par l'ANSM.

Lorsque la recherche est arrêtée (définitivement) de façon anticipée, la fin de la recherche doit être déclarée à l'ANSM dans un délai de 15 jours en indiquant les raisons qui motivent cet arrêt.

Arrêt temporaire de la recherche : l'arrêt temporaire d'une recherche clinique consiste en :

- l'arrêt de l'inclusion de nouvelles personnes dans cette recherche;
- et/ou l'arrêt de la pratique des actes prévus par le protocole de la recherche.

Toute décision du promoteur d'interrompre temporairement la recherche doit faire l'objet d'une information immédiate à l'ANSM et au CPP concerné et dans un second temps et dans un délai maximum de 15 jours calendaires suivant la date de cette interruption, d'une demande d'autorisation de modification substantielle concernant cet arrêt temporaire soumise à l'ANSM et d'une demande d'avis au CPP concerné.

7.7 DEVIATIONS AU PROTOCOLE

Les déviations peuvent concerner tous les aspects d'un protocole de recherche : processus d'inclusion, suivi, mesure des critères de jugement, traitements. Toutes doivent être documentées par l'investigateur et discutées en Conseil Scientifique.

Seuls les abandons entraînent un arrêt du suivi. Même en cas de déviation au protocole, le suivi du participant doit être mené jusqu'au terme prévu dans le protocole.

7.7.1 Arret premature et definitif de la procedure de la recherche

Les participants en arrêt prématuré continuent à être suivis comme prévu par le protocole. En aucun cas, le suivi prévu ne doit être modifié.

Le participant doit faire l'objet de la meilleure prise en charge possible compte tenu de son état de santé et de l'état des connaissances du moment.

7.7.2 Participant perdu de vue

Un participant est considéré comme perdu de vue quand il arrête le suivi prévu dans le cadre du protocole sans raison connue de l'investigateur, de sorte que le recueil des données ne peut pas être effectué comme prévu.

Les participants perdus de vue doivent faire l'objet d'une recherche active de la part de l'investigateur.

7.7.3 Participant inclus a tort

Un participant est considéré comme inclus à tort lorsqu'il a effectivement été inclus dans la recherche alors qu'il ne vérifiait pas tous les critères d'éligibilité. Les participants inclus à tort doivent faire l'objet d'une discussion en Conseil Scientifique. Ils doivent continuer à être suivis comme prévu par le protocole jusqu'à ce qu'une décision soit prise par le Conseil Scientifique.

7.8 PARTICIPATION SIMULTANEE A D'AUTRES RECHERCHES, PERIODE D'EXCLUSION, INDEMNISATION ET INSCRIPTION AU FICHIER VRB

En dehors des études ACCURATE et ANXIETY la personne peut participer simultanément à une autre recherche.
Aucune indemnisation ne sera perçue par les participants pendant la durée de cette recherche.

8. GESTION DES EVENEMENTS INDESIRABLES / EFFETS INDESIRABLES / INCIDENTS

Les évènements indésirables / effets indésirables / incidents seront à déclarer aux différents circuits de vigilances sanitaires applicables à chaque produit ou pratique concernée (vigilance du soin, pharmacovigilance, hémovigilance, cosmétovigilance…) en conformité avec la règlementation en vigueur.

Les déclarants doivent spécifier que le patient est inclus dans un essai clinique et identifier précisément l'essai clinique concerné.

Si l'investigateur a connaissance d'une atteinte à la sécurité des patients dans le cadre de la recherche, il doit en informer sans délai le promoteur.

9. ASPECTS STATISTIQUES

9.1 TAILLE D'ETUDE

Patients
L'approche de l'étude est principalement de type anthropologique par essence systémique, elle s'appuie sur une approche mixte avec des observations, des entretiens et des questionnaires. Pour pouvoir allier, dans le cadre du recueil puis de l'analyse, les approches qualitatives et quantitatives il a été décidé de construire une étude avec 60 patients répartis en 2 groupes randomisés de 30 patients. Les patients qui auront des données non exploitables seront exclus des analyses et remplacés avec un maximum d'inclusion total de 68 patients.

Professionnels
De même il a été choisi de réaliser des entretiens auprès de 30 professionnels, répondant aux critères d'inclusion, et permettant de couvrir l'hétérogénéité en termes de profession et d'ancienneté. Ceci permettra de mener correctement les entretiens dans le temps imparti et avoir une représentation au sein de la cohorte de l'hétérogénéité professionnelle et d'expérience.

9.2 METHODES STATISTIQUES EMPLOYEES

9.2.1 Strategie d'analyse

Les données seront analysées par l'équipe de chercheuses en SHS pour assurer l'analyse croisée des données qualitatives et quantitatives.
L'analyse principale sera effectuée par sous-traitement pour les patients, c'est à dire que les données de tous les participants doivent être analysées au regard du modèle 3D imprimé réellement administré, même si la randomisation en a décidé autrement, sur données disponibles.

Le schéma ci-dessous présente les différentes phases de la recherche par méthode mixte qui est proposée ici. De nombreux outils de recueils sont utilisés dans le cadre de cette étude pour pouvoir étudier les effets

de l'utilisation d'un modèle personnalisé de rein imprimé en 3D versus un modèle générique de rein imprimé en 3D comme outil de médiation sur l'expérience des patients et leurs interactions avec les professionnels. Cette approche par triangulation (21) nécessite des analyses spécifiques des données obtenues à partir des différents outils de recueil, associées à des interprétations croisées pour répondre aux différents objectifs.

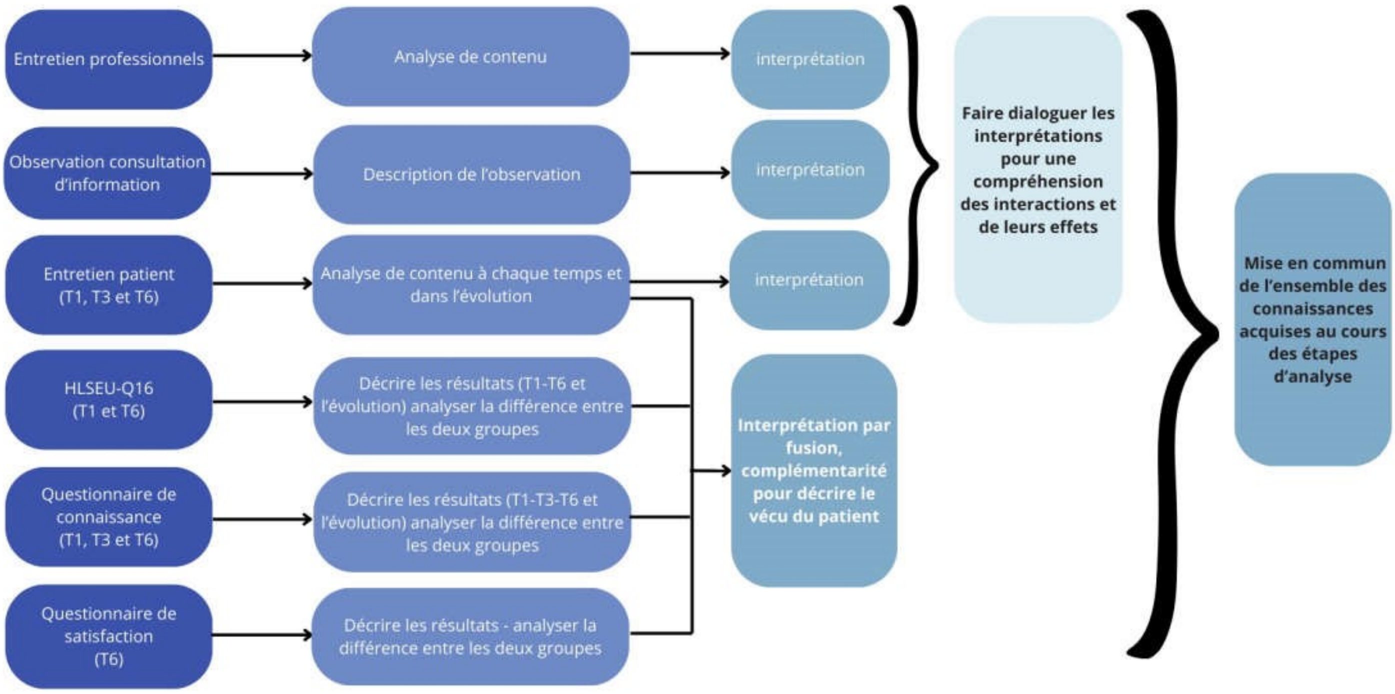

Une analyse descriptive sera réalisée de manière globale et par groupe de modèle 3D.
Une analyse de comparaison entre les groupes de randomisation sera réalisée, systématiquement sans ajustement et avec ajustement sur les facteurs pronostiques initiaux dont la répartition pourrait être, malgré la randomisation, déséquilibrée.

9.2.2 Patients inclus dans l'analyse

Ne pourront être exclus de l'analyse que les patients et professionnels qui présentent au moins une des conditions suivantes :

- Patients inclus à tort pour consentement non signé ;
- Patients ou professionnels inclus à tort pour critère(s) majeur(s) d'éligibilité non respecté(s) ;
- Patients ou professionnels ayant retiré leur consentement.

Cette décision d'exclusion sera prise par le Conseil Scientifique en insu du groupe de randomisation et de l'évolution du patient après l'inclusion.
En dehors de ces exclusions, les patients ou professionnels décédés, perdus de vue ou ayant abandonné la recherche seront tous inclus dans l'analyse.

9.2.3 Risque de premiere espece

Les analyses de comparaison des données statistiques seront effectuées au risque d'erreur global de = 5%.

9.2.4 METHODES STATISTIQUES DESCRIPTIVES

Pour les résultats des questionnaires utilisés :

- Le nombre ainsi que le pourcentage de participants (patients et professionnels) avec des données manquantes seront décrits pour chaque variable d'intérêt. La raison des données manquantes sera documentée autant que possible afin d'interpréter les résultats.
- Les variables qualitatives seront décrites en termes d'effectifs, de pourcentages et d'intervalles de confiance à 95% selon la loi binomiale exacte.
- Les variables quantitatives seront décrites en termes d'effectifs, moyennes, écarts-types, médianes, étendues et étendues interquartiles.

Pour les observations des consultations, des données systématiquement recueillies seront décrites (durée des différentes visites, délais entre les visites, caractéristiques du/des professionnels …).

Une analyse des entretiens mènera à une approche statistique de contenu, permettant de décrire, entre autres, les éléments de compréhension.

9.2.5 METHODES STATISTIQUES COMPARATIVES

Les distributions des variables qualitatives seront comparées entre les groupes par des tests du $\chi 2$, ou du $\chi 2$ corrigé, ou de Fisher exact, selon les valeurs des effectifs attendus sous l'hypothèse d'indépendance.
Les distributions des variables quantitatives seront comparées entre les groupes par le test de Student si les conditions de validité du test sont respectées (distribution normale, variances homogènes). Si les variances sont inégales entre les deux groupes, on utilisera un test de Student pour variances inégales et si la distribution n'est pas normale, on utilisera un test de Wilcoxon.

9.2.6 METHODES D'ANALYSE DE DONNEES QUALITATIVES

Les entretiens ainsi que les recueils d'observations sont sources de contenus importants à analyser pour pouvoir étudier les thèmes abordés, la ressemblance et l'hétérogénéité entre enquêtés mais aussi l'évolution de ces derniers au cours des différentes phases de l'étude, tout en tenant compte pour les échantillons de patients de l'appartenance à l'un ou l'autre des groupes pour en voir les différences.

9.2.7 LOGICIELS UTILISES

Les analyses seront réalisées avec le logiciel R (version n°4.2.3 ou ultérieures) et le logiciel Nvivo (version 9.4 ou ultérieures).

9.3 PLAN D'ANALYSE

9.3.1 DESCRIPTION DES INCLUSIONS, DES DEVIATIONS ET DU SUIVI Les

éléments suivants seront présentés :

- Vérification des critères d'éligibilité,
- Le diagramme de flux de l'étude suivant les recommandations CONSORT,
- Une description des causes de décès, d'abandon, des patients et des professionnels perdus de vue ou ayant abandonné la recherche, des patients n'ayant pas été opérés,
- Les déviations au protocole (concernant notamment les délais),

- Visites de suivi réalisées : nombre de patients ayant réalisé chacune des visites de suivi.

9.3.2 Caracteristiques des patients a l'inclusion

Les patients seront décrits selon les variables suivantes :

- caractéristiques démographiques,
- caractéristiques cliniques, - caractéristiques biologiques,
- caractéristiques de la stratégie.

9.3.3 Analyse de l'objectif principal

L'objectif principal est d'étudier les effets de l'utilisation d'un modèle personnalisé de rein imprimé en 3D versus un modèle générique de rein imprimé en 3D comme outil de médiation, tout au long du parcours, sur l'expérience des patients et leurs interactions avec les professionnels, avant et après néphrectomie partielle.

L'analyse de l'objectif principal s'effectuera en s'appuyant sur le croisement des éléments issus des différents outils de recueils et dépendra des contenus des entretiens ainsi que des observations. L'analyse des verbatims se nourrira des données d'observation et des données issues des questionnaires. Une analyse par triangulation des données sera donc effectuée globalement puis par groupe pour en étudier les différences.

Une analyse descriptive sera réalisée de manière globale et par groupe de stratégie pour les patients sur l'ensemble des données issues des questionnaires, des données socio-démographiques ainsi que des données caractérisant la tumeur. La description des données socio-démographiques de chacun des deux groupes de patients (groupe modèle 3D générique / groupe modèle 3D spécifique) sera réalisée : en indiquant les fréquences (ex : sexe, profession…), les moyennes et écart-types (ex : âge, délai de maladie…) permettant de comparer la répartition des deux groupes.

Une analyse de comparaison entre les groupes de types de modèles 3D sera réalisée, systématiquement sur les données issues des questionnaires et les données socio-démographiques.
Les données issues des verbatims d'entretiens seront analysées afin de pouvoir étudier les expériences de vie singulières des patients ainsi que les éléments communs entre patients et les hétérogénéités entre groupe.

Les observations et entretiens sont des modes de recueil ne menant pas automatiquement à une analyse statistique des données. L'analyse statistique ne portera que sur une partie des données recueillies, qualitatives et quantitatives.

La description de l'expérience des patients sera réalisée à partir de l'analyse des contenus des entretiens de manière transversale pour chaque temps mais aussi selon une analyse longitudinale prenant en considération les 3 temps (T, T3 et T6) et en spécifiant le groupe d'appartenance du patient.

9.3.4 Analyse des objectifs secondaires

La description des interactions entre les professionnels et les patients, en précisant les termes employés ainsi que leurs fréquences sera effectuée via l'analyse et la synthèse des informations recueillies via les carnets d'observations. Cette analyse permettra de répondre aux objectifs secondaires 1 et 2. Des éléments

complémentaires concernant les interactions patients et entourage pourront être issus des entretiens auprès des patients et viendront enrichir la réponse à l'objectif 2.

Pour répondre au troisième objectif secondaire, une synthèse de toutes les informations recueillies dans le cadre des observations mais aussi des entretiens auprès des patients et des professionnels permettra de retracer l'usage qui est fait des modèles 3D et de comprendre les utilisateurs et les circonstances d'utilisation.

L'évolution de la compréhension de la maladie et de la chirurgie sera étudiée par l'analyse des contenus des entretiens et du questionnaire de compréhension de l'anatomie rénale et des enjeux chirurgicaux avec tablette sur UroConnect. Les contenus seront comparés entre les deux groupes.

Une analyse descriptive de la distribution des scores obtenus par les patients pour le questionnaire littératie HLS-EU16 sera réalisée dans chacun des groupes pour les temps 1 et 6. Une analyse de comparaison entre le temps 1 et le temps 6 sera réalisée, pour chaque groupe, par un test de Student apparié, après vérification de l'homogénéité des variances, dans le cas contraire des tests non-paramétriques seront utilisés. Enfin les évolutions entre les deux groupes (HLSEU-Q16 au temps T6 - HLSEU-Q16 au temps T1) seront comparées par un test de Student.
Une seconde analyse sera réalisée en utilisant le classement du score de littératie en trois niveaux comme indiqué dans la partie 4.2, les données seront décrites pour chaque temps puis une comparaison entre les temps sera réalisée par un test du Chi deux appariés.

Une analyse thématique de contenu des entretiens réalisés auprès des professionnels sera une réalisée.

10. SURVEILLANCE DE LA RECHERCHE

10.1 CONSEIL SCIENTIFIQUE

10.1.1 COMPOSITION

Il est composé des personnes suivantes : Dr Gaëlle MARGUE (Présidente), Pr Jean-Christophe BERNHARD (Responsable scientifique), Pr Laura RICHERT (Méthodologiste), Roxane COUËRON (Biostatisticienne), Marthe-Aline JUTAND, (Chercheuse en sciences de l'éducation), Hélène HOARAU (Anthropologue), Sarah MASANET (Doctorante en sciences de l'éducation et de la formation), Solène RICARD (Cheffe de Projet), Manon JAFFREDO (Cheffe de projet), Clémence MORICE (Attachée de recherche clinique coordinatrice) et le(la) représentant(e) du promoteur.

10.1.2 RYTHME DES REUNIONS

Le Conseil Scientifique de la recherche se réunit selon les besoins de l'étude et au moins une fois par an.

10.1.3 ROLE

- Il a pour mission de prendre toute décision importante à la demande de l'investigateur coordonnateur concernant la bonne marche de la recherche et le respect du protocole.
- Il vérifie le respect de l'éthique.
- Il s'informe auprès du Centre de Méthodologie et de Gestion des données et du centre investigateur coordonnateur de la recherche de l'état d'avancement de la recherche, des problèmes éventuels et des résultats disponibles.

- Il décide de toute modification pertinente du protocole nécessaire à la poursuite de la recherche, notamment :
    - les mesures permettant de faciliter le recrutement dans la recherche,
    - les modifications des documents de la recherche (protocole et des documents d'information et de recueil de consentement) avant leur présentation au CPP,
    - les mesures qui assurent aux personnes participant à la recherche la meilleure sécurité, la discussion des résultats et la stratégie de publication de ces résultats.
- Le Conseil Scientifique peut proposer de prolonger ou d'interrompre la recherche en cas de rythme d'inclusion trop lent, d'un trop grand nombre de perdus de vue, de violations majeures du protocole ou bien pour des raisons médicales et/ou administratives. Il précise les modalités éventuelles du suivi prolongé des participants inclus dans la recherche.
- A l'issue de la réunion, la présidente du Conseil Scientifique doit informer le promoteur des décisions arrêtées. Les décisions concernant une modification majeure ou une modification de budget doivent être approuvées par le promoteur.

10.2 COMITE INDEPENDANT DE SURVEILLANCE

Cette étude ne nécessite pas la mise en place d'un comité indépendant de surveillance du fait de l'absence de tout traitement pouvant entraîner un arrêt prématuré de la recherche et la nature des procédures à l'étude n'entrainant aucun risque notable pour les patients.

11. GESTION ET TRAITEMENT DES DONNEES ET DOCUMENTS SOURCES

11.1 DONNEES ET DOCUMENTS SOURCES

Les données sources correspondent à l'ensemble des informations figurant dans des documents originaux, ou dans des copies authentifiées de ces documents, relatif aux examens cliniques, aux observations ou à d'autres activités menées dans le cadre d'une recherche et nécessaires à la reconstitution et à l'évaluation de la recherche. Les documents dans lesquels les données sources sont enregistrées sont appelés documents sources.

Besoin des données UroCCR

- Caractéristiques socio-démographiques du patient (âge, sexe),
- Taille de la tumeur,
- Score de complexité tumorale – RENAL,
- Antécédents de cancer familial,
- Diagnostic de cancer du rein,
- Distance hôpital domicile,
- Durée de l'opération,
- Antécédent chirurgical avec anesthésie générale, Présence d'un accompagnant lors de la consultation.

11.2 CONSIGNES POUR LE RECUEIL DES DONNEES

Toutes les informations requises par le protocole doivent être consignées dans le dossier médical. Les données devront être recueillies au fur et à mesure qu'elles sont obtenues et transcrites de façon nette et lisible. Les données médicales seront collectées dans la base UroCCR.

11.3 GESTION ET CIRCUIT DES DONNEES

11.3.1 Logiciel de gestion de donnees

11.3.1.1 Logiciel utilise

eCRF pour les données quantitatives : le logiciel utilisé pour la gestion des données est un eCRF accessible à l'adresse suivante : https://uroccr.fr

La maintenance et le développement informatiques de l'eCRF sont gérés par le CREDIM (Centre de Recherche et Développement en Informatique Médicale) qui est une plateforme informatique créée au sein de l'Université de Bordeaux.

UroConnect : La maintenance et le développement de l'application UroConnect sont assurés par la société Resilience basée en France (https://www.resilience.care/).

Modélisation 3D : Le logiciel utilisé pour la modélisation 3D est Synapse 3D de Fujifilm.

Nvivo Pro pour les données qualitatives : Les données seront retranscrites dans un document de traitement de texte (de type .docx) ou directement au sein du logiciel d'aide à l'analyse qualitative Nvivo Pro 13®, puis seront analysées via le logiciel Nvivo Pro 13®.

11.3.1.2 Hebergement des donnees

Le CREDIM : Le système de gestion de base de données utilisé est Microsoft SQL server.
UroConnect : Les données collectées par UroConnect sont stockées dans un service hébergé en France (fournisseur Ecritel).

Modélisation 3D : Les modélisations 3D sont hébergées sur les serveurs ou sur l'ordinateur stand alone ayant le logiciel Synapse 3D, sur le serveur du CHU de Bordeaux (NextCloud) puis sur l'ordinateur du technicien du TechnoShop en charge de réaliser l'impression 3D.

Logiciel de saisie des données qualitatives :
Les documents suivants relatifs à cette recherche sont archivés conformément aux Bonnes Pratiques Cliniques et à la réglementation en vigueur :

- Le protocole et les modifications éventuelles au protocole : par le promoteur et l'équipe de recherche SHS,
- Tous les autres documents et courriers relatifs à la recherche par le promoteur : l'équipe de recherche SHS.
- Les carnets de notes : par l'équipe de recherche SHS au sein du laboratoire CeDS.
- Les autres données-sources et données retranscrites seront conservées sur les ordinateurs de l'équipe de recherche SHS jusqu'à 5 ans après la fin du projet ANR-21-RHUS-0015, soit 2032.

11.3.1.3 Securite des donnees

UroConnect : les données collectées par UroConnect sont stockées dans un environnement HDS. eCRF pour les données quantitatives : le serveur se trouve dans une pièce dédiée, sans fenêtre. L'entrée dans le local sécurisé se fait au moyen d'un badge. Les portes du service sont sécurisées et fermées à clé le soir. Aucun ordinateur n'est en accès libre, l'authentification sur domaine est obligatoire.

La gestion des droits d'accès aux données recueillis par l'eCRF est gérée par le CREDIM. Seuls les gestionnaires de la base, l'équipe projet et les auditeurs ont des droits d'accès direct à la base de données.

L'USMR transmettra au CREDIM la liste des personnes devant avoir accès aux données. Les statisticiens et les DM auront donc accès aux données en lecture uniquement.

Données qualitatives (anonymisées) : toutes les données anonymisées (de format audio ou texte ou Nvivo) seront stockées sur un disque externe localisé au CeDS mis sous clef pendant la durée de l'étude. Les données seront ensuite stockées en archivage sur le réseau de l'Université de Bordeaux (système CIRRUS).

11.3.2 Saisie des donnees

UroCCR : La saisie des données est sous la responsabilité de l'investigateur du centre dans l'eCRF. Toute autre personne que l'investigateur effectuant la saisie dans l'eCRF doit être préalablement formée et déléguée par l'investigateur pour le faire.

Les questionnaires seront recueillis soit sur papier, soit directement via l'outil numérique UroCONNECT qui diffusera, sur un timing adapté, les différents questionnaires définis par le protocole par un envoi automatique aux patients.

Les données sources issues des observations (prise de notes dans un carnet) et des entretiens (support audionumérique), seront retranscrites, saisies, codées et encodées exclusivement par l'équipe de recherche SHS.
Ainsi, les données seront retranscrites dans un document de traitement de texte (de type .docx) ou directement au sein du logiciel d'aide à l'analyse qualitative Nvivo Pro 13®, puis seront analysées via le logiciel Nvivo Pro 13®.

11.3.3 Codage des donnees

Les traitements prescrits et les événements cliniques sont codés dans l'eCRF afin de pouvoir effectuer le contrôle et l'analyse des données.

Les dictionnaires suivants sont utilisés pour le codage des termes médicaux :

- MedDRA (version en cours) FR/US,
- ATC version.

L'équipe investigatrice UroCCR est en charge du codage des données, sous la responsabilité de l'investigateur.

11.3.4 Controles des donnees

Des contrôles sont programmés afin de vérifier la cohérence et la complétude des données saisies dans l'eCRF. La liste des contrôles à mettre en place est définie conjointement entre l'investigateur coordonnateur et l'USMR, dans le plan de validation des données de l'étude.
Le DM d'UroCCR et l'ARC coordonnateur sont responsables de la gestion des demandes de correction, qu'ils lancent régulièrement.
L'investigateur fait les corrections nécessaires à la résolution des demandes de corrections.

11.3.5 Transfert des donnees

Les transferts de données (envoi, réception) sont réalisés conformément à la procédure en vigueur à l'USMR. Les modalités de transfert de données doivent être définies dans le Plan de Data Management. Pour des raisons de sécurité, les fichiers de données sont pseudonymisés puis transférés via la plateforme sécurisée CIRRUS ou NextCloud. Dans le cadre de ce protocole :

- Les données pourront être transférées sur le serveur du CHU de Bordeaux (administré par la DSIN du CHU de Bordeaux) pour la réalisation de tâches par le CeDS.
- Un transfert des données est également prévu au laboratoire CeDS (Université de Bordeaux) pour des analyses complémentaires des données qualitatives.

D'autres transferts de données peuvent être demandés et approuvés selon la procédure en vigueur à l'USMR.

11.4 CONFIDENTIALITE DES DONNEES

Conformément aux dispositions législatives en vigueur, les personnes ayant un accès direct aux données source prendront toutes les précautions nécessaires en vue d'assurer la confidentialité des informations relatives aux médicaments expérimentaux, aux recherches, aux personnes qui s'y prêtent et notamment en ce qui concerne leur identité ainsi qu'aux résultats obtenus. Ces personnes, au même titre que les investigateurs eux-mêmes, sont soumises au secret professionnel.

Pendant la recherche ou à son issue, les données recueillies sur les personnes qui s'y prêtent et transmises au promoteur par les investigateurs (ou tous autres intervenants spécialisés) seront pseudonymisées. Elles ne doivent en aucun cas faire apparaître en clair les noms des personnes concernées ni leurs adresses.

Chaque participant se verra attribuer un code confidentiel d'identification composé d'un numéro de participant (3 chiffres).

Le promoteur s'assurera que chaque personne qui se prête à la recherche a donné son accord par écrit pour l'accès aux données individuelles la concernant et strictement nécessaires au contrôle de qualité de la recherche.

11.5 CONSERVATION DES DOCUMENTS ET DES DONNEES RELATIFS A LA RECHERCHE

Les documents suivants relatifs à cette recherche sont archivés par l'investigateur conformément aux Bonnes Pratiques Cliniques, à l'arrêté du 11 août 2008 fixe la durée de conservation des documents relatifs aux recherches en santé et au règlement européen sur les médicaments:

- pour une durée de 20 ans suivant la fin de la recherche (recherches ne portant pas sur un produit mentionné à l'article L.5311-1 du code de la santé publique),
  - Le protocole et les modifications éventuelles au protocole
  - Les cahiers d'observation (copies papiers ou électroniques)
  - Les dossiers source des participants ayant signé un consentement
  - Tous les autres documents et courriers relatifs à la recherche
- pour une durée de 30 ans suivant la fin de la recherche,
  - L'exemplaire original des consentements éclairés signés des participants

Tous ces documents sont sous la responsabilité de l'investigateur pendant la durée réglementaire d'archivage.

Aucun déplacement ou destruction ne pourra être effectué sans l'accord du promoteur. Au terme de la durée réglementaire d'archivage, le promoteur sera consulté pour destruction. Toutes les données, tous les documents et rapports pourront faire l'objet d'audit ou d'inspection.

Les données recueillies pour l'étude seront accessibles aux personnes habilitées par le CHU de Bordeaux, pendant deux ans après la dernière publication des résultats de la recherche. Elles feront l'objet d'un archivage pendant 20 ans après la fin de l'étude conformément à la réglementation en vigueur.

11.6 CESSION DES DONNEES

La gestion des données est assurée par le CeDS. Les conditions de cession de tout ou partie de la base de données de la recherche sont décidées par le promoteur de la recherche et font l'objet d'un contrat écrit.

12. CONTROLE ET ASSURANCE QUALITE

12.1 ACCES AUX DONNEES

L'acceptation de la participation au protocole implique que les investigateurs mettront à disposition les documents et données individuelles strictement nécessaires au suivi, au contrôle de qualité et à l'audit de la recherche, à la disposition des personnes ayant un accès à ces documents conformément aux dispositions législatives et réglementaires en vigueur.

12.2 CONTROLE QUALITE

Le contrôle qualité sera effectué par un attaché de recherche clinique mandaté par le promoteur conformément au plan de monitorage basé sur le risque (logistique, impact, ressources) défini pour la recherche.

Ce dernier défini la nature des éléments à vérifier, les modalités et le rythme de visite dans le centre investigateur.

Toute visite fera l'objet d'un rapport de monitorage par compte-rendu écrit transmis à l'investigateur principal du centre.

12.3 AUDIT ET INSPECTION

Un audit peut être réalisé à tout moment par des personnes mandatées par le promoteur et indépendantes des personnes menant la recherche. Il a pour objectif de vérifier la sécurité des participants et le respect de leurs droits, le respect de la réglementation applicable et la fiabilité des données.

Une inspection peut également être diligentée par une autorité compétente (ANSM pour la France ou autre autorité réglementaire dans le cadre d'une recherche européenne par exemple).

L'audit, aussi bien que l'inspection, pourront s'appliquer à tous les stades de la recherche, du développement du protocole à la publication des résultats et au classement des données utilisées ou produites dans le cadre de la recherche.

Les investigateurs acceptent de se conformer aux exigences du promoteur en ce qui concerne un audit et à l'autorité compétente pour une inspection de la recherche.

13. CONSIDERATIONS ETHIQUES ET REGLEMENTAIRES

PRENDRE CONTACT AVEC LE PROMOTEUR POUR AIDE A LA REDACTION DE CE CHAPITRE

13.1 CONFORMITE AUX TEXTES DE REFERENCE

Le promoteur et l'(es) investigateur(s) s'engagent à ce que cette recherche soit réalisée en conformité avec la loi n°2012-300 du 5 mars 2012 relative aux recherches impliquant la personne humaine, ainsi qu'en accord avec les Bonnes Pratiques Cliniques (I.C.H. E6 (R2) du 1er décembre 2016 et décision du 24 novembre 2006) et la déclaration d'Helsinki (qui peut être retrouvée dans sa version intégrale sur le site www.wma.net).

La recherche est conduite conformément au présent protocole. Hormis dans les situations d'urgence nécessitant la mise en place d'actes thérapeutiques précis, l'(es) investigateur(s) s'engage(nt) à respecter le protocole en tous points.

Cette recherche a reçu l'avis favorable du Comité de Protection des Personnes (CPP) Sud Est IV et a fait l'objet d'une information auprès de l'ANSM.

Le CHU de Bordeaux promoteur de cette recherche, a souscrit un contrat d'assurance en responsabilité civile auprès de Lloyd's Insurance Company SA (représentée par BEAH, mandataire) conformément aux dispositions du code de la santé publique.

Les données nécessaires à cette recherche sont enregistrées dans la base de données UroCCR qui a obtenu l'autorisation de la Commission Nationale de l'Informatique et des Libertés (CNIL) en date du 12/04/2013 (demande d'autorisation n°912578, décision DR-2013-206). Les données de la base UroCCR font l'objet d'un traitement informatisé au CREDIM conformément aux dispositions de la loi relative à l'informatique, aux fichiers et aux libertés (loi n° 78-17 du 6 janvier 1978 relative à l'informatique, aux fichiers et aux libertés modifiée par la loi n° 2018-493 du 20 juin 2018 relative à la protection des données personnelles) et au règlement général sur la protection des données (règlement UE 2016/679).

Cette recherche entre dans le cadre de la « Méthodologie de référence » MR-001 en application des dispositions de l'article 54 de la loi du 6 janvier 1978 modifiée relative à l'information, aux fichiers et aux libertés. Le CHU de Bordeaux, l'USMR du CHU de Bordeaux ont signé un engagement de conformité à cette « Méthodologie de référence ».

Cette recherche est enregistrée dans la base ID-RCB sous le n°2024-A00129-38. Cette

recherche est enregistrée sur le site http://clinicaltrials.gov/

13.2 MODIFICATIONS AU PROTOCOLE

Toute modification substantielle, c'est à dire toute modification de nature à avoir un impact significatif sur la protection des personnes, sur les conditions de validité et sur les résultats de la recherche, sur la qualité et la sécurité des produits expérimentés, sur l'interprétation des documents scientifiques qui viennent appuyer le déroulement de la recherche ou sur les modalités de conduite de celle-ci, fait l'objet d'un amendement écrit qui est soumis au promoteur ; celui-ci doit obtenir, préalablement à sa mise en œuvre, un avis favorable du CPP.

Les modifications non substantielles, c'est à dire celles n'ayant pas d'impact significatif sur quelque aspect de la recherche que ce soit, sont communiquées au CPP à titre d'information.

Toutes les modifications sont validées par le promoteur, et par tous les intervenants de la recherche concernés par la modification, avant soumission au CPP. Cette validation peut nécessiter la réunion de tout comité constitué pour la recherche.

Toutes les modifications au protocole doivent être portées à la connaissance de tous les investigateurs qui participent à la recherche. Les investigateurs s'engagent à en respecter le contenu.
Toute modification qui modifie la prise en charge des participants ou les bénéfices, risques et contraintes de la recherche fait l'objet d'une nouvelle note d'information et d'un nouveau formulaire de consentement dont le recueil suit la même procédure que celle précitée.

14. RAPPORT FINAL

Dans un délai d'un an suivant la fin de la recherche ou son interruption, un rapport final sera établi et signé par le promoteur et l'investigateur. Ce rapport sera tenu à la disposition de l'autorité compétente. Le promoteur transmettra au CPP et, le cas échéant, à l'ANSM les résultats de la recherche sous forme d'un résumé du rapport final dans un délai d'un an après la fin de la recherche.

15. REGLES RELATIVES A LA PUBLICATION

15.1 COMMUNICATIONS SCIENTIFIQUES

L'édition du rapport final est réalisée par le CeDS avec l'apport d'expertise de l'USMR sur les questions méthodologiques autour des données quantitatives. Cette analyse donne lieu à un rapport écrit qui est soumis au promoteur, qui transmettra au Comité de Protection des Personnes et à l'autorité compétente.

Toute communication écrite ou orale des résultats de la recherche doit recevoir l'accord préalable de l'investigateur coordonnateur et, le cas échéant, de tout comité constitué pour la recherche.

L'investigateur coordonnateur s'engage à mettre à disposition du public les résultats de la recherche aussi bien négatifs et non concluants que positifs.

La publication des résultats principaux mentionne le CHU de Bordeaux, tous les investigateurs ayant inclus ou suivi des participants dans la recherche, des méthodologistes, biostatisticiens et data managers ayant participé à la recherche, des membres du(des) comité(s) constitué(s) pour la recherche et la mention « ce travail a bénéficié d'une aide de l'Etat gérée par l'Agence Nationale de la Recherche au titre du troisième PIA intégré à France 2030 portant la référence ANR-21-RHUS-0015 ».

Pour les publications en SHS, ces règles s'appliquent aux remerciements avec la mention « ce travail a bénéficié d'une aide de l'Etat gérée par l'Agence Nationale de la Recherche au titre du troisième PIA intégré à France 2030 portant la référence ANR-21-RHUS-0015 ». Il sera tenu compte des règles internationales d'écriture et de publication (The Uniform Requirements for Manuscripts de l'ICMJE, avril 2010) concernant la qualité d'auteurs.

15.2 COMMUNICATION DES RESULTATS AUX PARTICIPANTS

Conformément à la loi n°2002-303 du 4 mars 2002, les participants sont informés, à leur demande, des résultats globaux de la recherche.

RÉFÉRENCES BIBLIOGRAPHIQUES

1. Bernhard JC, Isotani S, Matsugasumi T, Duddalwar V, Hung AJ, Suer E, et al. Personalized 3D printed model of kidney and tumor anatomy: a useful tool for patient education. World J Urol. mars 2016;34(3):337-45.

2. Travaline JM, Ruchinskas R, D'Alonzo GE. Patient-physician communication: why and how. J Am Osteopath Assoc. janv 2005;105(1):13-8.

3. Curchod C. 1 - Identifier les facteurs de détérioration de la relation. In: Curchod C, éditeur. Relations soignants–soignés [Internet]. Paris: Elsevier Masson; 2009 [cité 3 août 2023]. p. 13-28. Disponible sur: https://www.sciencedirect.com/science/article/pii/B9782294705373500017

4. Rey S, Leduc A, Debussche X, Rigal L, Ringa V. Une personne sur dix éprouve des difficultés de compréhension de l'information médicale. Etudes Résultats. 2023;1269:8.

5. Stephanie CJ, Mathieu A, Aurore M, Monique MRT. Outpatients' perception of their preoperative information regarding their health literacy skills and their preoperative anxiety level: Protocol for a prospective multicenter cross-sectional study. Medicine (Baltimore). 21 mai 2021;100(20):e26018.

6. Köhler H, Dorozhkina R, Gruner-Labitzke K, de Zwaan M. Specific Health Knowledge and Health Literacy of Patients before and after Bariatric Surgery: A Cross-Sectional Study. Obes Facts. 2020;13(2):166-78.

7. Hälleberg Nyman M, Nilsson U, Dahlberg K, Jaensson M. Association Between Functional Health Literacy and Postoperative Recovery, Health Care Contacts, and Health-Related Quality of Life Among Patients Undergoing Day Surgery. JAMA Surg. août 2018;153(8):738-45.

8. Shen HN, Lin CC, Hoffmann T, Tsai CY, Hou WH, Kuo KN. The relationship between health literacy and perceived shared decision making in patients with breast cancer. Patient Educ Couns. 1 févr 2019;102(2):360-6.

9. Pieterse AH, Gulbrandsen P, Ofstad EH, Menichetti J. What does shared decision making ask from doctors? Uncovering suppressed qualities that could improve person-centered care. Patient Educ Couns. 1 sept 2023;114:107801.

10. Luckenbaugh AN, Moses KA. The impact of health literacy on urologic oncology care. Urol Oncol. avr 2022;40(4):117-9.

11. Marche H. Au-delà de l'autonomie du patient : l' « esprit de soin » dans les trajectoires de cancer avancé. Anthropol Santé Rev Int Francoph Anthropol Santé [Internet]. 11 mai 2015 [cité 11 mai 2023];(10). Disponible sur: https://journals.openedition.org/anthropologiesante/1595

12. Canfell OJ, Meshkat Y, Kodiyattu Z, Engstrom T, Chan W, Mifsud J, et al. Understanding the Digital Disruption of Health Care: An Ethnographic Study of Real-Time Multidisciplinary Clinical Behavior in a New Digital Hospital. Appl Clin Inform. 9 nov 2022;13(5):1079-91.

13. Scott ER, Singh A, Quinn A, Boyd K, Lallas CD. How I Do It: Cost-effective 3D printed models for renal masses. Can J Urol. oct 2021;28(5):10874-7.

14. Sørensen K, Van den Broucke S, Pelikan JM, Fullam J, Doyle G, Slonska Z, et al. Measuring health literacy in populations: illuminating the design and development process of the European Health Literacy Survey Questionnaire (HLS-EU-Q). BMC Public Health. 10 oct 2013;13(1):948.

15. Rouquette A, Nadot T, Labitrie P, Van den Broucke S, Mancini J, Rigal L, et al. Validity and measurement invariance across sex, age, and education level of the French short versions of the European Health Literacy Survey Questionnaire. PloS One. 2018;13(12):e0208091.

16. Kaufmann JC. Introduction. In: L'entretien compréhensif [Internet]. Paris: Armand Colin; 2016 [cité 15 nov 2022]. p. 9-11. (128; vol. 4e éd.). Disponible sur: https://www.cairn.info/l-entretiencomprehensif--9782200613976-p-9.htm

17. Fainzang S. La relation médecins-malades: information et mensonge. (France): Paris : PUF; 2006. 159 p. (Ethnologies).

18. Pierron JP. Une nouvelle figure du patient ? Les transformations contemporaines de la relation de soins. Sci Soc Santé. 2007;25(2):43-66.

19. Renault L. L'analyse qualitative entre disciplines. Polit Soc. 2020;1-2(1):43-53.

20. Morse JM. Approaches to Qualitative-Quantitative Methodological Triangulation. Nurs Res. avr 1991;40(2):120.

21. Balard F, Kivits J, Schrecker C, Volery. L’analyse qualitative en santé. In: Les recherches qualitatives en santé. Malakoff: Armand Colin; 2016. (Collection U).

ANNEXES

Annexe 1 : ECHELLE Littératie HLS-EU16

HLS-EU16 version Française (Version 2020)

Indiquez, sur une échelle de très facile à très difficile, dans quelle mesure il est facile pour vous de...

	très facile	facile	difficile	très difficile
... trouver des informations sur les traitements des maladies qui vous concernent ?	❑	❑	❑	❑
... savoir où obtenir l'aide d'un professionnel quand vous êtes malade ? (Par ex. médecin, infirmier, pharmacien ou psychologue)	❑	❑	❑	❑
... comprendre ce qu'un médecin vous dit ?	❑	❑	❑	❑
... comprendre les consignes de votre médecin ou pharmacien sur la manière de prendre vos médicaments ?	❑	❑	❑	❑
... savoir quand il serait utile d'avoir l'avis d'un autre médecin ?	❑	❑	❑	❑
... utiliser les informations que le médecin vous donne pour prendre des décisions concernant votre maladie ?	❑	❑	❑	❑
... suivre les consignes de votre médecin ou pharmacien ?	❑	❑	❑	❑
... trouver des informations sur comment faire en cas de problèmes psychologiques ? (Par ex. stress, dépression ou anxiété)	❑	❑	❑	❑
... comprendre les mises en gardes concernant l'impact sur la santé de certains comportements comme fumer, ne pas faire assez d'exercice et boire trop ?	❑	❑	❑	❑

Tournez la page s'il vous plaît...

Indiquez, sur une échelle de très facile à très difficile, dans quelle mesure il est facile pour vous de...

	très facile	facile	difficile	très difficile
... comprendre les informations sur les dépistages et examens recommandés ? (Par ex. dépistage du cancer colorectal, test de glycémie)	❑	❑	❑	❑
... évaluer la fiabilité des informations disponibles dans les médias sur ce qui est dangereux pour la santé ? (Par ex. journaux, télévision ou internet)	❑	❑	❑	❑
... savoir comment vous protéger des maladies à partir des informations disponibles dans les médias ? (Par ex. journaux, télévision ou internet)	❑	❑	❑	❑
... vous renseigner sur les activités bénéfiques pour votre santé et votre bien être ? (Par ex. relaxation, exercice physique, yoga)	❑	❑	❑	❑
... comprendre les conseils de votre famille ou de vos amis en matière de santé ?	❑	❑	❑	❑
... comprendre les informations disponibles dans les médias pour être en meilleure santé ?	❑	❑	❑	❑
... identifier quels sont les comportements de votre vie de tous les jours qui ont un impact sur votre santé ?	❑	❑	❑	❑

Fin du questionnaire.

Version courte du « European Health Literacy Survey Questionnaire » (Sørensen et al., 2013), validée en français par Rouquette en 2018 (Rouquette et al., 2018).
Disponible sur : https://reflis.fr/wp-content/uploads/2020/07/HLSEU16-Francais-2020.pdf

Annexe 2 – Questionnaire de l'étude Bernhard (2016)

1/ What I know about the kidney, generally speaking	1/ Ce que je sais sur le rein, de manière générale	True / Vrai	False / Faux	Don't know / je ne sais pas
1.1- The kidney is a paired organ	1.1- Le rein est un organe pair			
1.2- The kidney function is to "clean" my blood	1.2- La fonction du rein est d'épurer le sang			
1.3- The kidney produces urines	1.3- Le rein produit l'urine			
1.4- The urine is collected in cavities inside of the kidney, called the collecting system	1.4- L'urine est recueillie dans des cavités situées à l'intérieur du rein, appelées système collecteur.			
1.5- The kidney is a highly vascularized organ; a lot of blood lows through the renal vessels	1.5- Le rein est un organe très vascularisé, avec un débit sanguin important			
1.6- The renal vessels can be described as an artery and a vein	1.6- Les vaisseaux du rein comprennent l'artère et la veine rénales			
1.7- To work properly the kidney needs blood coming through the renal artery	1.7- Pour fonctionner correctement, le rein a besoin de sang qui arrive par l'artère rénale			
1.8- When the kidneys are not working properly this leads to renal insufficiency	1.8- Lorsque les reins ne fonctionnent pas correctement, cela conduit à l'insuffisance rénale			
**2/ What I know about my disease:**	**2/ Ce que je sais de ma maladie :**	**True / Vrai**	**False / Faux**	**Don't know / je ne sais pas**
2.1- My kidney is bearing a tumor	2.1- Mon rein est porteur d'une tumeur			
2.2- The tumor is located in the mid part of my kidney	2.2- La tumeur est située dans la partie médiane de mon rein			
2.3- The tumor is in close contact with the kidney vessels	2.3- La tumeur est en contact étroit avec les vaisseaux du rein			
2.4- The tumor is in close contact with the collecting system	2.4- La tumeur est en contact étroit avec le système collecteur			
**3/ What I understand about my planned surgery**	**3/ Ce que je comprends de l'intervention chirurgicale prévue**	**True / Vrai**	**False / Faux**	**Don't know / je ne sais pas**
3.1- My surgeon will try to remove the tumor only	3.1- Mon chirurgien essaiera d'enlever uniquement la tumeur			
3.2- My surgeon will remove the entire kidney	3.2- Mon chirurgien va enlever tout le rein			
In case of tumor only removal my surgeon will have to cut the kidney itself to separate the tumor from surrounding healthy tissue. This may lead to:<br>3.3- bleeding with a risk of hemorrhage	Dans le cas d'une ablation de la tumeur uniquement, mon chirurgien devra couper le rein lui-même pour séparer la tumeur des tissus sains qui l'entourent. Cela peut entraîner<br>3.3- des saignements avec un risque d'hémorragie			
3.4- opening the collecting system with a risk of urine leakage	3.4- l'ouverture du système collecteur avec un risque de fuite d'urine			

3.5- To reduce the risk of hemorrhage at the time of tumor removal my surgeon may need to clamp (=interrupt blood-low) the renal artery	3.5- Pour réduire le risque d'hémorragie au moment de l'ablation de la tumeur, mon chirurgien peut être amené à clamper (= interrompre le flux sanguin) l'artère rénale.			
3.6- Prolonged renal artery clamping is known to alter renal function so my surgeon will have to speed up the procedure to limit the length of clamping	3.6- Le clampage prolongé de l'artère rénale est connu pour altérer la fonction rénale, mon chirurgien devra donc accélérer la procédure pour limiter la durée du clampage			
3.7- In case of tumor only removal, the benefit is preservation of healthy kidney tissue	3.7- En cas d'ablation de la tumeur uniquement, l'avantage est la préservation du tissu rénal sain.			
3.8- Preserving healthy tissue from my tumor bearing kidney decreases the risk of renal insuficiency	3.8- Préserver le tissu sain de mon rein porteur de tumeur diminue le risque d'insuffisance rénale			

Annexe 3 – Questionnaire de satisfaction (adapté de l'étude Bernhard 2016)

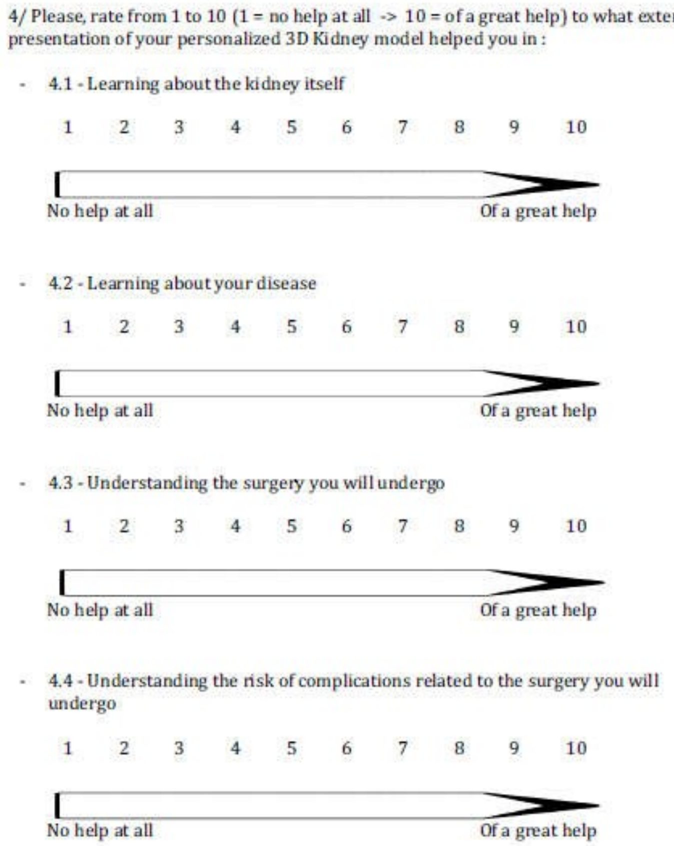
4/ Please, rate from 1 to 10 (1 = no help at all -> 10 = of a great help) to what extent the presentation of your personalized 3D Kidney model helped you in :

- 4.1 - Learning about the kidney itself

  1 2 3 4 5 6 7 8 9 10

  No help at all — Of a great help

- 4.2 - Learning about your disease

  1 2 3 4 5 6 7 8 9 10

  No help at all — Of a great help

- 4.3 - Understanding the surgery you will undergo

  1 2 3 4 5 6 7 8 9 10

  No help at all — Of a great help

- 4.4 - Understanding the risk of complications related to the surgery you will undergo

  1 2 3 4 5 6 7 8 9 10

  No help at all — Of a great help

4/ S'il vous plaît, notez de 1 à 10 (1 = pas d'aide du tout -> 10 = d'une grande aide) dans quelle mesure la présentation du modèle de Rein imprimé en 3D vous a aidé à :

- 4.1 – Apprendre « des notions » sur le rein lui-même

  De 1 à 10, 1 signifie ne vous a pas aidé, 10 vous a été d'une grande aide

- 4.2 – Comprendre votre maladie ;

  De 1 à 10, 1 signifie ne vous a pas aidé, 10 vous a été d'une grande aide

- 4.3 - Comprendre la chirurgie prévue,

  De 1 à 10, 1 signifie ne vous a pas aidé, 10 vous a été d'une grande aide

- 4.4 - Comprendre le risque de complications liées à l'intervention chirurgicale prévue

  De 1 à 10, 1 signifie ne vous a pas aidé, 10 vous a été d'une grande aide
